# Phylogenomic analysis and development of molecular markers for the determination of twelve plum cultivars (*Prunus*, Rosaceae)

**DOI:** 10.1186/s12864-022-08965-z

**Published:** 2022-11-08

**Authors:** Yicen Xu, Bo Fang, Jingling Li, Yuanwei Wang, Jingting Liu, Chang Liu, Jie Yu

**Affiliations:** 1 College of Horticulture and Landscape Architecture, Southwest University, Chongqing, 400715 China; 2 Chongqing Academy of Agricultural Sciences, Chongqing, 401329 China; 3 Improved Seed Farm in Liangping District, Chongqing, 405299 China; 4 Institute of Medicinal Plant Development, Chinese Academy of Medical Sciences, Peking Union Medical College, Beijing, 100193 China

**Keywords:** Prunus, Plastome, Phylogenetic analysis, Molecular markers

## Abstract

**Background:**

Plums are one of the most important economic crops of the Rosaceae family and are produced all over the world. China has many local varieties, but the genomic information is limited for genetic studies. Here, we first sequenced, assembled, and analyzed the plastomes of twelve plum cultivars and developed molecular markers to distinguish them.

**Results:**

The twelve plastomes of plum cultivars have a circular structure of 157,863–157,952 bp containing a large single-copy region (LSC) of 86,109–86,287 bp, a small copy region (SSC) of 18,927–19,031 bp, and two inverted repeats (IR) of 26,353–26,387 bp each. The plastomes of plum cultivars encode 131 genes, including 86 protein-coding genes, 37 tRNA genes, and 8 rRNA genes. We detected 50, 54, 54, 53, 53, 50, 54, 54, 54, 49, 50, 54 SSRs in the twelve analyzed varieties, respectively. For repeat sequences, we identified 553 tandem repeats, 204 direct repeats, and 270 palindromic repeats. We also analyzed the expansion/contraction of IR regions. The genes *rpl*22, *rps*19, *rpl*2, *ycf*1, *ndh*F, and the *trn*H span on or near the boundary of IR and single-copy regions. Phylogenetic analysis showed that the twelve cultivars were clustered with the *P. salicina* and *P. domestica*. We developed eight markers LZ01 to LZ08 based on whole plastomes and nuclear genes and validated them successfully with six repetitions.

**Conclusions:**

The results obtained here could fill in the blanks of the plastomes of these twelve plum cultivars and provide a wider perspective based on the basis of the plastomes of *Prunus* to the molecular identification and phylogenetic construction accurately. The analysis from this study provides an important and valuable resource for studying the genetic basis for agronomic and adaptive differentiation of the *Prunus* species.

**Supplementary Information:**

The online version contains supplementary material available at 10.1186/s12864-022-08965-z.

## Background

Plums are one of the most economically important crops of the Rosaceae family and are cultivated all over the world. Plums are one of the most important stone fruits consumed worldwide because of their high degree of acceptance by consumers [1]. Plums are rich in vitamin C, vitamin E, non-essential and essential amino acids, total phenols, flavonoids, and trace elements such as potassium, calcium, and magnesium. They have good antioxidant activity, and the active peptide extracted from it can effectively resist ABTS (2, 2'-azino-bis(3-ethylbenzothiazoline-6-sulfonic acid)) free radical and inhibit angiotensin-converting enzyme [2]. As a result, plums are widely recognized for their nutritional and economic value.

There are about 19 to 40 species of plums distributed across Asia, Europe, and North America [3, 4]. China is the original distribution center of Chinese plums, which are widely used. There are local varieties that have been cultivated for a long time in various regions of the country, such as the Wushan plum [5], the Fendai plum [6] and the Wanshuang plum [7] in Chongqing, the Sanhua plum [8] in Guangdong, Cuihong plum [9] and Jiangan plum [10] in Sichuan, the Fengtang plum [11] in Guizhou, Hongxin plum [12] in Zhejiang, among other regions. These fruits are mostly consumed while fresh because of their characteristic taste [13].

In recent years, the development of genomics and high-throughput sequencing technology has provided strong support for the study of plant plastomes. The interest in plant plastomes has increased since 1986, when the first whole plastomes were published for *Nicotiana tabacum* [14] and *Marchantia polymorpha* [15]. Compared with the nuclear genome, the plastome is characterized by small molecular weight, single copy, simple structure, highly conserved gene structure order and gene content, and low gene substitution rate [16, 17]. The plastome is uniparental, with gymnosperms inherited on the paternal line and angiosperms on the maternal line, therefore it will not be disturbed by genetic recombination; the evolutionary path of the plastome is relatively independent, and does not depend on other data to construct a phylogenetic tree [18]. So, the plastome is widely used in plant phylogeny and evolution, species identification, and taxonomy.

Lately, DNA markers were developed to authenticate *Prunus* genus. For example, Yamamoto T (2003) has analyzed peach germplasm resources using SSR markers and found that Japanese peaches are closely related to Chinese peaches [19]. Ortiz used the RAPD technique to detect hexaploid and diploid plum cultivars, and only three random primers were used to distinguish 31 plum varieties [20]. Twenty four Chinese plum varieties are proved from three types of production areas using 16 SSR primer pairs [21].

However, to date, genetic information is scarce, preventing in-depth molecular breeding. In this study, we selected twelve plum varieties that are most consumed in China, including Chinese plums: ‘Sanhua plum’, ‘Wanshuang plum’, ‘Wuyuecui’, ‘Oishiwase’, ‘Yinhong plum’, ‘Fengtang plum’, ‘Cuihong plum’, and ‘No.2 Guofeng’; European plums: ‘Richard Early’, ‘Bingtang plum’; *Prunus cerasifera* 'Hollywood' and *Prunus simonii* ‘Weiwang’. Our goal is to understand their taxonomic relationship and to develop high-resolution molecular markers for discrimination.

## Results

### General features of the twelve plastomes

Using Illumina NovaSeq 6000 sequencing platforms, we obtained 5.01 – 6.21 G clean data from each plum cultivar and the number of clean reads ranged from 16,709,174 to 20,713,829 (Table S1). The twelve plastomes of plum cultivars have a circular structure of 157,863–157,952 bp containing a large single-copy region (LSC) of 86,109–86,287 bp, a small copy region (SSC) of 18,927–19,031 bp, and two inverted repeats (IR) of 26,353–26,387 bp by each. In general, there were small differences in the length of plastomes of the plants in this study. The GC content analysis showed that the total GC content ranged from 36.72% to 36.76% in the twelve plastomes. The GC contents in IR regions (42.58%-42.62%) are significantly higher than those in LSC (34.51%-34.59%) and SSC regions (30.36%-30.54%) (Table 1). Since the cultivars belong to the same genus, there is little difference in GC content. The twelve plastomes were deposited to NCBI (Accession number: MW406457, MW406459, MW406460, MW406461, MW406463, MW406464, MW406465, MW406466, MW406468, MW406470, MW406471, MW406472).

**Table 1:** Basic characteristics of plastomes of twelve varieties of *Prunus*

Gene numbers				GC content (%)				Length (bp)				Accession number	Varieties
rRNA gene	tRNA gene	Protein-coding gene	Total	SSC	IR	LSC	Total	IR	SSC	LSC	Total		
8	37	86	131	30.38	42.62	34.52	36.73	26,353	19,027	86,130	157,863	MW406459	*P. salicina* ‘Sanhua plum’
8	37	86	131	30.37	42.62	34.51	36.72	26,353	19,031	86,189	157,926	MW406460	*P. salicina* ‘Wanshuang plum’
8	37	86	131	30.4	42.58	34.59	36.76	26,387	19,022	86,109	157,905	MW406461	*P. salicina* ‘Wuyuecui’
8	37	86	131	30.39	42.59	34.58	36.75	26,382	19,029	86,123	157,916	MW406457	*P. salicina* ‘Oishiwase’
8	37	86	131	30.37	42.62	34.51	36.72	26,353	19,031	86,187	157,924	MW406463	*P. simonii* ‘Weiwang’
8	37	86	131	30.48	42.59	34.54	36.74	26,379	18,964	86,213	157,935	MW406464	*P. domestica* ‘Richard Early’
8	37	86	131	30.37	42.62	34.51	36.72	26,353	19,031	86,176	157,913	MW406465	*P. salicina* ‘Yinhong plum’
8	37	86	131	30.36	42.62	34.51	36.72	26,353	19,031	86,189	157,926	MW406466	*P. salicina* ‘Fengtang plum’
8	37	86	131	30.37	42.62	34.51	36.72	26,354	19,031	86,190	157,929	MW406468	*P. salicina* ‘Cuihong plum’
8	37	86	131	30.54	42.61	34.52	36.74	26,369	18,927	86,287	157,952	MW406470	*P. cerasifera* ‘Hollywood’
8	37	86	131	30.48	42.59	34.54	36.74	26,379	18,964	86,213	157,935	MW406471	*P. domestica* ‘Bingtang plum’
8	37	86	131	30.4	42.58	34.59	36.76	26,387	19,022	86,109	157,905	MW406472	*P. salicina* ‘No.2 Guofeng’

### Genome annotation

**Fig. 1:**
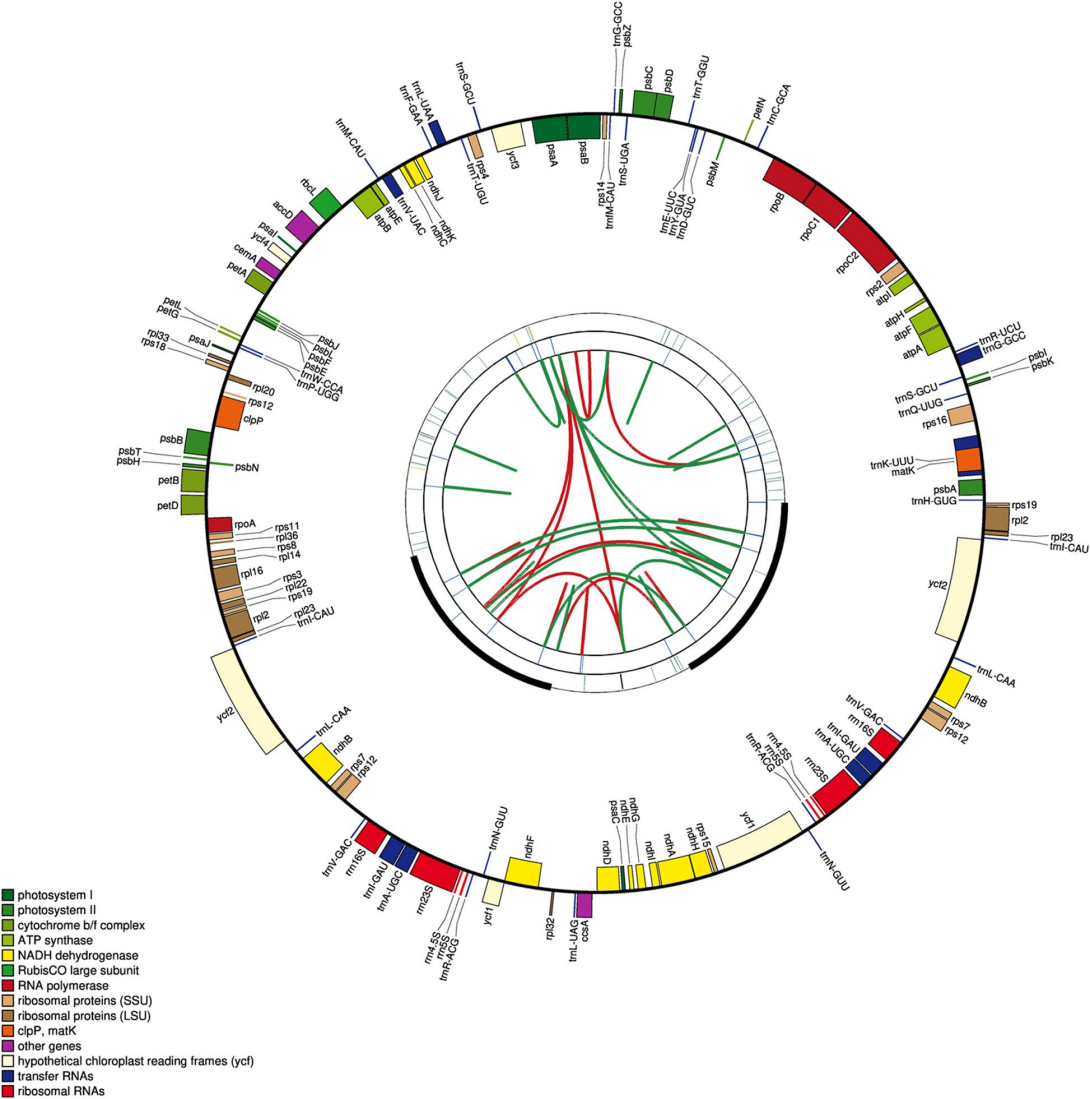
Genome map of *P. salicina* ‘Sanhua plum’ plastome. The map has four rings, from the center outward, with red and green arcs on the first circle connecting forward and reverse repeats, respectively; the second ring shows tandem repeats marked with dashes; the third ring is a MISA-identified microsatellite sequence; and the fourth ring shows the gene structure on the plastome. The colors of these genes are classified according to their function, as shown in the lower left corner

The plastomes of twelve plum cultivars all encoded 131 genes, among which, 110 are unique genes, including 78 protein-coding genes, 28 tRNA genes, and 4 rRNA genes (Table S2). An IR region contains 21 genes, four rRNA genes, nine tRNA genes, and eight protein-coding genes, respectively. The plastomes map is shown in Fig. 1 and Figures S1, S2, S3, S4, S5, S6, S7, S8, S9, S10, S11. Among the 78 protein-coding genes annotated, the genes containing two introns were the *ycf*3 and *clp*P genes, and the genes containing one intron include the *trn*K-UUU, *rps*16, *trn*G-UCC, *atp*F, *rpo*C1, *trn*L-UAA, *trn*V-UAC, *pet*B, *pet*D, *rpl*16, *ndh*A, and two repeats of *rpl*2, *ndh*B, *trn*I-GAU, *trn*A-UGC (Table S3).

### Repeats analysis

**Fig. 2:**
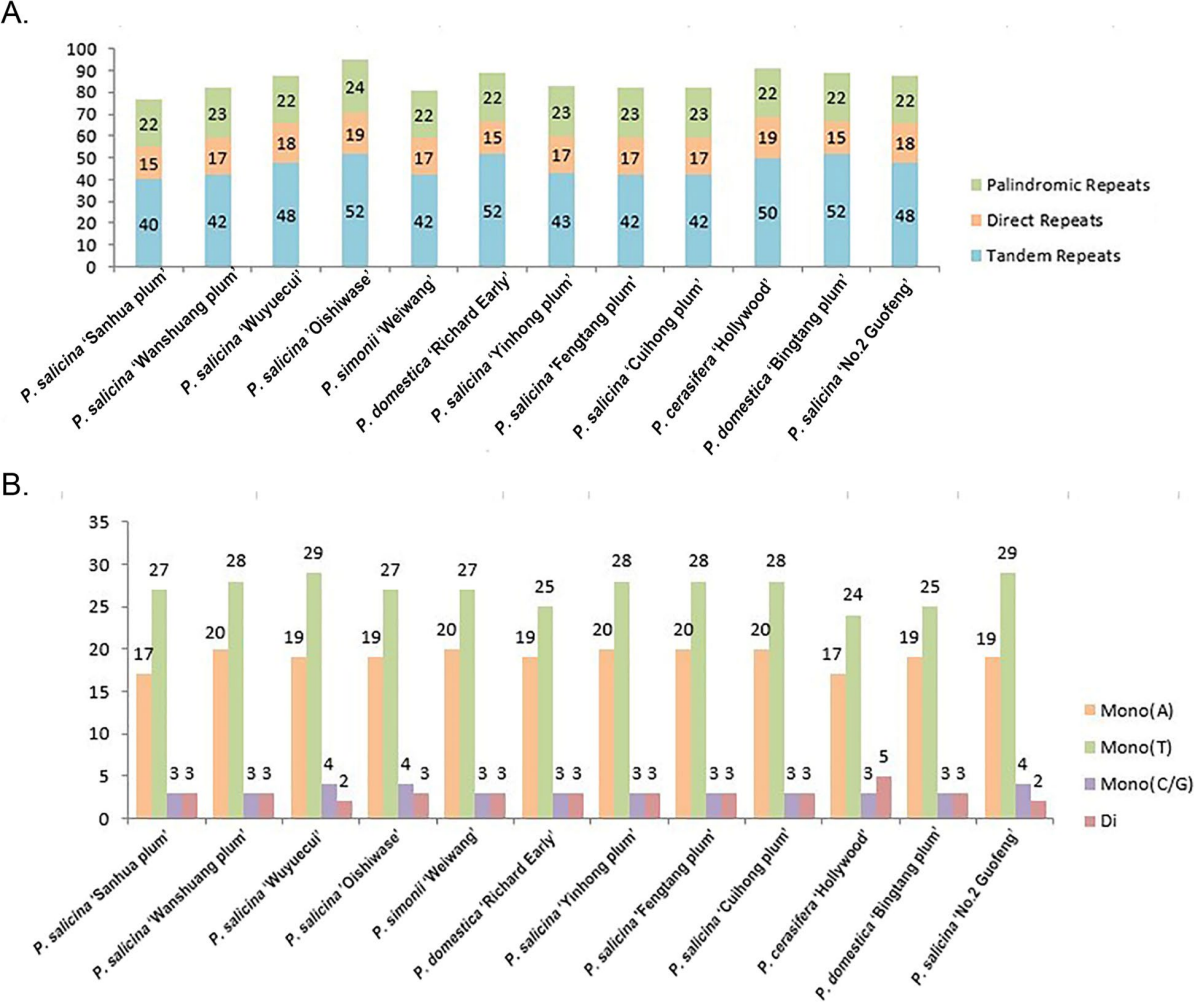
Comparison of the Repeats in the plastomes of 12 plum cultivars. A. Types and numbers of the interspersed repeats in the 12 plastomes; B. Types and numbers of SSRs detected in the 12 plastomes

In plastomes of twelve plum cultivars, we identified three kinds of repeated sequences including tandem repeats, direct repeats, and palindromic repeats. The numbers of them are 553, 204 and 270, respectively. Among them, *P. salicina* 'Oishiwase' has the most repeats (95) including 52 tandem repeats, 19 direct repeats and 24 palindromic repeats. On the contrary, *P. salicina* 'Sanhua plum' has the least repeats (77) including 40 tandem repeats, 15 direct repeats and 22 palindromic repeats (Fig. 2A).

Simple sequence repeats (SSRs), also known as microsatellite sequences, provide a large number of information about genetic variation. SSRs have high genetic polymorphism and are commonly used to develop molecular markers that play an important role in species identification. In this study, we detected 50, 54, 54, 53, 53, 50, 54, 54, 54, 49, 50, 54 SSRs in the twelve analyzed varieties, respectively (Fig. 2B, Table S4). Most SSRs are mononucleotide, particularly A/T motifs, which accounts for more than 80% of the total. Moreover, *P. cerasifera* ‘Hollywood’ has the least SSRs, but has the most dinucleotide. These SSRs have the potential in the identification of *Prunus*.

### Contraction and expansion analysis of IR regions

**Fig. 3:**
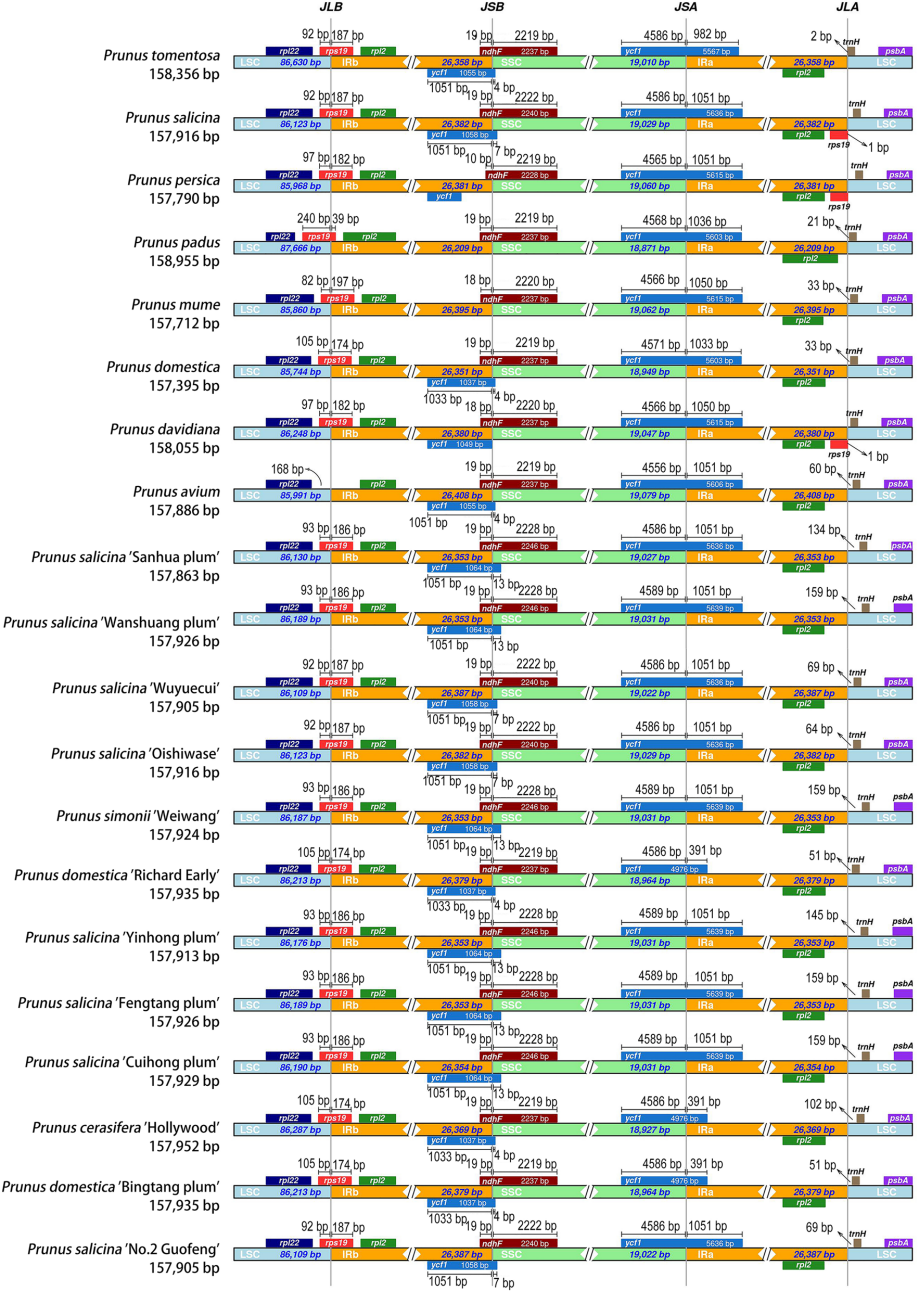
Comparison of the borders among LSC, SSC, and IR regions of twelve analyzed plums. The genes around the borders are shown above or below the mainline. The JLB, JSB, JSA, and JLA represent junction sites of LSC/IRb, IRb/SSC, SSC/IRa, and IRa/LSC, respectively

The IR regions of the twelve plum cultivars plastomes are the most conserved regions, being 26,353 to 26,387 bp in length. However, the expansions and contractions of the IR boundary can cause the diversity of plastome length [22]. The LSC/IR and SSC/IR borders of the *Prunus* plastomes were compared (Fig. 3). We observed several genes span on or near the boundary of IR and single-copy regions. These are mainly the genes *rpl*22, *rps*19, *rpl*2, *ycf*1, *ndh*F and *trn*H. Among them, *rps*19 gene span the LSC/IRb boundary, but the *P. domestica* ‘Richard Early’, *P. cerasifera* ‘Hollywood’ and *P. domestica* ‘Bingtang plum’s’ *rps*19 gene in IRb (174 bp) is shorter than the other nine varieties (186 bp/187 bp). Similarly, in terms of two copies of *ycf*1 gene span the IRb/SSC and SSC/IRa, the above three varieties are also shorter than the other nine. It can be seen that the genomic structure has changed in *Prunus*.

### Hypervariable Region Analysis

**Fig. 4:**
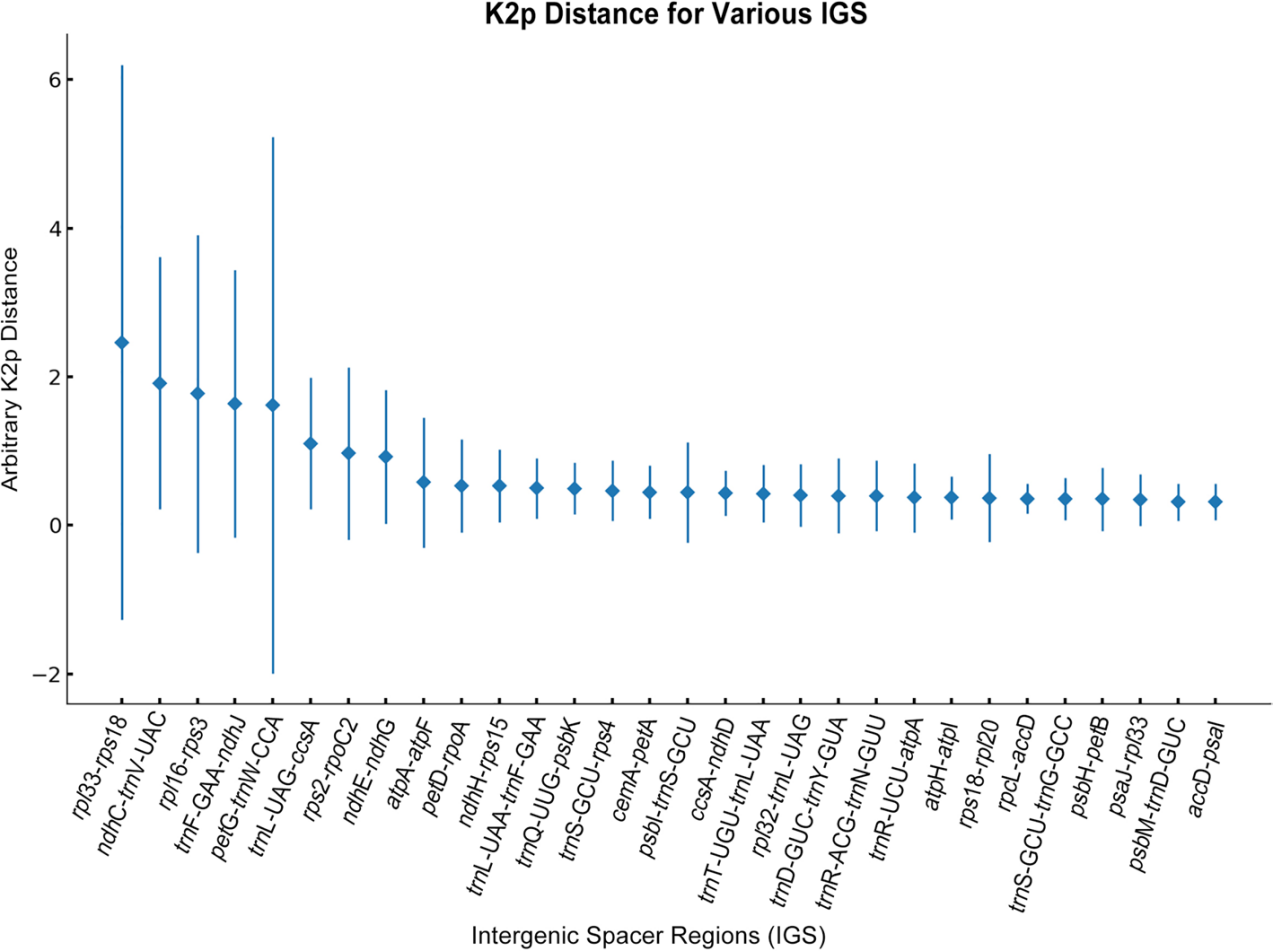
Comparison of the variability of IGS regions among the plastomes of 12 plums. The X-axis indicates the IGS regions, and the Y-axis shows the range of K2p distances between different pairs of species. The diamond shows the average K2p distance of the IGS region, respectively

Hypervariable regions can be used to resolve phylogenies and to discriminate closely related plant species [23]. The pairwise comparison of intergenic spacer regions was conducted to identify divergence hotspot regions among the twelve plum cultivars using the Kimura 2-parameter (K2p) model. The average K2p distance ranged from 0.00 to 2.463. The IGS regions of *rpl*33-*rps*18, *ndh*C-*trn*V-UAC, *rpl*16-*rps*3, *trn*F-GAA-*ndh*J, and *pet*G-*trn*W-CCA showed the largest distances of 2.463, 1.915, 1.772, 1.64 and 1.615, respectively (Fig. 4).

### Phylogenetic analysis based on plastome data

**Fig. 5:**
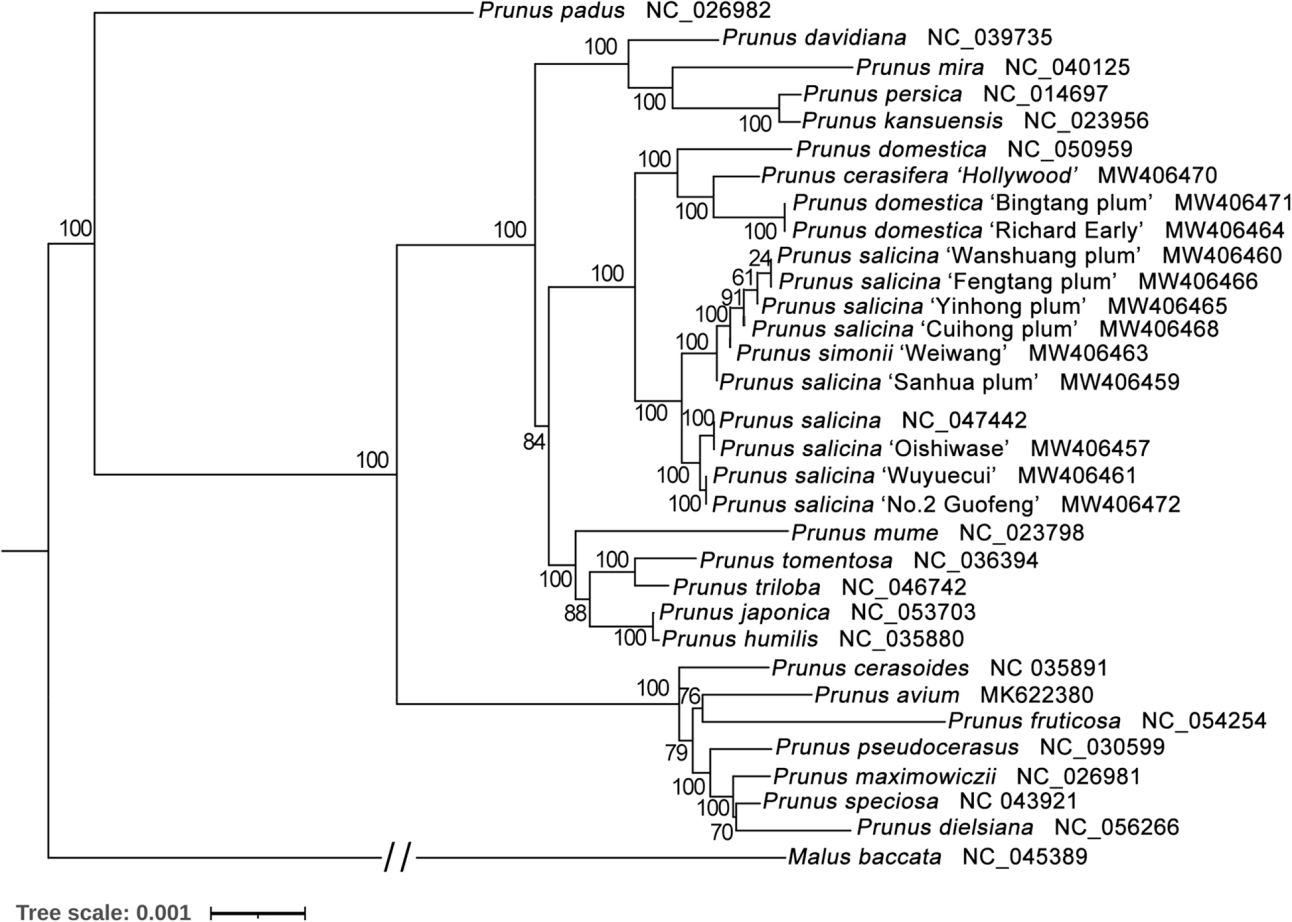
Phylogenetic relationships of species from *Prunus* (Rosaceae) inferred using Maximum likelihood (ML) method. The phylogenetic tree was constructed using the complete plastome sequences among the 32 plastomes. The number at the bottom of the scale, 0.001, means that the length of the branch represents the replacement frequency of bases at each site of the genome at 0.001. Bootstrap values were calculated from 1000 replicates

To examine the phylogenetic position of the twelve plum cultivars, we constructed maximum likelihood (ML) trees based on complete plastome sequences (Fig. 5) and 71 common protein-coding genes shared among 32 species from *Prunus* (Figure S12A), including the twelve sequenced in this study (Table S5). Two trees had a similar topological structure. However, the varieties in this study have the same structure between the two results. They are distributed in three clades. The first clade is formed by ‘Hollywood’, Richard Early’ and ‘Bingtang plum’ with *P. domestica*. Besides, ‘Oshiwase’, Wuyuecui’ and ‘No.2 Guofeng’ were clustered with *P. salicina*. The other six are grouped into another clade. It indicated that these varieties are a most likely hybrid between *P. domestica* and *P. salicina*. Most nodes of the phylogenetic tree have high bootstrap support, indicating the reliability of the phylogenetic analysis.

### Identification and validation of plastome-based markers

To distinguish the twelve cultivars, we selected six hypervariable regions manually based on plastome to develop six molecular markers named LZ01 to LZ06. The primers used for validation are shown in Table S6. All of PCR amplification results have single and bright band. The DNA fragments were extracted from each band and then sent for Sanger sequencing. The sequencing results were identical to the previous sequences.

**Fig. 6:**
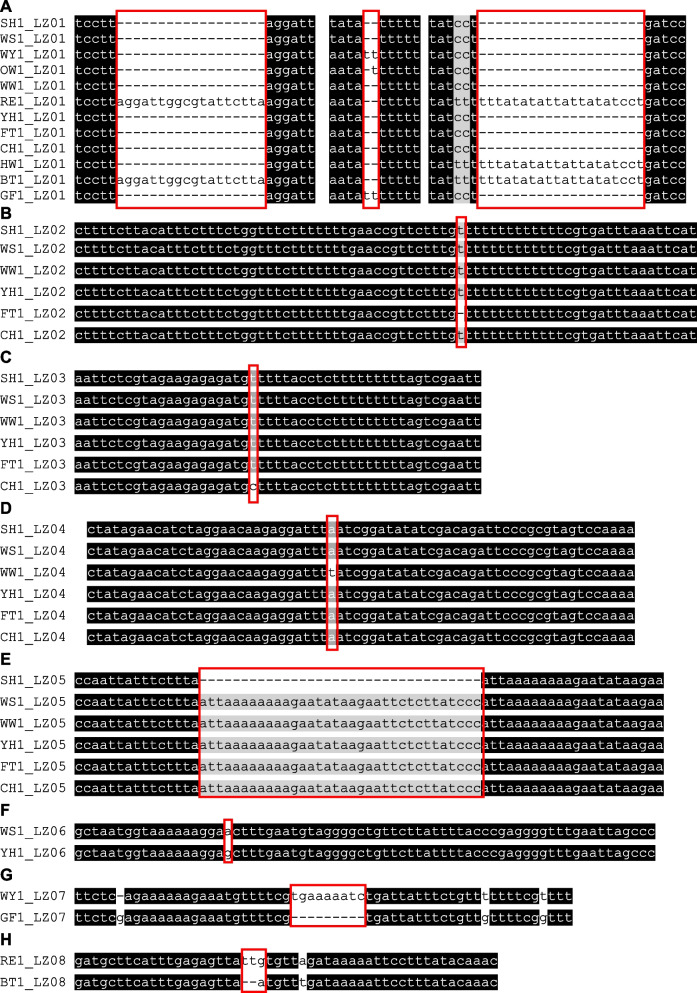
The alignment of the sequencing of the PCR products amplified using the primer LZ01 to LZ08. A-H represents the alignment using LZ01, LZ02, LZ03, LZ04, LZ05, LZ06, LZ07 and LZ08, respectively. The SNP and Indel regions are highlighted with red squares. The nucleotides identical across all plastomes are shaded in black, whereas those conserved in 60% of the sequences are shaded in gray. SH: *P. salicina* 'Sanhua plum'; WS: *P. salicina* 'Wanshuang plum'; WY: *P. salicina* 'Wuyuecui'; OW: *P. salicina* 'Oishiwase'; WW: *P. simonii* 'Weiwang'; RE: *P. domestica* 'Richard Early'; YH: *P. salicina* 'Yinhong plum'; FT: *P. salicina* 'Fengtang plum'; CH: *P. salicina* 'Cuihong plum'; HW: *P. cerasifera* 'Hollywood'; BT: *P. domestica* 'Bingtang plum'; GF: *P. salicina* 'No.2 Guofeng'. Arabic numerals represent different individuals

Marker LZ01 can divide the twelve varieties into five groups, named Group1 to Group5 (Group1: ‘Sanhua plum’ (SH), ‘Wanshuang plum’ (WS), ‘Weiwang’ (WW), ‘Yinhong plum’ (YH), ‘Fengtang plum’ (FT) and ‘Cuihong plum’ (CH); Group2: ‘Oishiwase’ (OW); Group3: ‘Wuyuecui’(WY) and ‘No.2 Guofeng’ (GF); Group4: ‘Hollywood’ (HW); Group5: ‘Richard Early’ (RE) and ‘Bingtang plum’ (BT)). And two varieties: ‘Oishiwase’ (OW) and ‘Hollywood’ (HW) can be distinguished with three Indels (Fig. 6A). For the Group1, Marker LZ02, LZ03,LZ04, LZ05 and LZ06 can distinguish ‘Fengtang plum’ (FT) (Fig. 6B), ‘Cuihong plum’ (CH) (Fig. 6C), ‘Weiwang’ (WW) (Fig. 6D), ‘Sanhua plum’ (SH) (Fig. 6E) and ‘Yinhong plum’ (YH) (Fig. 6F), respectively. Unfortunately, for the Group3 and Group5, their plastome sequences are the same as another. As a result, we further developed markers for these cultivars based on nuclear genome.

### Identification and validation of nuclear genome-based markers

To identify the remaining four cultivars, we extracted nuclear genes from sequence data among the Angiosperms-mega 353 gene set [24]. Among these genes, 342, 295, 331, 339 genes had extracted for ‘Wuyuecui’, ‘Richard Early’, ‘Bingtang plum’ and ‘No.2 Guofeng’, respectively. Among these coding sequences, 254 genes were shared among the four cultivars. These common genes were used to construct a phylogenetic tree using the same method as that for the complete plastome sequences. The relationships in both the nuclear and plastome trees were consistent (Figure S12B). We selected two hypervariable regions from two genes (AT2G45770 and AT4G02790) to develop two molecular markers named LZ07 and LZ08. The same method for PCR amplification and Sanger sequencing as the above. All of the PCR amplification results have single and bright band. The sequencing results were identical to the previous sequences (Fig. 6G, H).

To verify the reliability of the markers, we also collected plant materials for three individuals from another region (Table 2). All individuals used the same method for DNA extraction, PCR amplification and Sanger sequencing as the above. These markers can discriminate all six individuals from two regions (Figures S13, S14, S15, S16, S17, S18, S19, S20, S21, S22, S23, S24, S25, S26, S27, S28). The identification scheme is shown in the Fig. 7.

**Table 2:** Summary information of the plant samples

Sample	Collection places1	Geospatial coordinate1	Collection places2	Geospatial coordinate2
*P. salicina* 'Sanhua plum'	Qianpai, Xinyi, Guangdong	N22.36357/E111.26705	Qianpai, Xinyi, Guangdong	N22.36325/E111.11401
*P. salicina* 'Wanshuang plum'	Fenshui, Wanzhou, Chongqing	N30.71991/E108.08268	Xikou, Wanzhou, Chongqing	N30.61144/E108.35160
*P. salicina* 'Wuyuecui'	Shuangshi, Rong, Sichuan	N29.37621/E104.47442	Chishui, Luzhou, Sichuan	N27.73962/E105.57788
*P. salicina* 'Oishiwase'	Xiongyue, Yingkou, Liaoning	N40.19776/E122.18046	Xiannvshan, Wulong, Chongqing	N29.39880/E107.71477
*P. simonii* 'Weiwang'	Sanjiao, Qijiang, Chongqing	N28.99009/E106.75957	Baishiyi, Jiulongpo, Chongqing	N29.45479/E106.35890
*P. domestica* 'Richard Early'	Xiongyue, Yingkou, Liaoning	N40.19776/E122.20143	Sanjiao, Qijiang, Chongqing	N29.07032/E106.75494
*P. salicina* 'Yinhong plum'	Yibin, Sichuan	N28.65130/E104.76381	Yibin, Sichuan	N28.73574/E104.45183
*P. salicina* 'Fengtang plum'	Liuma, Zhenning, Guizhou	N25.70337/E105.81562	Daxing, Bishan, Chongqing	N29.52927/E106.16698
*P. salicina* 'Cuihong plum'	Leshan, Sichuan	N29.40460/E103.63412	Baishiyi, Jiulongpo, Chongqing	N29.45544/E106.35833
*P. cerasifera* 'Hollywood'	Xiongyue, Yingkou, Liaoning	N40.18739/E122.18315	Baishiyi, Jiulongpo, Chongqing	N29.45487/E106.35919
*P. domestica* 'Bingtang plum'	Xiongyue, Yingkou, Liaoning	N40.18623/E122.19721	Baishiyi, Jiulongpo, Chongqing	N29.45470/E106.35884
*P. salicina* 'No.2 Guofeng'	Xiongyue, Yingkou, Liaoning	N40.19776/E122.22141	Baishiyi, Jiulongpo, Chongqing	N29.45440/E106.35882

**Fig. 7:**
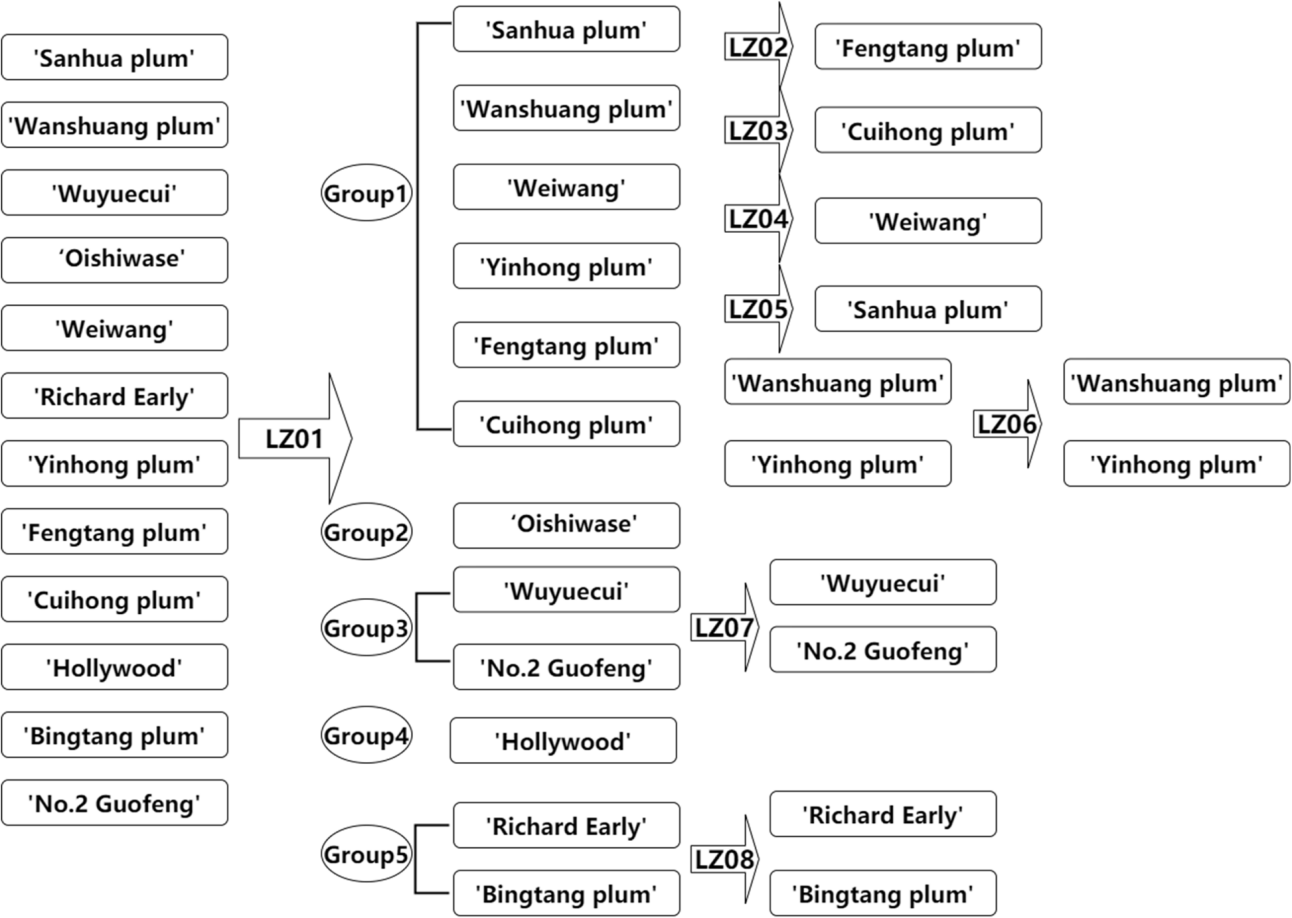
The identification scheme for this study. The boxes represent varieties names. The arrows represent the primers names

## Discussion

We first reported the plastome sequences of twelve plum cultivars. Our assembly results showed that the length of twelve plastomes ranged from 157,863 bp to 157,952 bp. This result is similar to most Rosaceae plant plastomes [25, 26]. In our study, the longest and shortest plastome sequences were 158,955 bp (*Prunus padus*) and 157,395 bp (*Prunus domeatica*), respectively. This suggests that the plastomes of *Prunus* may be evolutionarily different. The plastome of angiosperms evolves faster, with inversions and gene loss occurring during evolution [27]. Among our study, the longest genome sequence was found in *P. cerasifera* ‘Hollywood’ (157,952 bp) and the shortest was that of *P. salicina* ‘Sanhua plum’ (157,863 bp). They have a similar structure to most angiosperms, and we didn’t find gene gain/loss, thus, the plastomes were still relatively conserved. In terms of gene composition, these species encoded 131 genes, including 86 protein-coding genes, 37 tRNA genes, and 8 rRNA genes. The plastomes among *Prunus* varieties were similar in intron and GC contents, but the GC contents in LSC and SSC regions were significantly lower than that in the IR region. These results are similar to those reported previously [28–31].

In this study, we examined the SSRs and repetitive sequences of twelve plastomes. 593 of the 629 SSRs were mononucleotide repeats, accounting for the majority of all SSRs (94.28%). These mononucleotide repeats were mainly A/T repeats, which had a significant effect on the overall G/C content of the genome [32, 33]. They are often used as molecular markers due to the length of polymorphism in different species. Plastomes are rich in SSR loci and have been recommended for species identification [34, 35]. Besides, we also detected three kinds of repeated sequences in twelve plastomes. Among them, *P. salicina* 'Oishiwase' had the most repeats. Genomic recombination and sequence variation were mainly caused by slip-strand mismatches and inappropriate recombination of repetitive sequences [35, 36]. These repeats are the genetic markers that are the basis of population and phylogenetic studies and are widely used because of their high polymorphism rates [37–40].

Typically, the IR region is the most conserved region of the chloroplast genome [41]. The expansion and contraction of IR, LSC and SSC regions are common during the evolutionary process and are the main reasons for the differences in plastomes length [42, 43]. For example, *Cicer arietinum* and *Pisum sativum* were found to lack a copy of the IR region [44, 45], *Cephalotaxus oliveri* was no IR region [36], and gene loss events were identified in the plastome of *Astragalus membranaceus* [27]. *Pelargonium hortorum* and *Pinus thunbergii* plastome showed expansion/ contraction events in the IR region leading to the length of the plastome being unusual [46, 47]. A comparative map of chloroplast genome boundary regions was obtained by analyzing the boundary genes of the IR, LSC and SSC regions of the plastome [48, 49]. In our study, all species have two copies of the IR regions. Gene *ycf*1 is located in the SSC and IRa regions, the length in IRa is between 391 bp to 1,051 bp. These overlapping segments resulted in a pseudogene fragment of *ycf*1 at the IRb/SSC boundary except *P. padus* and *P. mume*. Especially, pseudogene *ycf*1 of *P. persica* is all in the IRb region. However, except for the *P. avium* and *rps*19 genes that are located in the boundary of LSC/IRb, the length in the IR region is between 39 bp (*P. padus*) and 197 bp (*P. mume*). Thus, the length of the IR region of *P. padus* is shorter than others.

The phylogenetic relationships of Rosaceae have long been problematic because of frequent hybridization, asexual reproduction, presumed rapid radiation, and historical diversification [50]. In this study, we obtained identical phylogenetic relationships for the twelve cultivars using complete plastomes and common protein-coding genes. Three cultivars: ‘Hollywood’, ‘Bingtang plum’ and ‘Richard Early’ are close to the European plum (*P. domestica*). The others are close to the Chinese plum (*P. salicina*). This also can confirm their breeding background [7, 11, 51–55].

Currently, there are many studies on molecular markers for the *Prunus*. But there are few studies on the identification of *Prunus* based on the plastome, which is extremely conserved and has many variant loci and is ideal data for molecular marker development. In this study, we identified 12 plum cultivars based on the plastomes and used nuclear genes to identify some of the plants that could not be distinguished from the chloroplast genome. In this experiment, the plastomes of the twelve plum cultivars differed very little, and the highly variable regions screened by the K2p model could not achieve the purpose of distinguishing the individual resources by sequence comparison; therefore, we manually screened the regions with large variation and validated eight molecular markers that could identify them.

Over all, we first developed markers to identify the twelve plum cultivars. DNA markers can comprehensively compare genetic material between populations and individuals, and improve the accuracy and reliability of plant classification. The genetic distance is related to the sequence divergence [56]. In this study, the plastomes of twelve plum cultivars differ slightly. According to the hypervariable region using the K2p module, the most variable regions can’t distinguish each variety. Thus, we selected the different segments manually and verified eight markers that could separate each of them.

## Conclusions

The complete plastomes of twelve plum cultivars are reported for the first time in this study. These twelve cultivars are closely related to *P. salicina* and *P. domestica*. In addition, we successfully developed a scheme using eight molecular makers in plastome and nuclear genome. Our results provide a wider perspective on the basis of the plastomes of *Prunus* to the molecular identification and phylogenetic construction.

## Methods

### Plant material, DNA extraction and Sequencing

The fresh leaves of twelve plants were collected from Chongqing, Guangdong, Sichuan, Liaoning, Guizhou. All the samples were saved deposited at the Herbarium of Southwest University, Chongqing, China. The detailed information for the plant samples is shown in Table 1. The total genomic DNA was extracted by using the CTAB method [57]. The DNA library was constructed using the Agilent 2100 and sequenced using the Illumina NovaSeq 6000 sequencing platform. Sequencing produced a total of 5.04 – 6.26 G raw data per sample. Clean data were obtained by removing low-quality sequences: sequences with a quality value of Q <  = 5 accounted for more than 50% of the total base, sequences with more than 10% bases being “N”, and sequences having an adapter.

### Genome assembly and annotation

The chloroplast genome was assembled from the clean data by GetOrganelle (v. 1.6.4) [58]. The correctness of the assembly was confirmed by using Bowtie2 (v2.0.1) [59] to manually edit and map all raw reads to the assembled genome sequence under the default settings. The annotation of the plastome was conducted initially using CpGAVAS2 [60]. Geseq was then used to confirm the annotation results [61]. Furthermore, the annotations with problems were manually edited by using Apollo [62]. The genome sequence and annotations have been deposited in the GenBank with accession numbers MW406457, MW406459, MW406460, MW406461, MW406463, MW406464, MW406465, MW406466, MW406468, MW406470, MW406471, MW406472.

### Repeats and SSR analysis

The GC content was conducted by using the cusp program provided by EMBOSS (v6.3.1) [63]. The simple sequence repeats (SSRs) were identified using the Online website MISA (https://webblast.ipk-gatersleben.de/misa/), including mono-, di-, tri-, tetra-, penta-, and hexanucleotides with the minimum numbers were 10, 5, 4, 3, 3, and 3, respectively [64]. Additionally, REPuter (https://bibiserv.cebitec.uni-bielefeld.de/reputer/) was used to calculate palindromic repeats, forward repeats, reverse repeats, and complement repeats with the settings: Hamming Distance was three, and Minimal Repeat Size was 30 bp [65].

### Genome comparison

The multiple sequences were aligned using ClustalW2 [66]. The intergenic regions were extracted with extractseq from EMOSS [63]. The distances of intergenic spacers were conducted using the program distmat from EMBOSS [63]. IRscope (https://irscope.shinyapps.io/irapp/) was used for visualizing the IR boundaries in these plastomes [67].

### Phylogenetic analysis

Except for the twelve sequences in this study, the plastome sequences of 19 species belonging to the genus *Prunus* were downloaded from GenBank (NCBI, https://www.ncbi.nlm.nih.gov/). *Malus baccata* (Rosaceae) was used as an outgroup. The details are shown in Table S1. The complete plastome sequences were aligned by using MAFFT (https://mafft.cbrc.jp/alignment/server/) [68]. These aligned sequences were used to construct the phylogenetic trees by using the Maximum Likelihood (ML) method implemented in RaxML (v8.2.4) [69]. The parameters were “raxmlHPC-PTHREADS-SSE3 -f a -N 1000 -m GTRGAMMA—× 551,314,260 -p 551,314,260”. The bootstrap analysis was performed with 1,000 replications. As for the common genes, we extracted 71 protein-coding genes from 32 species. The method to construct the tree is the same with the above.

### Identification of nuclear markers for phylogenetic analysis

To distinguish the four varieties whose plastome sequences are pairwise consistent, we used the pipeline HybPiper (v1.2) (https://github.com/mossmatters/HybPiper) to identify nuclear markers with the default settings to process our cleaned data [70]. The HybPiper package contains an internal reference set of 353 genes [24]. This Angiosperms-mega 353 gene set can capture loci in our sequence reads. The identified contigs matching probe can be extract using the command line “./reads_first.py -b mega353.fasta -r sample_R1.fastq sample_R2.fastq –prefix sample_result –bwa”. And we selected the common genes among the four varieties to construct the phylogenetic tree using RaxML with 1000 bootstrap replicates. *Oryza sativa* is the outgroup.

### Identification and validation of molecular markers for discrimination

We selected different segments manually to develop molecular markers. Primers were designed using the IDT website (https://sg.idtdna.com/pages/tools/primerquest?returnurl=%2FPrimerquest%2FHome%2FIndex). We collected three individuals from each variety. DNA samples were extracted and then subjected to PCR amplification on a Pro Flex PCR system (Applied Biosystems, Waltham, MA, USA). PCR amplifications were performed in a final volume of 25μL with 2 μL template DNA, 1 μl of forwarding primer, 1 μl of reverse primer, 12.5 μL 2 × Taq PCR Master Mix and 8.5 μL ddH_2_0. PCR experiments were conducted under the following conditions: pre-denaturation at 94 ˚C for 5 min, 30 cycles of amplification at 94 ˚C for 30 s, 58 ˚C for 30 s, and 72 ˚C for 60 s, followed by a final extension at 72 ˚C for 5 min. The PCR products were evaluated with 1% agarose gelelectrophoresis. Only single bands were subjected to Sanger sequencing.

## Supplementary Information

**Additional file 1:** **TableS1. **Summary of sequencing data quality. **Table S2. **Gene composition in the plastomes of twelve plum cultivars*. ***Table S3**. Length of introns and exons inthe plastomes of twelve plumcultivars. **Table S4.** Statistics on simple sequencerepeats (SSRs) in the twelve plastomes. **TableS5.** The list of accession numbers of the plastome sequences used in thephylogenetic analyses of the *Prunus*. **FigureS1.** Genome map of *P. salicina* ‘Wanshuang plum’ plastome. **Figure S2.** Genome map of *P. salicina* ‘Wuyuecui’ plastome. **Figure S3.** Genome map of *P. salicina* ‘Oishiwase’ plastome. **Figure S4.** Genome map of *P. simonii* 'Weiwang' plastome. **Figure S5.** Genome map of *P. domestica* 'Richard Early' plastome. **Figure S6.** Genome map of *P. salicina* 'Yinhong plum' plastome. **Figure S7.** Genome map of *P. salicina* ' Fengtang plum' plastome. **Figure S8.** Genome map of *P. salicina* ' Cuihong plum' plastome. **Figure S9.** Genome map of* P. cerasifera* 'Hollywood' plastome. **Figure S10.** Genome map of *P. domestica* 'Bingtang plum' plastome. **Figure S11.** Genome map of *P. salicina* 'No.2 Guofeng' plastome. **Figure S12.** Phylogenetic relationshipsof species from *Prunus* (Rosaceae)inferred using Maximum likelihood (ML) method.** Figure S13.** The gel electrophoresis results of the amplificationof DNA barcodes using designed primer LZ01. **Figure S14.** The gel electrophoresis results of the amplification ofDNA barcodes using designed primer LZ02. **FigureS15.** The gel electrophoresis results of the amplification of DNA barcodesusing designed primer LZ03. **Figure S16.**The gel electrophoresis results of the amplification of DNA barcodes usingdesigned primer LZ04. **Figure S17.**The gel electrophoresis results of the amplification of DNA barcodes usingdesigned primer LZ05. **Figure S18.**The gel electrophoresis results of the amplification of DNA barcodes usingdesigned primer LZ06. **Figure S19.**The gel electrophoresis results of the amplification of DNA barcodes usingdesigned primer LZ07. **Figure S20.**The gel electrophoresis results of the amplification of DNA barcodes usingdesigned primer LZ08. **Figure S21.**The alignment of amplicons produced by designed LZ01 primer. **Figure S22.** The alignment of ampliconsproduced by designed LZ02 primer. **FigureS23.** The alignment of amplicons produced by designed LZ03 primer. **Figure S24.** The alignment of ampliconsproduced by designed LZ04 primer. **FigureS25.** The alignment of amplicons produced by designed LZ05 primer. **Figure S26.** The alignment of ampliconsproduced by designed LZ06 primer. **FigureS27. **The alignment of amplicons produced by designed LZ07 primer. **Figure S28.** The alignment of ampliconsproduced by designed LZ08 primer.

## Data Availability

The annotated chloroplast genome sequences of twelve plum cultivars were deposited in GeneBank (https://www.ncbi.nlm.nih.gov/) with Accession number: MW406457 (*P. salicina* 'Oishiwase'), MW406459 (*P. salicina* ‘Sanhua plum’), MW406460 (*P. salicina* ‘Wanshuang plum’), MW406461 (*P. salicina* ‘Wuyuecui’), MW406463 (*P. simonii* 'Weiwang'), MW406464 (*P. domestica* 'Richard Early'), MW406465 (*P. salicina* 'Yinhong plum'), MW406466 (*P. salicina* 'Fengtang plum'), MW406468 (*P. salicina* 'Cuihong plum'), MW406470 (*P. cerasifera* 'Hollywood'), MW406471 (*P. domestica* 'Bingtang plum') and MW406472 (*P. salicina* 'No.2 Guofeng'). Raw sequence data for this study also can be found in GenBank. The associated BioProject, SRA, Bio-Sample numbers are PRJNA795302, SRR17478141, SAMN24706757 (*P. salicina* 'Oishiwase'); PRJNA795262, SRR17477716, SAMN24699028 (*P. salicina* ‘Sanhua plum’); PRJNA795282, SRR17477883, SAMN24704169 (*P. salicina* ‘Wanshuang plum’); PRJNA795299, SRR17478140, SAMN24705967(*P. salicina* ‘Wuyuecui’); PRJNA719267, SRR14133449, SAMN18593728 (*P. simonii* 'Weiwang'); PRJNA795321, SRR17478255, SAMN24707166 (*P. domestica* 'Richard Early'); PRJNA795322, SRR17478256, SAMN24707171 (*P. salicina* 'Yinhong plum'); PRJNA795338, SRR17480298, SAMN24708705 (*P. salicina* 'Fengtang plum'); PRJNA795340, SRR17480297, SAMN24709469 (*P. salicina* 'Cuihong plum'); PRJNA795572, SRR17498860, SAMN24731932 (*P. cerasifera* 'Hollywood'); PRJNA795571, SRR17498195, SAMN24731931 (*P. domestica* 'Bingtang plum'); PRJNA795581, SRR17498228, SAMN24734651 (*P. salicina* 'No.2 Guofeng'). All the samples are saved at the Herbarium of Southwest University, Chongqing, China. All other data and material generated in this manuscript are available from the corresponding author upon reasonable request.

## Abbreviations

SSRs: Simple sequence repeats
IR: Inverted repeat
LSC: Long Single Copy
SSC: Short Single Copy
IGS: Intergenic spacer
tRNA: Transfer RNA
rRNA: Ribosomal RNA
