# Supplementary material for: Phylogenomic analysis and development of molecular markers for the determination of twelve plum cultivars (Prunus, Rosaceae)
Source: BMC Genomics. 2022 Nov 8;23:745. doi: 10.1186/s12864-022-08965-z (PMC9644608; doi:10.1186/s12864-022-08965-z)
Supplement: Supplementary file 1 — Additional file 1: TableS1. Summary of sequencing data quality. Table S2. Gene composition in the plastomes of twelve plum cultivars. Table S3. Length of introns and exons inthe plastomes of twelve plumcultivars. Table S4. Statistics on simple sequencerepeats (SSRs) in the twelve plastomes. TableS5. The list of accession numbers of the plastome sequences used in thephylogenetic analyses of the Prunus. FigureS1. Genome map of P. salicina ‘Wanshuang plum’ plastome. Figure S2. Genome map of P. salicina ‘Wuyuecui’ plastome. Figure S3. Genome map of P. salicina ‘Oishiwase’ plastome. Figure S4. Genome map of P. simonii 'Weiwang' plastome. Figure S5. Genome map of P. domestica 'Richard Early' plastome. Figure S6. Genome map of P. salicina 'Yinhong plum' plastome. Figure S7. Genome map of P. salicina ' Fengtang plum' plastome. Figure S8. Genome map of P. salicina ' Cuihong plum' plastome. Figure S9. Genome map of P. cerasifera 'Hollywood' plastome. Figure S10. Genome map of P. domestica 'Bingtang plum' plastome. Figure S11. Genome map of P. salicina 'No.2 Guofeng' plastome. Figure S12. Phylogenetic relationshipsof species from Prunus (Rosaceae)inferred using Maximum likelihood (ML) method. Figure S13. The gel electrophoresis results of the amplificationof DNA barcodes using designed primer LZ01. Figure S14. The gel electrophoresis results of the amplification ofDNA barcodes using designed primer LZ02. FigureS15. The gel electrophoresis results of the amplification of DNA barcodesusing designed primer LZ03. Figure S16.The gel electrophoresis results of the amplification of DNA barcodes usingdesigned primer LZ04. Figure S17.The gel electrophoresis results of the amplification of DNA barcodes usingdesigned primer LZ05. Figure S18.The gel electrophoresis results of the amplification of DNA barcodes usingdesigned primer LZ06. Figure S19.The gel electrophoresis results of the amplification of DNA barcodes usingdesigned primer LZ07. Figure S20.The gel electrophoresis results of th [file 12864_2022_8965_MOESM1_ESM.pdf]

Title

Phylogenomic analysis and development of molecular markers for the determination of twelve plum cultivars (*Prunus*, Rosaceae)

Authors

Yicen Xu[1], Bo Fang[2], Jingling Li[1], Yuanwei Wang[3], Jingting Liu[4], Chang Liu[4*] and Jie Yu[1*]

1 College of Horticulture and Landscape Architecture, Southwest University, Chongqing, 400715, China.
2 Chongqing Academy of Agricultural Sciences, Chongqing, 401329, China.
3 Improved Seed Farm in Liangping District, Chongqing, 405299, China.
4 Institute of Medicinal Plant Development, Chinese Academy of Medical Sciences, Peking Union Medical College, Beijing 100193, China.

* Correspondence:
cliu6688@yahoo.com (C.L); Tel.:+86-10-57833111, Fax: +86-10- 62899715
yujie1982@swu.edu.cn (J.Y); Tel.: +86-23-68250755

Email Address:
YCX: xuyic0802@163.com
BF: Fbo211@hotmail.com
JLL: Lijingling1997@163.com
YWW: 451243627@qq.com
JTL: liujingtingy@163.com
CL: cliu6688@yahoo.com
JY: yujie1982@swu.edu.cn

Supplementary Table and Figure Legend

Table S1. Summary of sequencing data quality.

Table S2. Gene composition in the plastomes of twelve plum cultivars.

Table S3. Length of introns and exons in the plastomes of twelve plum cultivars.

Table S4. Statistics on simple sequence repeats (SSRs) in the twelve plastomes.

Table S5. The list of accession numbers of the plastome sequences used in the phylogenetic analyses of the *Prunus*.

Figure S1. Genome map of *P. salicina* 'Wanshuang plum' plastome.

Figure S2. Genome map of *P. salicina* 'Wuyuecui' plastome.

Figure S3. Genome map of *P. salicina* 'Oishiwase' plastome.

Figure S4. Genome map of *P. simonii* 'Weiwang' plastome.

Figure S5. Genome map of *P. domestica* 'Richard Early' plastome.

F**igure S6.** Genome map of *P. salicina* 'Yinhong plum' plastome.

Figure S7. Genome map of *P. salicina* ' Fengtang plum' plastome.

Figure S8. Genome map of *P. salicina* ' Cuihong plum' plastome.

Figure S9. Genome map of *P. cerasifera* 'Hollywood' plastome.

Figure S10. Genome map of *P. domestica* 'Bingtang plum' plastome.

Figure S11. Genome map of *P. salicina* 'No.2 Guofeng' plastome.

Figure S12. Phylogenetic relationships of species from *Prunus* (Rosaceae) inferred using Maximum likelihood (ML) method.

Figure S13. The gel electrophoresis results of the amplification of DNA barcodes using designed primer LZ01.

Figure S14. The gel electrophoresis results of the amplification of DNA barcodes using designed primer LZ02.

Figure S15. The gel electrophoresis results of the amplification of DNA barcodes using designed primer LZ03.

Figure S16. The gel electrophoresis results of the amplification of DNA barcodes using designed primer LZ04.

Figure S17. The gel electrophoresis results of the amplification of DNA barcodes using designed primer LZ05.

Figure S18. The gel electrophoresis results of the amplification of DNA barcodes using designed primer LZ06.

Figure S19. The gel electrophoresis results of the amplification of DNA barcodes using designed primer LZ07.

Figure S20. The gel electrophoresis results of the amplification of DNA barcodes using designed primer LZ08.

Figure S21. The alignment of amplicons produced by designed LZ01 primer.

Figure S22. The alignment of amplicons produced by designed LZ02 primer.

Figure S23. The alignment of amplicons produced by designed LZ03 primer.

Figure S24. The alignment of amplicons produced by designed LZ04 primer.

Figure S25. The alignment of amplicons produced by designed LZ05 primer.

Figure S26. The alignment of amplicons produced by designed LZ06 primer.

Figure S27. The alignment of amplicons produced by designed LZ07 primer.

Figure S28. The alignment of amplicons produced by designed LZ08 primer.

Table S1. Summary of sequencing data quality.

Varieties	*P. salicina* 'Sanhua plum'	*P. salicina* 'Wanshuang plum'	*P. salicina* 'Wuyuecui'	*P. salicina* 'Oishiwase'	*P. simonii* 'Weiwang'	*P. domestica* 'Richard Early'
Raw Reads	19,815,279	17,536,403	19,624,728	20,448,186	19,667,246	17,711,776
Clean Reads	19,715,778	17,445,941	19,523,760	20,310,868	19,555,048	17,563,770
Raw Base (G)	5.94	5.26	5.89	6.13	5.9	5.31
Clean Base (G)	5.91	5.23	5.86	6.09	5.87	5.27
Effective Rate (%)	99.50	99.48	99.49	99.33	99.43	99.16
Error Rate (%)	0.03	0.03	0.03	0.03	0.03	0.03
Q20 (%)	97.09	97.10	96.95	96.97	97.25	97.16
Q30 (%)	92.02	92.10	91.75	91.75	92.37	92.19
GC Content (%)	38.51	38.97	38.94	39.08	39.42	38.70
Varieties	*P. salicina* 'Yinhong plum'	*P. salicina* 'Fengtang plum'	*P. salicina* 'Cuihong plum'	*P. cerasifera* 'Hollywood'	*P. domestica* 'Bingtang plum'	*P. salicina* 'No.2 Guofeng'
Raw Reads	19,270,256	16,790,616	20,751,812	20,854,169	19,934,460	20,396,281
Clean Reads	19,187,146	16,709,174	20,651,324	20,713,829	19,765,663	20,291,493
Raw Base (G)	5.78	5.04	6.23	6.26	5.98	6.12
Clean Base (G)	5.76	5.01	6.2	6.21	5.93	6.09
Effective Rate (%)	99.57	99.51	99.52	99.33	99.15	99.49
Error Rate (%)	0.03	0.03	0.03	0.03	0.03	0.03
Q20 (%)	97.03	96.94	97.23	96.95	97.39	97.25
Q30 (%)	91.94	91.54	92.31	91.70	92.65	92.37
GC Content (%)	39.51	39.44	40.08	39.00	38.77	39.47

Table S2. Gene composition in the plastomes of twelve plum cultivars.

**Category of Genes**	**Group of Genes**	**Name of Genes**
Self-replication	Ribosomal RNA	*rrn*16S (x2), *rrn*23S (x2), *rrn*5S (x2), *rrn4.*5S (x2)
	Transfer RNA	*trn*A-UGC(x2), *trn*C-GCA, *trn*D-GUC, *trn*E-UUC, *trn*F-GAA, *trn*fM-CAU, *trn*G-UCC(x2), *trn*H-GUG, *trn*I-CAU (x2), *trn*I-GAU (x2), *trn*K-UUU, *trn*L-CAA (x2), *trn*L-UAA, *trn*L-UAG, *trn*M-CAU, *trn*N-GUU(x2), *trn*P-UGG, *trn*Q-UUG, *trn*R-ACG (x2), *trn*R-UCU, *trn*S-GCU(x2), *trn*S-UGA, *trn*T-GGU, *trn*T-UGU, *trn*V-GAC (x2), *trn*V-UAC, *trn*W-CCA, *trn*Y-GUA
	Large subunit of ribosome	*rpl*14, *rpl*16, *rpl*2 (x2), *rpl*20, *rpl*22, *rpl*23 (x2), *rpl*32, *rpl*33, *rpl*36
	DNA dependent RNA polymerase	*rpo*A, *rpo*B, *rpo*C1, *rpo*C2
	Small subunit of ribosome	*rps*11, *rps*12 (x2), *rps*14, *rps*15, *rps*16, *rps*18, *rps*19(x2), *rps*2, *rps*3, *rps*4, *rps*7(x2), *rps*8
Photosynthesis	Subunits of *ATP* synthase	*atp*A, *atp*B, *atp*E, *atp*F, *atp*H, *atp*I
	Subunits of photosystem II	*psb*A, *psb*B, *psb*C, *psb*D, *psb*E, *psb*F, *psb*I, *psb*J, *psb*K, *psb*L, *psb*M, *psb*N, *psb*T, *psbZ*, *ycf*3
	Subunits of NADH-dehydrogenase	*ndh*A, *ndh*B (x2), *ndh*C, *ndh*D, *ndh*E, *ndh*F, *ndh*G, *ndh*H, *ndh*I, *ndh*J, *ndh*K
	Subunits of cytochrome b/f complex	*pet*A, *pet*B, *pet*D, *pet*G, *pet*L, *pet*N
	Subunits of photosystem I	*psa*A, *psa*B, *psa*C, *psa*I, *psa*J
	Subunit of rubisco	*rbc*L
Other Genes	Subunit of Acetyl-CoA-carboxylase	*acc*D
	c-type cytochrom synthesis gene	*ccs*A
	Envelop membrane protein	*cem*A
	Protease	*clp*P
	Maturase	*mat*K
Unknown	Conserves open reading frames	*ycf*1 (x2), *ycf*2 (x2), *ycf*4

Table S3. Length of introns and exons in the plastomes of twelve plum cultivars.

Varieties	Gene	Strand	Start	End	ExonI	IntronI	ExonII	IntronII	ExonIII
P. salicina ‘Sanhua plum’	*trn*K-UUU	-	1799	4406	37	2536	35		
	*rps*16	-	5337	6470	40	864	230		
	*trn*G-UCC	+	9015	9800	23	714	49		
	*atp*F	-	12021	13318	145	743	410		
	*rpo*C1	-	21405	24210	434	754	1618		
	*ycf*3	-	44304	46296	124	723	230	763	153
	*trn*L-UAA	+	49300	49900	37	514	50		
	*trn*V-UAC	-	53406	54071	39	590	37		
	*clp*P	-	71902	73947	71	811	291	647	226
	*pet*B	+	76864	78266	6	755	642		
	*pet*D	+	78454	79680	8	744	475		
	*rpl*16	-	83190	84608	9	1011	399		
	*rpl*2	-	86387	87888	385	683	434		
	*ndh*B	-	96970	99182	775	680	758		
	*trn*I-GAU	+	104604	105623	42	943	35		
	*trn*A-UGC	+	105688	106567	38	807	35		
	*ndh*A	-	122709	124949	553	1149	539		
	*trn*A-UGC	-	137427	138306	38	807	35		
	*trn*I-GAU	-	138371	139390	42	943	35		
	*ndh*B	+	144812	147024	775	680	758		
	*rpl*2	+	156106	157607	385	683	434		
P. salicina ‘Wanshuang plum’	*trn*K-UUU	-	1824	4433	37	2538	35		
	*rps*16	-	5364	6497	40	864	230		
	*trn*G-UCC	+	9043	9828	23	714	49		
	*atp*F	-	12049	13346	145	743	410		
	*rpo*C1	-	21435	24240	434	754	1618		
	*ycf*3	-	44334	46326	124	723	230	763	153
	*trn*L-UAA	+	49330	49930	37	514	50		
	*trn*V-UAC	-	53436	54101	39	590	37		
	*clp*P	-	71963	74007	71	810	291	647	226
	*pet*B	+	76924	78325	6	754	642		
	*pet*D	+	78513	79739	8	744	475		
	*rpl*16	-	83250	84668	9	1011	399		
	*rpl*2	-	86446	87947	385	683	434		
	*ndh*B	-	97029	99241	775	680	758		
	*trn*I-GAU	+	104663	105682	42	943	35		
	*trn*A-UGC	+	105747	106626	38	807	35		
	*ndh*A	-	122769	125009	553	1149	539		
	*trn*A-UGC	-	137490	138369	38	807	35		
	*trn*I-GAU	-	138434	139453	42	943	35		

	*ndh*B	+	144875	147087	775	680	758		
	*rpl*2	+	156169	157670	385	683	434		
P. salicina ‘Wuyuecui’	*trn*K-UUU	-	1724	4332	37	2537	35		
	*rps*16	-	5273	6408	40	866	230		
	*trn*G-UCC	+	8917	9708	23	720	49		
	*atp*F	-	11934	13232	145	744	410		
	*rpo*C1	-	21327	24132	434	754	1618		
	*ycf*3	-	44189	46182	124	723	230	764	153
	*trn*L-UAA	+	49180	49780	37	514	50		
	*trn*V-UAC	-	53243	53908	39	590	37		
	*clp*P	-	71890	73924	71	806	291	641	226
	*pet*B	+	76841	78242	6	754	642		
	*pet*D	+	78430	79656	8	744	475		
	*rpl*16	-	83168	84586	9	1011	399		
	*rpl*2	-	86367	87868	385	683	434		
	*ndh*B	-	96983	99195	775	680	758		
	*trn*I-GAU	+	104617	105636	42	943	35		
	*trn*A-UGC	+	105701	106580	38	807	35		
	*ndh*A	-	122718	124958	553	1149	539		
	*trn*A-UGC	-	137435	138314	38	807	35		
	*trn*I-GAU	-	138379	139398	42	943	35		
	*ndh*B	+	144820	147032	775	680	758		
	*rpl*2	+	156147	157648	385	683	434		
P. salicina ‘Oishiwase’	*trn*K-UUU	-	1721	4328	37	2536	35		
	*rps*16	-	5270	6399	40	860	230		
	*trn*G-UCC	+	8907	9698	23	720	49		
	*atp*F	-	11924	13223	145	745	410		
	rpoC1	-	21317	24122	434	754	1618		
	*ycf*3	-	44148	46132	124	715	230	763	153
	*trn*L-UAA	+	49153	49753	37	514	50		
	*trn*V-UAC	-	53241	53906	39	590	37		
	*clp*P	-	71883	73930	71	807	291	653	226
	*pet*B	+	76853	78253	6	753	642		
	*pet*D	+	78441	79667	8	744	475		
	*rpl*16	-	83181	84600	9	1012	399		
	*rpl*2	-	86381	87882	385	683	434		
	*ndh*B	-	96998	99210	775	680	758		
	*trn*I-GAU	+	104626	105645	42	943	35		
	*trn*A-UGC	+	105710	106589	38	807	35		
	*ndh*A	-	122734	124974	553	1149	539		
	*trn*A-UGC	-	137451	138330	38	807	35		
	*trn*I-GAU	-	138395	139414	42	943	35		
	*ndh*B	+	144830	147042	775	680	758		

	*rpl*2	+	156158	157659	385	683	434		
P. simonii ‘Weiwang’	*trn*K-UUU	-	1824	4433	37	2538	35		
	*rps*16	-	5364	6497	40	864	230		
	*trn*G-UCC	+	9043	9828	23	714	49		
	*atp*F	-	12049	13346	145	743	410		
	*rpo*C1	-	21433	24238	434	754	1618		
	*ycf*3	-	44332	46324	124	723	230	763	153
	*trn*L-UAA	+	49328	49928	37	514	50		
	*trn*V-UAC	-	53434	54099	39	590	37		
	*clp*P	-	71960	74005	71	811	291	647	226
	*pet*B	+	76922	78324	6	755	642		
	*pet*D	+	78512	79738	8	744	475		
	*rpl*16	-	83248	84666	9	1011	399		
	*rpl*2	-	86444	87945	385	683	434		
	*ndh*B	-	97027	99239	775	680	758		
	*trn*I-GAU	+	104661	105680	42	943	35		
	*trn*A-UGC	+	105745	106624	38	807	35		
	*ndh*A	-	122767	125007	553	1149	539		
	*trn*A-UGC	-	137488	138367	38	807	35		
	*trn*I-GAU	-	138432	139451	42	943	35		
	*ndh*B	+	144873	147085	775	680	758		
	*rpl*2	+	156167	157668	385	683	434		
P. domestica ‘Richard Early’	*trn*K-UUU	-	1713	4320	37	2536	35		
	*rps*16	-	5244	6381	40	868	230		
	*trn*G-UCC	+	8942	9730	23	717	49		
	*atp*F	-	11961	13258	145	743	410		
	*rpo*C1	-	21363	24168	434	754	1618		
	*ycf*3	-	44298	46281	124	714	230	763	153
	*trn*L-UAA	+	49250	49850	37	514	50		
	*trn*V-UAC	-	53292	53957	39	590	37		
	*clp*P	-	71953	74002	71	806	291	656	226
	*pet*B	+	76926	78327	6	754	642		
	*pet*D	+	78515	79741	8	744	475		
	*rpl*16	-	83249	84677	9	1021	399		
	*rpl*2	-	86458	87959	385	683	434		
	*ndh*B	-	97103	99315	775	680	758		
	*trn*I-GAU	+	104731	105750	42	943	35		
	*trn*A-UGC	+	105815	106694	38	807	35		
	*ndh*A	-	122756	124996	553	1149	539		
	*trn*A-UGC	-	137455	138334	38	807	35		
	*trn*I-GAU	-	138399	139418	42	943	35		
	*ndh*B	+	144834	147046	775	680	758		
	*rpl*2	+	156190	157691	385	683	434		

P. salicina ‘Yinhong plum’	*trn*K-UUU	-	1810	4419	37	2538	35		
	*rps*16	-	5350	6483	40	864	230		
	*trn*G-UCC	+	9029	9814	23	714	49		
	*atp*F	-	12035	13332	145	743	410		
	*rpo*C1	-	21421	24226	434	754	1618		
	*ycf*3	-	44320	46312	124	723	230	763	153
	*trn*L-UAA	+	49316	49916	37	514	50		
	*trn*V-UAC	-	53422	54087	39	590	37		
	*clp*P	-	71949	73994	71	811	291	647	226
	*pet*B	+	76911	78313	6	755	642		
	*pet*D	+	78501	79727	8	744	475		
	*rpl*16	-	83237	84655	9	1011	399		
	*rpl*2	-	86433	87934	385	683	434		
	*ndh*B	-	97016	99228	775	680	758		
	*trn*I-GAU	+	104650	105669	42	943	35		
	*trn*A-UGC	+	105734	106613	38	807	35		
	*ndh*A	-	122756	124996	553	1149	539		
	*trn*A-UGC	-	137477	138356	38	807	35		
	*trn*I-GAU	-	138421	139440	42	943	35		
	*ndh*B	+	144862	147074	775	680	758		
	*rpl*2	+	156156	157657	385	683	434		
P. salicina ‘Fengtang plum’	*trn*K-UUU	-	1824	4433	37	2538	35		
	*rps*16	-	5364	6497	40	864	230		
	*trn*G-UCC	+	9043	9828	23	714	49		
	*atp*F	-	12049	13346	145	743	410		
	*rpo*C1	-	21434	24239	434	754	1618		
	*ycf*3	-	44333	46325	124	723	230	763	153
	*trn*L-UAA	+	49329	49929	37	514	50		
	*trn*V-UAC	-	53435	54100	39	590	37		
	*clp*P	-	71962	74007	71	811	291	647	226
	*pet*B	+	76924	78326	6	755	642		
	*pet*D	+	78514	79740	8	744	475		
	*rpl*16	-	83250	84668	9	1011	399		
	*rpl*2	-	86446	87947	385	683	434		
	*ndh*B	-	97029	99241	775	680	758		
	*trn*I-GAU	+	104663	105682	42	943	35		
	*trn*A-UGC	+	105747	106626	38	807	35		
	*ndh*A	-	122769	125009	553	1149	539		
	*trn*A-UGC	-	137490	138369	38	807	35		
	*trn*I-GAU	-	138434	139453	42	943	35		
	*ndh*B	+	144875	147087	775	680	758		
	*rpl*2	+	156169	157670	385	683	434		
P. salicina ‘Cuihong plum’	*trn*K-UUU	-	1824	4433	37	2538	35		

	*rps*16	-	5364	6497	40	864	230		
	*trn*G-UCC	+	9043	9828	23	714	49		
	*atp*F	-	12049	13346	145	743	410		
	*rpo*C1	-	21435	24240	434	754	1618		
	*ycf*3	-	44334	46326	124	723	230	763	153
	*trn*L-UAA	+	49331	49931	37	514	50		
	*trn*V-UAC	-	53437	54102	39	590	37		
	*clp*P	-	71963	74009	71	811	291	648	226
	*pet*B	+	76926	78327	6	754	642		
	*pet*D	+	78515	79741	8	744	475		
	*rpl*16	-	83251	84669	9	1011	399		
	*rpl*2	-	86447	87948	385	683	434		
	*ndh*B	-	97031	99243	775	680	758		
	*trn*I-GAU	+	104665	105684	42	943	35		
	*trn*A-UGC	+	105749	106628	38	807	35		
	*ndh*A	-	122771	125011	553	1149	539		
	*trn*A-UGC	-	137492	138371	38	807	35		
	*trn*I-GAU	-	138436	139455	42	943	35		
	*ndh*B	+	144877	147089	775	680	758		
	*rpl*2	+	156172	157673	385	683	434		
P. cerasifera ‘Hollywood’	*trn*K-UUU	-	1761	4368	37	2536	35		
	*rps*16	-	5318	6450	40	863	230		
	*trn*G-UCC	+	9005	9795	23	719	49		
	*atp*F	-	12028	13325	145	743	410		
	*rpo*C1	-	21445	24250	434	754	1618		
	*ycf*3	-	44377	46361	124	714	230	764	153
	*trn*L-UAA	+	49366	49966	37	514	50		
	*trn*V-UAC	-	53396	54061	39	590	37		
	*clp*P	-	72042	74087	71	806	291	652	226
	*pet*B	+	77011	78411	6	753	642		
	*pet*D	+	78599	79825	8	744	475		
	*rpl*16	-	83334	84753	9	1012	399		
	*rpl*2	-	86532	88033	385	683	434		
	*ndh*B	-	97157	99369	775	680	758		
	*trn*I-GAU	+	104785	105804	42	943	35		
	*trn*A-UGC	+	105869	106748	38	807	35		
	*ndh*A	-	122783	125023	553	1149	539		
	*trn*A-UGC	-	137492	138371	38	807	35		
	*trn*I-GAU	-	138436	139455	42	943	35		
	*ndh*B	+	144871	147083	775	680	758		
	*rpl*2	+	156207	157708	385	683	434		
P. domestica ‘Bingtang plum’	*trn*K-UUU	-	1713	4320	37	2536	35		
	*rps*16	-	5244	6381	40	868	230		

	*trn*G-UCC	+	8942	9730	23	717	49		
	*atp*F	-	11961	13258	145	743	410		
	*rpo*C1	-	21363	24168	434	754	1618		
	*ycf*3	-	44298	46281	124	714	230	763	153
	*trn*L-UAA	+	49250	49850	37	514	50		
	*trn*V-UAC	-	53292	53957	39	590	37		
	*clp*P	-	71953	74002	71	806	291	656	226
	*pet*B	+	76926	78327	6	754	642		
	*pet*D	+	78515	79741	8	744	475		
	*rpl*16	-	83249	84677	9	1021	399		
	*rpl*2	-	86458	87959	385	683	434		
	*ndh*B	-	97103	99315	775	680	758		
	*trn*I-GAU	+	104731	105750	42	943	35		
	*trn*A-UGC	+	105815	106694	38	807	35		
	*ndh*A	-	122756	124996	553	1149	539		
	*trn*A-UGC	-	137455	138334	38	807	35		
	*trn*I-GAU	-	138399	139418	42	943	35		
	*ndh*B	+	144834	147046	775	680	758		
	*rpl*2	+	156190	157691	385	683	434		
P. salicina 'No.2 Guofeng'	*trn*K-UUU	-	1724	4332	37	2537	35		
	*rps*16	-	5273	6408	40	866	230		
	*trn*G-UCC	+	8917	9708	23	720	49		
	*atp*F	-	11934	13232	145	744	410		
	*rpo*C1	-	21327	24132	434	754	1618		
	*ycf*3	-	44189	46182	124	723	230	764	153
	*trn*L-UAA	+	49180	49780	37	514	50		
	*trn*V-UAC	-	53243	53908	39	590	37		
	*clp*P	-	71890	73924	71	806	291	641	226
	*pet*B	+	76841	78242	6	754	642		
	*pet*D	+	78430	79656	8	744	475		
	*rpl*16	-	83168	84586	9	1011	399		
	*rpl*2	-	86367	87868	385	683	434		
	*ndh*B	-	96983	99195	775	680	758		
	*trn*I-GAU	+	104617	105636	42	943	35		
	*trn*A-UGC	+	105701	106580	38	807	35		
	*ndh*A	-	122718	124958	553	1149	539		
	*trn*A-UGC	-	137435	138314	38	807	35		
	*trn*I-GAU	-	138379	139398	42	943	35		
	*ndh*B	+	144820	147032	775	680	758		
	*rpl*2	+	156147	157648	385	683	434		

Table S4. Statistics on simple sequence repeats (SSRs) in the twelve plastomes.

Species	SSR nr.	SSR type	SSR	Size	Start	End
P. salicina 'Sanhua plum'	1	p1	(A)11	11	371	381
	2	p1	(T)11	11	1728	1738
	3	p1	(A)11	11	3039	3049
	4	p1	(T)11	11	3385	3395
	5	p1	(T)10	10	4148	4157
	6	p1	(A)13	13	5748	5760
	7	p1	(A)14	14	6952	6965
	8	p1	(A)16	16	8554	8569
	9	p1	(T)10	10	9619	9628
	10	p1	(T)19	19	10027	10045
	11	p1	(T)10	10	11979	11988
	12	p1	(T)13	13	14284	14296
	13	p1	(T)11	11	15007	15017
	14	p1	(A)15	15	16894	16908
	15	p1	(T)11	11	19088	19098
	16	p1	(T)10	10	26809	26818
	17	p1	(A)10	10	27889	27898
	18	p1	(T)10	10	29132	29141
	19	p1	(T)10	10	29715	29724
	20	p2	(AT)7	14	48482	48495
	21	p1	(T)10	10	48606	48615
	22	p1	(A)15	15	48939	48953
	23	p2	(TA)6	12	50760	50771
	24	p1	(T)10	10	56412	56421
	25	p1	(T)13	13	59094	59106
	26	p1	(T)11	11	62674	62684
	27	p1	(A)10	10	62821	62830
	28	p1	(A)11	11	65397	65407
	29	p1	(G)11	11	66588	66598
	30	p1	(A)17	17	67371	67387
	31	c	(T)10agaataagttattct(A)11	36	69253	69288
	32	p1	(T)19	19	69545	69563
	33	p1	(A)13	13	70283	70295
	34	p1	(T)14	14	72455	72468
	35	p1	(T)15	15	73483	73497
	36	p2	(AT)6	12	74136	74147
	37	p1	(T)17	17	77059	77075
	38	p1	(A)15	15	83097	83111
	39	p1	(T)14	14	84664	84677
	40	p1	(T)10	10	85463	85472

	41	p1	(G)13	13	96177	96189
	42	p1	(A)16	16	116909	116924
	43	c	(A)12tgaacct tagtattatttatta gtaaagtaatag tcttagtaaagta attataaattattc tttaatcaagag atttac(T)17	108	122042	122149
	44	p1	(T)10	10	126646	126655
	45	p1	(T)11	11	129654	129664
	46	p1	(A)19	19	130279	130297
	47	p1	(T)10	10	130669	130678
	48	p1	(C)13	13	147805	147817
P. salicina 'Wanshuang plum'	1	p1	(A)11	11	396	406
	2	p1	(T)11	11	1753	1763
	3	p1	(A)11	11	3064	3074
	4	p1	(T)11	11	3410	3420
	5	p1	(A)10	10	3901	3910
	6	p1	(T)10	10	4175	4184
	7	p1	(A)13	13	5775	5787
	8	p1	(A)14	14	6979	6992
	9	p1	(A)10	10	7830	7839
	10	p1	(A)16	16	8582	8597
	11	p1	(T)10	10	9647	9656
	12	p1	(T)19	19	10055	10073
	13	p1	(T)10	10	12007	12016
	14	p1	(T)10	10	13467	13476
	15	p1	(T)13	13	14313	14325
	16	p1	(T)11	11	15036	15046
	17	p1	(A)16	16	16923	16938
	18	p1	(T)11	11	19118	19128
	19	p1	(T)10	10	26839	26848
	20	p1	(A)10	10	27919	27928
	21	p1	(T)10	10	29162	29171
	22	p1	(T)10	10	29745	29754
	23	p2	(AT)7	14	48512	48525
	24	p1	(T)10	10	48636	48645
	25	p1	(A)15	15	48969	48983
	26	p2	(TA)6	12	50790	50801
	27	p1	(T)10	10	56442	56451
	28	p1	(T)15	15	59124	59138
	29	p1	(T)11	11	62706	62716
	30	p1	(A)10	10	62853	62862

	31	p1	(A)11	11	65429	65439
	32	p1	(G)12	12	66654	66665
	33	p1	(A)17	17	67438	67454
	34	c	(T)10agaata agttattct(A)1 0	35	69320	69354
	35	p1	(T)14	14	69611	69624
	36	p1	(A)13	13	70344	70356
	37	p1	(T)14	14	72516	72529
	38	p1	(T)14	14	73544	73557
	39	p2	(AT)6	12	74196	74207
	40	p1	(T)16	16	77119	77134
	41	p1	(A)16	16	83156	83171
	42	p1	(T)13	13	84724	84736
	43	p1	(T)10	10	85522	85531
	44	p1	(G)13	13	96236	96248
	45	p1	(A)16	16	116973	116988
	46	c	(A)12tgaacct tagtattatttatta gtaaagtaatag tcttagtaaagta attataaattattc tttaatcaagag atttac(T)13	104	122106	122209
	47	p1	(T)10	10	126706	126715
	48	p1	(T)11	11	129714	129724
	49	p1	(A)19	19	130339	130357
	50	p1	(T)10	10	130729	130738
	51	p1	(A)10	10	130841	130850
	52	p1	(C)13	13	147868	147880
P. salicina ‘Wuyuecui’	1	p1	(T)12	12	1652	1663
	2	p1	(A)11	11	2964	2974
	3	p1	(T)11	11	3310	3320
	4	p1	(A)10	10	3801	3810
	5	p1	(A)13	13	6858	6870
	6	p1	(A)10	10	8466	8475
	7	p1	(T)10	10	9527	9536
	8	p1	(T)16	16	9935	9950
	9	p1	(T)10	10	11892	11901
	10	p1	(A)10	10	12776	12785
	11	p1	(T)13	13	14213	14225
	12	p1	(T)11	11	14936	14946
	13	p1	(T)11	11	19010	19020
	14	p1	(T)10	10	26731	26740

15	p1	(A)10	10	27811	27820
16	p1	(T)10	10	29082	29091
17	p1	(T)10	10	29593	29602
18	p1	(T)10	10	44487	44496
19	p2	(AT)7	14	48361	48374
20	p1	(T)10	10	48485	48494
21	p1	(A)16	16	48818	48833
22	p1	(T)10	10	50030	50039
23	p1	(T)10	10	52286	52295
24	p1	(T)10	10	56249	56258
25	p1	(T)11	11	58723	58733
26	p1	(T)16	16	58933	58948
27	p1	(C)15	15	60847	60861
28	p1	(T)12	12	62670	62681
29	p1	(A)10	10	62818	62827
30	p1	(A)11	11	65394	65404
31	p1	(G)12	12	66581	66592
32	p1	(A)11	11	67365	67375
33	p1	(A)11	11	67964	67974
34	c	(T)10agaataagttattct(A)11	36	69243	69278
35	p1	(T)17	17	69535	69551
36	p1	(A)13	13	70271	70283
37	p1	(T)16	16	72435	72450
38	p1	(T)11	11	73464	73474
39	p2	(AT)6	12	74113	74124
40	p1	(T)16	16	77036	77051
41	p1	(A)15	15	83075	83089
42	p1	(T)16	16	84642	84657
43	p1	(T)10	10	85443	85452
44	p1	(G)13	13	96157	96169
45	c	(A)11taaaatatttttcttaattaattgtttctgattcaccggttcttatttgttttctgttgaaaggggtcagttaat(A)10	96	114719	114814
46	p1	(A)14	14	116926	116939
47	c	(A)10tgaaccttagtattatttattagtaaagtaatagtcttagtaaagta	101	122058	122158

			attataaattattc tttaatcaagag atttac(T)12			
	48	p1	(T)11	11	129662	129672
	49	p1	(A)19	19	130287	130305
	50	p1	(T)10	10	130677	130686
	51	p1	(C)13	13	147846	147858
P. salicina 'Oishiwase'	1	p1	(T)14	14	1647	1660
	2	p1	(A)11	11	2961	2971
	3	p1	(T)11	11	3307	3317
	4	p1	(A)10	10	4930	4939
	5	p1	(A)12	12	6849	6860
	6	p1	(A)10	10	8456	8465
	7	p1	(T)10	10	9517	9526
	8	p1	(T)16	16	9925	9940
	9	p1	(T)10	10	11882	11891
	10	p1	(A)11	11	12766	12776
	11	p1	(T)12	12	14204	14215
	12	p1	(T)11	11	14926	14936
	13	p1	(T)11	11	19000	19010
	14	p1	(T)10	10	26721	26730
	15	p1	(A)10	10	27801	27810
	16	p1	(T)10	10	29072	29081
	17	p1	(T)10	10	29583	29592
	18	c	(AT)7aaa(AT)6	29	48320	48348
	19	p1	(T)10	10	48459	48468
	20	p1	(A)15	15	48792	48806
	21	p1	(T)10	10	52264	52273
	22	p1	(T)10	10	56247	56256
	23	p1	(T)10	10	58721	58730
	24	p1	(T)17	17	58930	58946
	25	p1	(C)12	12	60841	60852
	26	p1	(T)16	16	62661	62676
	27	p1	(A)10	10	62813	62822
	28	p1	(A)12	12	65389	65400
	29	p1	(G)11	11	66576	66586
	30	p1	(A)11	11	67359	67369
	31	p1	(A)10	10	67958	67967
	32	c	(T)10agaata acttattct(A)1 1	36	69236	69271
	33	p1	(T)17	17	69528	69544
	34	p1	(A)13	13	70264	70276

	35	p1	(T)16	16	72440	72455
	36	p1	(T)12	12	73469	73480
	37	p2	(AT)6	12	74125	74136
	38	p1	(T)15	15	77048	77062
	39	p1	(A)15	15	83088	83102
	40	p1	(T)16	16	84656	84671
	41	p1	(T)10	10	85457	85466
	42	p1	(G)14	14	96171	96184
	43	c	(A)10taaaatatttttcttaattaattatttctgattcaccggttcttatttgttttctgttgaaaggggtcagttaat(A)10	95	114728	114822
	44	p1	(A)15	15	116940	116954
	45	c	(A)11tgaaccttagtattatttattagtaaagtaatagtcttagtaaagtaattataaattattctttaatcaagagatttac(T)12	102	122073	122174
	46	p1	(T)11	11	129678	129688
	47	p1	(A)19	19	130303	130321
	48	p1	(T)10	10	130693	130702
	49	p1	(C)14	14	147856	147869
P. simonii ‘Weiwang’	1	p1	(A)11	11	396	406
	2	p1	(T)11	11	1753	1763
	3	p1	(A)11	11	3064	3074
	4	p1	(T)11	11	3410	3420
	5	p1	(A)10	10	3901	3910
	6	p1	(T)10	10	4175	4184
	7	p1	(A)13	13	5775	5787
	8	p1	(A)14	14	6979	6992
	9	p1	(A)10	10	7830	7839
	10	p1	(A)16	16	8582	8597
	11	p1	(T)10	10	9647	9656
	12	p1	(T)19	19	10055	10073
	13	p1	(T)10	10	12007	12016
	14	p1	(T)13	13	14312	14324
	15	p1	(T)11	11	15035	15045
	16	p1	(A)15	15	16922	16936
	17	p1	(T)11	11	19116	19126

	18	p1	(T)10	10	26837	26846
	19	p1	(A)10	10	27917	27926
	20	p1	(T)10	10	29160	29169
	21	p1	(T)10	10	29743	29752
	22	p2	(AT)7	14	48510	48523
	23	p1	(T)10	10	48634	48643
	24	p1	(A)15	15	48967	48981
	25	p2	(TA)6	12	50788	50799
	26	p1	(T)10	10	56440	56449
	27	p1	(T)14	14	59122	59135
	28	p1	(T)11	11	62703	62713
	29	p1	(A)10	10	62850	62859
	30	p1	(A)11	11	65426	65436
	31	p1	(G)12	12	66651	66662
	32	p1	(A)17	17	67435	67451
	33	c	(T)10agaataagttattct(A)10	35	69317	69351
	34	p1	(T)14	14	69608	69621
	35	p1	(A)13	13	70341	70353
	36	p1	(T)14	14	72513	72526
	37	p1	(T)15	15	73541	73555
	38	p2	(AT)6	12	74194	74205
	39	p1	(T)17	17	77117	77133
	40	p1	(A)15	15	83155	83169
	41	p1	(T)13	13	84722	84734
	42	p1	(T)10	10	85520	85529
	43	p1	(G)13	13	96234	96246
	44	p1	(A)16	16	116971	116986
	45	c	(A)12tgaaccttagtattatttattagtaaagtaatagtcttagtaaagtaattataaattattctttaatcaagagatttac(T)13	104	122104	122207
	46	p1	(T)10	10	126704	126713
	47	p1	(T)11	11	129712	129722
	48	p1	(A)19	19	130337	130355
	49	p1	(T)10	10	130727	130736
	50	p1	(A)10	10	130839	130848
	51	p1	(C)13	13	147866	147878
P. domestica ‘Richard Early’	1	p1	(A)10	10	245	254
	2	p1	(A)11	11	2953	2963

3	p1	(T)10	10	4062	4071
4	p1	(A)10	10	4730	4739
5	p1	(A)16	16	5656	5671
6	p1	(A)13	13	6865	6877
7	p1	(A)13	13	7721	7733
8	p1	(T)13	13	9546	9558
9	p1	(T)14	14	9957	9970
10	p1	(T)10	10	13379	13388
11	p1	(T)14	14	14241	14254
12	p1	(T)13	13	14965	14977
13	p1	(A)12	12	16855	16866
14	p1	(T)11	11	19046	19056
15	p1	(T)10	10	26767	26776
16	p1	(A)11	11	27847	27857
17	p2	(AT)6	12	48460	48471
18	p1	(T)10	10	48582	48591
19	p1	(A)16	16	48888	48903
20	p1	(T)10	10	50100	50109
21	p1	(T)10	10	50906	50915
22	p1	(T)10	10	52256	52265
23	p1	(T)10	10	56306	56315
24	p1	(T)14	14	59025	59038
25	p1	(T)10	10	62750	62759
26	p1	(A)11	11	65471	65481
27	p1	(G)11	11	66658	66668
28	p1	(T)10	10	67101	67110
29	p1	(A)15	15	67443	67457
30	c	(T)10agaataagttattct(A)11	36	69323	69358
31	p1	(T)15	15	69609	69623
32	p1	(A)13	13	70343	70355
33	p1	(T)14	14	72514	72527
34	p1	(A)10	10	72711	72720
35	p1	(T)12	12	73542	73553
36	p2	(AT)6	12	74191	74202
37	p1	(T)16	16	77121	77136
38	p2	(AT)6	12	79765	79776
39	p1	(A)15	15	83156	83170
40	p1	(A)10	10	83978	83987
41	p1	(T)11	11	84733	84743
42	p1	(T)10	10	85534	85543
43	p1	(G)13	13	96249	96261
44	c	(A)14taaaata	99	114812	114910

		tttttcttaattaatt gtttctgattcac cggttcttatttgtt ttctgttgaaagg ggtcagttaat(A)10			
	45	p1	(T)10	10	129701	129710
	46	p1	(A)19	19	130325	130343
	47	p1	(T)10	10	130715	130724
	48	p1	(C)13	13	147888	147900
P. salicina 'Yinhong plum'	1	p1	(A)11	11	382	392
	2	p1	(T)11	11	1739	1749
	3	p1	(A)11	11	3050	3060
	4	p1	(T)11	11	3396	3406
	5	p1	(A)10	10	3887	3896
	6	p1	(T)10	10	4161	4170
	7	p1	(A)13	13	5761	5773
	8	p1	(A)14	14	6965	6978
	9	p1	(A)10	10	7816	7825
	10	p1	(A)16	16	8568	8583
	11	p1	(T)10	10	9633	9642
	12	p1	(T)19	19	10041	10059
	13	p1	(T)10	10	11993	12002
	14	p1	(T)10	10	13453	13462
	15	p1	(T)13	13	14299	14311
	16	p1	(T)11	11	15022	15032
	17	p1	(A)16	16	16909	16924
	18	p1	(T)11	11	19104	19114
	19	p1	(T)10	10	26825	26834
	20	p1	(A)10	10	27905	27914
	21	p1	(T)10	10	29148	29157
	22	p1	(T)10	10	29731	29740
	23	p2	(AT)7	14	48498	48511
	24	p1	(T)10	10	48622	48631
	25	p1	(A)15	15	48955	48969
	26	p2	(TA)6	12	50776	50787
	27	p1	(T)10	10	56428	56437
	28	p1	(T)15	15	59110	59124
	29	p1	(T)11	11	62692	62702
	30	p1	(A)10	10	62839	62848
	31	p1	(A)11	11	65415	65425
	32	p1	(G)12	12	66640	66651
	33	p1	(A)17	17	67424	67440
	34	c	(T)10agaata	35	69306	69340

			agttattct(A)1<br>0			
	35	p1	(T)14	14	69597	69610
	36	p1	(A)13	13	70330	70342
	37	p1	(T)14	14	72502	72515
	38	p1	(T)15	15	73530	73544
	39	p2	(AT)6	12	74183	74194
	40	p1	(T)17	17	77106	77122
	41	p1	(A)15	15	83144	83158
	42	p1	(T)13	13	84711	84723
	43	p1	(T)10	10	85509	85518
	44	p1	(G)13	13	96223	96235
	45	p1	(A)16	16	116960	116975
	46	c	(A)12tgaacct<br>tagtattatttatta<br>gtaaagtaatag<br>tcttagtaaagta<br>attataaattattc<br>tttaatcaagag<br>atttac(T)13	104	122093	122196
	47	p1	(T)10	10	126693	126702
	48	p1	(T)11	11	129701	129711
	49	p1	(A)19	19	130326	130344
	50	p1	(T)10	10	130716	130725
	51	p1	(A)10	10	130828	130837
	52	p1	(C)13	13	147855	147867
P. salicina 'Fengtang plum'	1	p1	(A)11	11	396	406
	2	p1	(T)11	11	1753	1763
	3	p1	(A)11	11	3064	3074
	4	p1	(T)11	11	3410	3420
	5	p1	(A)10	10	3901	3910
	6	p1	(T)10	10	4175	4184
	7	p1	(A)13	13	5775	5787
	8	p1	(A)14	14	6979	6992
	9	p1	(A)10	10	7830	7839
	10	p1	(A)16	16	8582	8597
	11	p1	(T)10	10	9647	9656
	12	p1	(T)19	19	10055	10073
	13	p1	(T)10	10	12007	12016
	14	p1	(T)10	10	13467	13476
	15	p1	(T)12	12	14313	14324
	16	p1	(T)11	11	15035	15045
	17	p1	(A)16	16	16922	16937
	18	p1	(T)11	11	19117	19127

	19	p1	(T)10	10	26838	26847
	20	p1	(A)10	10	27918	27927
	21	p1	(T)10	10	29161	29170
	22	p1	(T)10	10	29744	29753
	23	p2	(AT)7	14	48511	48524
	24	p1	(T)10	10	48635	48644
	25	p1	(A)15	15	48968	48982
	26	p2	(TA)6	12	50789	50800
	27	p1	(T)10	10	56441	56450
	28	p1	(T)15	15	59123	59137
	29	p1	(T)11	11	62705	62715
	30	p1	(A)10	10	62852	62861
	31	p1	(A)11	11	65428	65438
	32	p1	(G)12	12	66653	66664
	33	p1	(A)17	17	67437	67453
	34	c	(T)10agaataagttattct(A)10	35	69319	69353
	35	p1	(T)14	14	69610	69623
	36	p1	(A)13	13	70343	70355
	37	p1	(T)14	14	72515	72528
	38	p1	(T)15	15	73543	73557
	39	p2	(AT)6	12	74196	74207
	40	p1	(T)17	17	77119	77135
	41	p1	(A)15	15	83157	83171
	42	p1	(T)13	13	84724	84736
	43	p1	(T)10	10	85522	85531
	44	p1	(G)13	13	96236	96248
	45	p1	(A)16	16	116973	116988
	46	c	(A)12tgaaccttagtattatttattagtaaagtaatagtcttagtaaagtaattataaattattctttaatcaagagatttac(T)13	104	122106	122209
	47	p1	(T)10	10	126706	126715
	48	p1	(T)11	11	129714	129724
	49	p1	(A)19	19	130339	130357
	50	p1	(T)10	10	130729	130738
	51	p1	(A)10	10	130841	130850
	52	p1	(C)13	13	147868	147880
P. salicina ‘Cuihong plum’	1	p1	(A)11	11	396	406
	2	p1	(T)11	11	1753	1763

3	p1	(A)11	11	3064	3074
4	p1	(T)11	11	3410	3420
5	p1	(A)10	10	3901	3910
6	p1	(T)10	10	4175	4184
7	p1	(A)13	13	5775	5787
8	p1	(A)14	14	6979	6992
9	p1	(A)10	10	7830	7839
10	p1	(A)16	16	8582	8597
11	p1	(T)10	10	9647	9656
12	p1	(T)19	19	10055	10073
13	p1	(T)10	10	12007	12016
14	p1	(T)10	10	13467	13476
15	p1	(T)13	13	14313	14325
16	p1	(T)11	11	15036	15046
17	p1	(A)16	16	16923	16938
18	p1	(T)11	11	19118	19128
19	p1	(T)10	10	26839	26848
20	p1	(A)10	10	27919	27928
21	p1	(T)10	10	29162	29171
22	p1	(T)10	10	29745	29754
23	p2	(AT)7	14	48512	48525
24	p1	(T)10	10	48636	48645
25	p1	(A)16	16	48969	48984
26	p2	(TA)6	12	50791	50802
27	p1	(T)10	10	56443	56452
28	p1	(T)14	14	59125	59138
29	p1	(T)11	11	62706	62716
30	p1	(A)10	10	62853	62862
31	p1	(A)11	11	65429	65439
32	p1	(G)12	12	66654	66665
33	p1	(A)17	17	67438	67454
34	c	(T)10agaata agttattct(A)10	35	69320	69354
35	p1	(T)14	14	69611	69624
36	p1	(A)13	13	70344	70356
37	p1	(T)15	15	72516	72530
38	p1	(T)15	15	73545	73559
39	p2	(AT)6	12	74198	74209
40	p1	(T)16	16	77121	77136
41	p1	(A)15	15	83158	83172
42	p1	(T)13	13	84725	84737
43	p1	(T)10	10	85523	85532
44	p1	(G)14	14	96237	96250

	45	p1	(A)16	16	116975	116990
	46	c	(A)12tgaacct tagtattatttatta gtaaagtaatag tcttagtaaagta attataaattattc tttaatcaagag atttac(T)13	104	122108	122211
	47	p1	(T)10	10	126708	126717
	48	p1	(T)11	11	129716	129726
	49	p1	(A)19	19	130341	130359
	50	p1	(T)10	10	130731	130740
	51	p1	(A)10	10	130843	130852
	52	p1	(C)14	14	147870	147883
P. cerasifera 'Hollywood'	1	c	(A)10tatagaa caatatagtaaa gttaagtagtaa at(A)10	53	290	342
	2	p1	(T)10	10	1691	1700
	3	p1	(A)11	11	3001	3011
	4	p1	(T)11	11	3347	3357
	5	p1	(A)10	10	3838	3847
	6	p1	(A)12	12	5729	5740
	7	p1	(A)13	13	6934	6946
	8	p1	(A)13	13	7785	7797
	9	p1	(T)15	15	9609	9623
	10	p1	(T)15	15	10022	10036
	11	p1	(T)10	10	13446	13455
	12	p1	(T)14	14	14308	14321
	13	p1	(T)12	12	15032	15043
	14	p1	(A)12	12	16922	16933
	15	p1	(T)11	11	19113	19123
	16	p1	(T)10	10	26849	26858
	17	p1	(A)11	11	27929	27939
	18	p2	(TA)6	12	38247	38258
	19	p1	(T)10	10	44675	44684
	20	c	(AT)6aaaata atatatataaaat ataaa(AT)6	48	48540	48587
	21	p1	(T)10	10	48699	48708
	22	p1	(A)15	15	49005	49019
	23	p2	(TA)6	12	50826	50837
	24	p1	(T)10	10	52357	52366
	25	p1	(T)10	10	56402	56411

	26	p1	(T)14	14	59105	59118
	27	p1	(T)10	10	62820	62829
	28	p1	(A)11	11	65541	65551
	29	p1	(G)14	14	66728	66741
	30	p1	(T)10	10	67174	67183
	31	p1	(A)16	16	67516	67531
	32	c	(T)10agaata agttattct(A)11	36	69397	69432
	33	p1	(T)17	17	69689	69705
	34	p1	(A)13	13	70425	70437
	35	p1	(A)11	11	71128	71138
	36	p1	(T)11	11	72603	72613
	37	p1	(T)12	12	73627	73638
	38	p2	(AT)6	12	74276	74287
	39	p1	(T)16	16	77206	77221
	40	p1	(A)12	12	83244	83255
	41	p1	(T)10	10	85608	85617
	42	p1	(G)15	15	96322	96336
	43	p1	(T)10	10	129728	129737
	44	p1	(A)19	19	130352	130370
	45	p1	(T)10	10	130742	130751
	46	p1	(C)15	15	147904	147918
P. domestica 'Bingtang plum'	1	p1	(A)10	10	245	254
	2	p1	(A)11	11	2953	2963
	3	p1	(T)10	10	4062	4071
	4	p1	(A)10	10	4730	4739
	5	p1	(A)16	16	5656	5671
	6	p1	(A)13	13	6865	6877
	7	p1	(A)13	13	7721	7733
	8	p1	(T)13	13	9546	9558
	9	p1	(T)14	14	9957	9970
	10	p1	(T)10	10	13379	13388
	11	p1	(T)14	14	14241	14254
	12	p1	(T)13	13	14965	14977
	13	p1	(A)12	12	16855	16866
	14	p1	(T)11	11	19046	19056
	15	p1	(T)10	10	26767	26776
	16	p1	(A)11	11	27847	27857
	17	p2	(AT)6	12	48460	48471
	18	p1	(T)10	10	48582	48591
	19	p1	(A)16	16	48888	48903
	20	p1	(T)10	10	50100	50109
	21	p1	(T)10	10	50906	50915

	22	p1	(T)10	10	52256	52265
	23	p1	(T)10	10	56306	56315
	24	p1	(T)14	14	59025	59038
	25	p1	(T)10	10	62750	62759
	26	p1	(A)11	11	65471	65481
	27	p1	(G)11	11	66658	66668
	28	p1	(T)10	10	67101	67110
	29	p1	(A)15	15	67443	67457
	30	c	(T)10agaataagttattct(A)11	36	69323	69358
	31	p1	(T)15	15	69609	69623
	32	p1	(A)13	13	70343	70355
	33	p1	(T)14	14	72514	72527
	34	p1	(A)10	10	72711	72720
	35	p1	(T)12	12	73542	73553
	36	p2	(AT)6	12	74191	74202
	37	p1	(T)16	16	77121	77136
	38	p2	(AT)6	12	79765	79776
	39	p1	(A)15	15	83156	83170
	40	p1	(A)10	10	83978	83987
	41	p1	(T)11	11	84733	84743
	42	p1	(T)10	10	85534	85543
	43	p1	(G)13	13	96249	96261
	44	c	(A)14taaaatatttttcttaattaattgtttctgattcaccggttcttatttgttttctgttgaaaggggtcagttaat(A)10	99	114812	114910
	45	p1	(T)10	10	129701	129710
	46	p1	(A)19	19	130325	130343
	47	p1	(T)10	10	130715	130724
	48	p1	(C)13	13	147888	147900
P. salicina ‘No.2 Guofeng’	1	p1	(T)12	12	1652	1663
	2	p1	(A)11	11	2964	2974
	3	p1	(T)11	11	3310	3320
	4	p1	(A)10	10	3801	3810
	5	p1	(A)13	13	6858	6870
	6	p1	(A)10	10	8466	8475
	7	p1	(T)10	10	9527	9536
	8	p1	(T)16	16	9935	9950
	9	p1	(T)10	10	11892	11901

10	p1	(A)10	10	12776	12785
11	p1	(T)13	13	14213	14225
12	p1	(T)11	11	14936	14946
13	p1	(T)11	11	19010	19020
14	p1	(T)10	10	26731	26740
15	p1	(A)10	10	27811	27820
16	p1	(T)10	10	29082	29091
17	p1	(T)10	10	29593	29602
18	p1	(T)10	10	44487	44496
19	p2	(AT)7	14	48361	48374
20	p1	(T)10	10	48485	48494
21	p1	(A)16	16	48818	48833
22	p1	(T)10	10	50030	50039
23	p1	(T)10	10	52286	52295
24	p1	(T)10	10	56249	56258
25	p1	(T)11	11	58723	58733
26	p1	(T)16	16	58933	58948
27	p1	(C)15	15	60847	60861
28	p1	(T)12	12	62670	62681
29	p1	(A)10	10	62818	62827
30	p1	(A)11	11	65394	65404
31	p1	(G)12	12	66581	66592
32	p1	(A)11	11	67365	67375
33	p1	(A)11	11	67964	67974
34	c	(T)10agaataagttattct(A)11	36	69243	69278
35	p1	(T)17	17	69535	69551
36	p1	(A)13	13	70271	70283
37	p1	(T)16	16	72435	72450
38	p1	(T)11	11	73464	73474
39	p2	(AT)6	12	74113	74124
40	p1	(T)16	16	77036	77051
41	p1	(A)15	15	83075	83089
42	p1	(T)16	16	84642	84657
43	p1	(T)10	10	85443	85452
44	p1	(G)13	13	96157	96169
45	c	(A)11taaaatatttttcttaattaattgtttctgattcaccggttcttatttgttttctgttgaaaggggtcagttaat(A)10	96	114719	114814

	46	p1	(A)14	14	116926	116939
	47	c	(A)10tgaacct tagtattatttatta gtaaagtaatag tcttagtaaagta attataaattattc tttaatcaagag atttac(T)12	101	122058	122158
	48	p1	(T)11	11	129662	129672
	49	p1	(A)19	19	130287	130305
	50	p1	(T)10	10	130677	130686
	51	p1	(C)13	13	147846	147858

Table S5. The list of accession numbers of the plastome sequences used in the phylogenetic analyses of the *Prunus*.

No.	Taxon	Family	Tribe	GenBank Accession Number
1	*Prunus salicina* 'Sanhua plum'	Rosaceae	Amygdaleae	MW406459
2	*Prunus salicina* 'Wanshuang plum'	Rosaceae	Amygdaleae	MW406460
3	*Prunus salicina* 'Wuyuecui'	Rosaceae	Amygdaleae	MW406461
4	*Prunus salicina* 'Oishiwase'	Rosaceae	Amygdaleae	MW406457
5	*Prunus simonii* 'Weiwang'	Rosaceae	Amygdaleae	MW406463
6	*Prunus domestica* 'Richard Early'	Rosaceae	Amygdaleae	MW406464
7	*Prunus salicina* 'Yinhong plum'	Rosaceae	Amygdaleae	MW406465
8	*Prunus salicina* 'Fengtang plum'	Rosaceae	Amygdaleae	MW406466
9	*Prunus salicina* 'Cuihong plum'	Rosaceae	Amygdaleae	MW406468
10	*Prunus cerasifera* 'Hollywood'	Rosaceae	Amygdaleae	MW406470
11	*Prunus domestica* 'Bingtang plum'	Rosaceae	Amygdaleae	MW406471
12	*Prunus salicina* 'No.2 Guofeng'	Rosaceae	Amygdaleae	MW406472
13	*Prunus salicina*	Rosaceae	Amygdaleae	NC_047442
14	*Prunus domestica*	Rosaceae	Amygdaleae	NC_050959
15	*Prunus kansuensis*	Rosaceae	Amygdaleae	NC_023956
16	*Prunus persica*	Rosaceae	Amygdaleae	NC_014697
17	*Prunus mira*	Rosaceae	Amygdaleae	NC_040125
18	*Prunus davidiana*	Rosaceae	Amygdaleae	NC_039735
19	*Prunus padus*	Rosaceae	Amygdaleae	NC_026982
20	*Prunus speciosa*	Rosaceae	Amygdaleae	NC 043921
21	*Prunus maximowiczii*	Rosaceae	Amygdaleae	NC_026981
22	*Prunus dielsiana*	Rosaceae	Amygdaleae	NC_056266
23	*Prunus pseudocerasus*	Rosaceae	Amygdaleae	NC_030599
24	*Prunus fruticosa*	Rosaceae	Amygdaleae	NC_054254
25	*Prunus avium*	Rosaceae	Amygdaleae	MK622380
26	*Prunus cerasoides*	Rosaceae	Amygdaleae	NC 035891
27	*Prunus tomentosa*	Rosaceae	Amygdaleae	NC_036394
28	*Prunus triloba*	Rosaceae	Amygdaleae	NC_046742
29	*Prunus mume*	Rosaceae	Amygdaleae	NC_023798
30	*Prunus humilis*	Rosaceae	Amygdaleae	NC_035880
31	*Prunus japonica*	Rosaceae	Amygdaleae	NC_053703
32	*Malus baccata*	Rosaceae	Maleae	NC_045389

Table S6. The two pairs of primers for the amplification of DNA barcodes

Primer ID	Sequence
LZ01_F	AGGATTGAGCCGAATACAATAA
LZ01_R	AAACGCAGGATCAGGATATAAT
LZ02_F	CCGCAGAACTACTTCGATATCTATT
LZ02_R	GGGAAAGGCATGTATATGTGATATTAAC
LZ03_F	GTTCAATGAGATAATGAGACAAAGG
LZ03_R	AACATGTTCTGTCCTGAATAGAG
LZ04_F	TTTGGACTACGCGGGAATC
LZ04_R	TCTCCATCACATTGTCGGAAC
LZ05_F	GTATCGGCCATTTGTCCTACTT
LZ05_R	TAGAGCTACGACACAATCAAACC
LZ06_F	GTGGATATCGAGGTTCCACAAG
LZ06_R	ACCAGGAACAGGACCTATCA
LZ07_F	GCTCTTGCTCTACTGGAATCTC
LZ07_R	AATGCTGCCAATGTTGCTATG
LZ08_F	GAAGCCAAACTATGCCCTAGA
LZ08_R	CATTCGTCGCTTCAGCAAAC

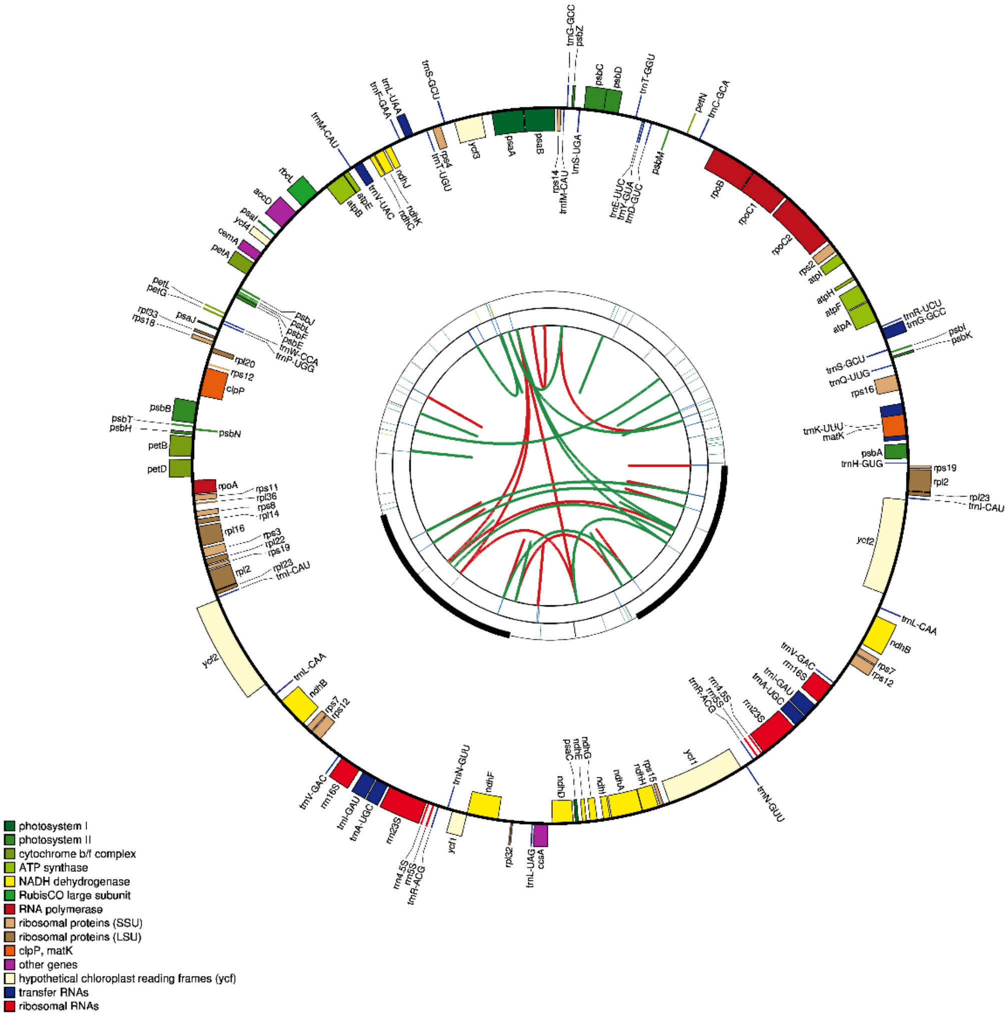

Figure S1. Genome map of *P. salicina* 'Wanshuang plum' plastome. The map has four rings, from the center outward, with red and green arcs on the first circle connecting forward and reverse repeats, respectively; the second ring shows tandem repeats marked with dashes; the third ring is a MISA-identified microsatellite sequence; and the fourth ring shows the gene structure on the plastome. The colors of these genes are classified according to their function, as shown in the lower left corner.

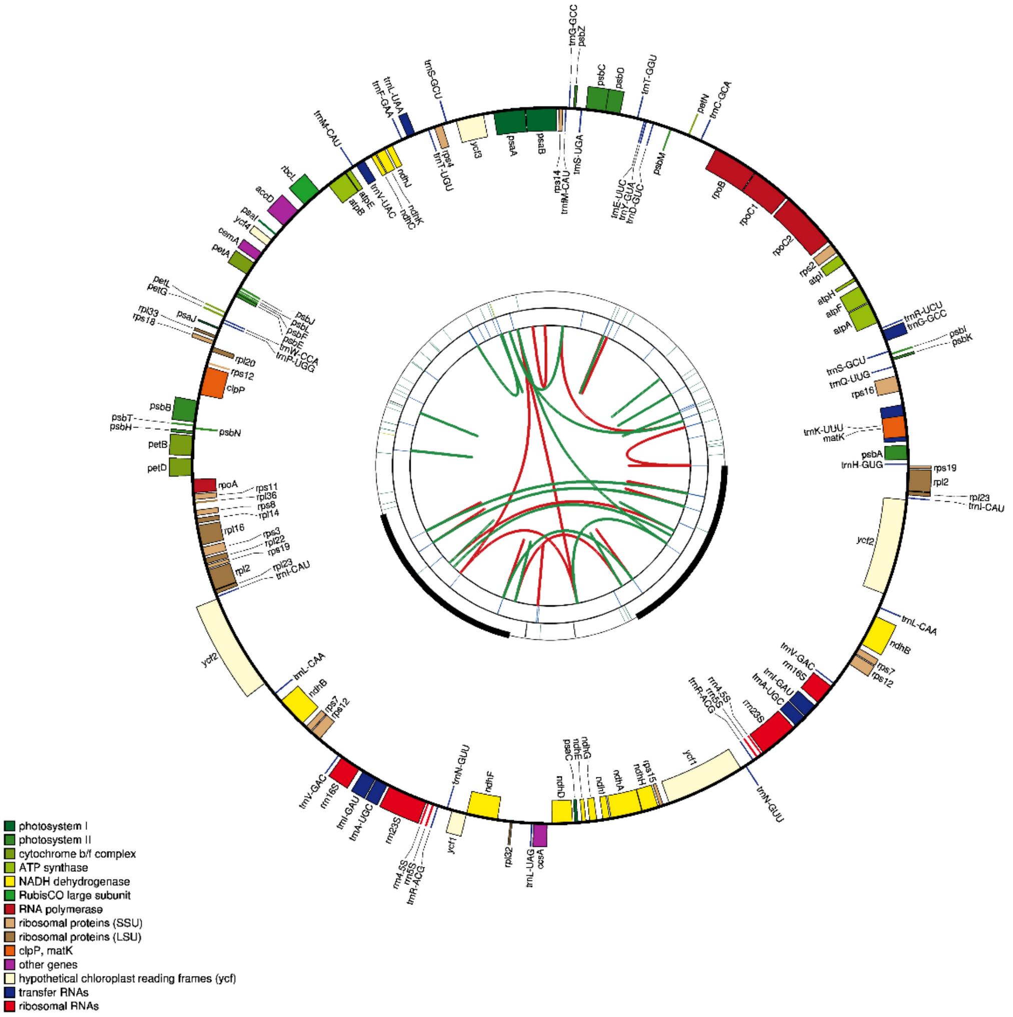

Figure S2. Genome map of *P. salicina* 'Wuyuecui' plastome. The map has four rings, from the center outward, with red and green arcs on the first circle connecting forward and reverse repeats, respectively; the second ring shows tandem repeats marked with dashes; the third ring is a MISA-identified microsatellite sequence; and the fourth ring shows the gene structure on the plastome. The colors of these genes are classified according to their function, as shown in the lower left corner.

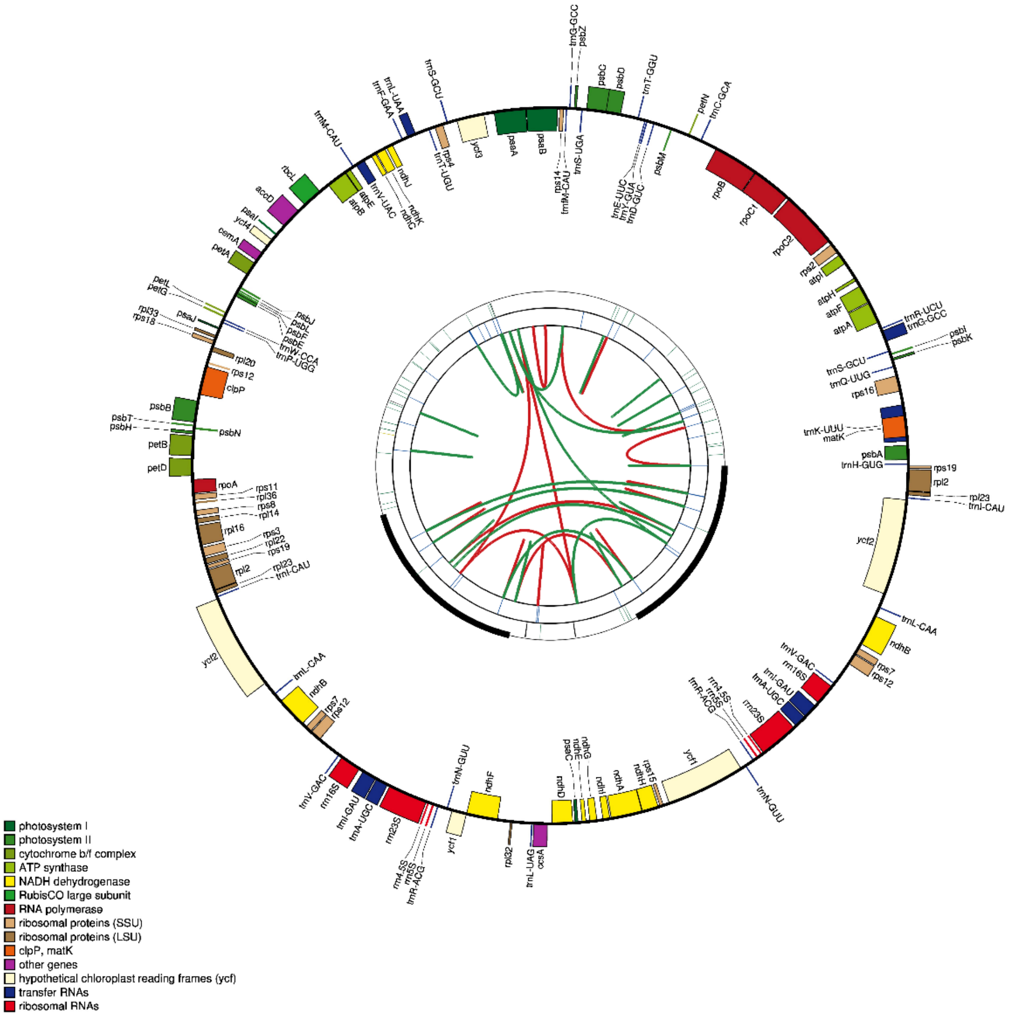

Figure S3. Genome map of *P. salicina* 'Oishiwase' plastome. The map has four rings, from the center outward, with red and green arcs on the first circle connecting forward and reverse repeats, respectively; the second ring shows tandem repeats marked with dashes; the third ring is a MISA-identified microsatellite sequence; and the fourth ring shows the gene structure on the plastome. The colors of these genes are classified according to their function, as shown in the lower left corner.

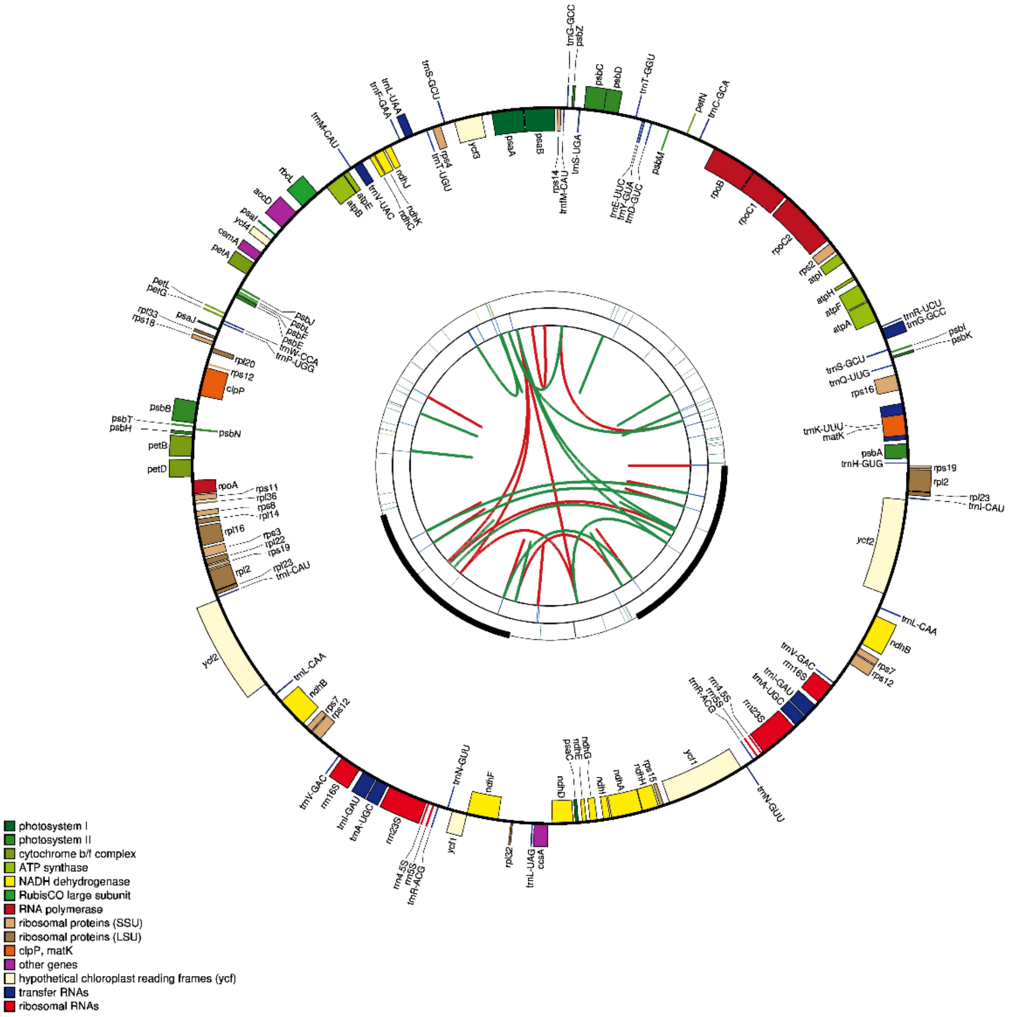

Figure S4. Genome map of *P. simonii* 'Weiwang' plastome. The map has four rings, from the center outward, with red and green arcs on the first circle connecting forward and reverse repeats, respectively; the second ring shows tandem repeats marked with dashes; the third ring is a MISA-identified microsatellite sequence; and the fourth ring shows the gene structure on the plastome. The colors of these genes are classified according to their function, as shown in the lower left corner.

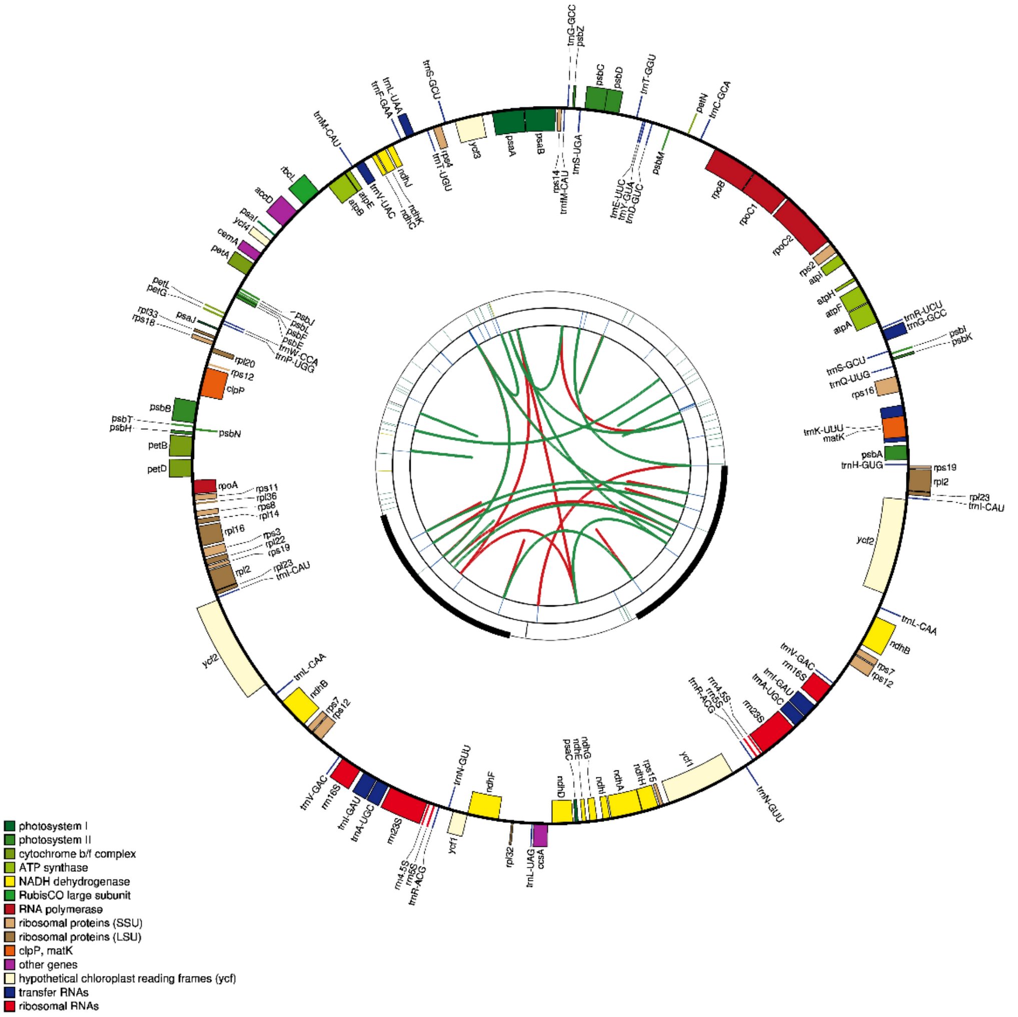

Figure S5. Genome map of *P. domestica* 'Richard Early' plastome. The map has four rings, from the center outward, with red and green arcs on the first circle connecting forward and reverse repeats, respectively; the second ring shows tandem repeats marked with dashes; the third ring is a MISA-identified microsatellite sequence; and the fourth ring shows the gene structure on the plastome. The colors of these genes are classified according to their function, as shown in the lower left corner.

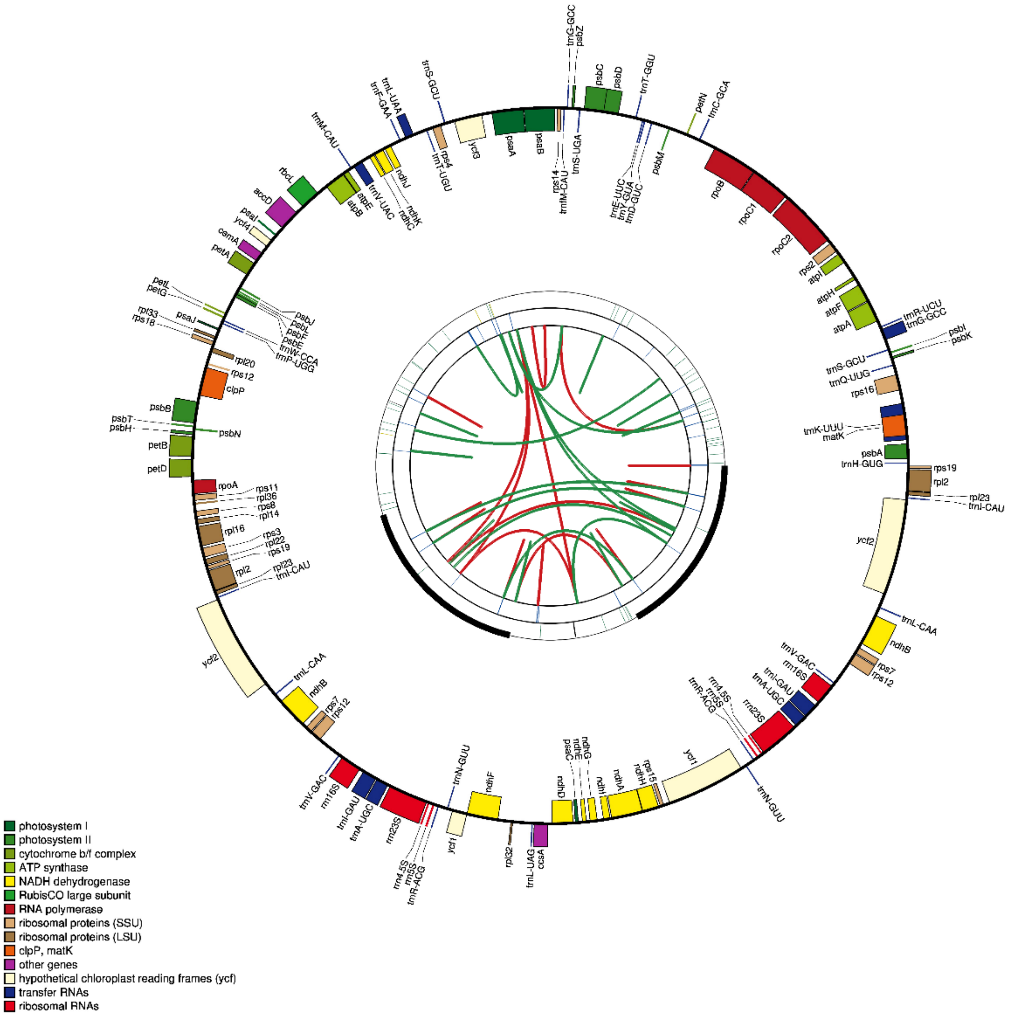

Figure S6. Genome map of *P. salicina* 'Yinhong plum' plastome. The map has four rings, from the center outward, with red and green arcs on the first circle connecting forward and reverse repeats, respectively; the second ring shows tandem repeats marked with dashes; the third ring is a MISA-identified microsatellite sequence; and the fourth ring shows the gene structure on the plastome. The colors of these genes are classified according to their function, as shown in the lower left corner.

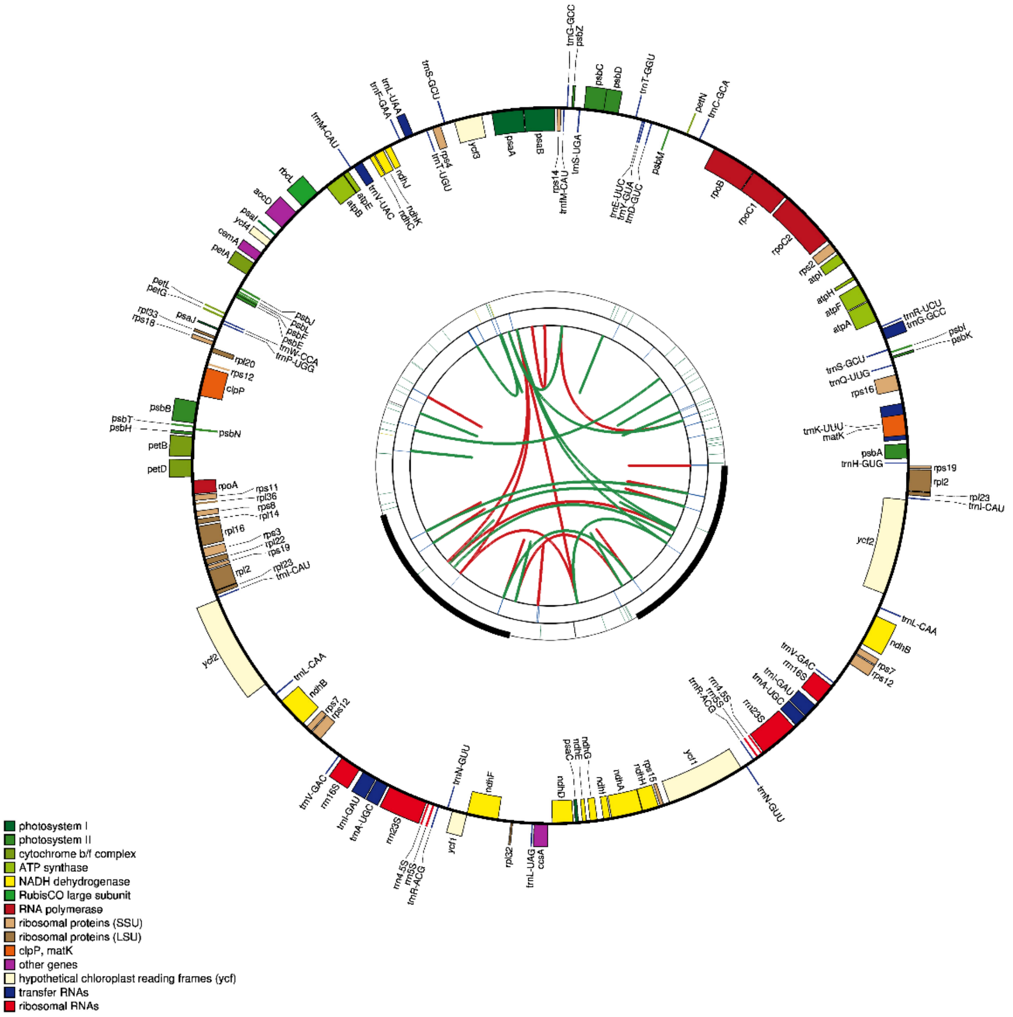

Figure S7. Genome map of *P. salicina* ' Fengtang plum' plastome. The map has four rings, from the center outward, with red and green arcs on the first circle connecting forward and reverse repeats, respectively; the second ring shows tandem repeats marked with dashes; the third ring is a MISA-identified microsatellite sequence; and the fourth ring shows the gene structure on the plastome. The colors of these genes are classified according to their function, as shown in the lower left corner.

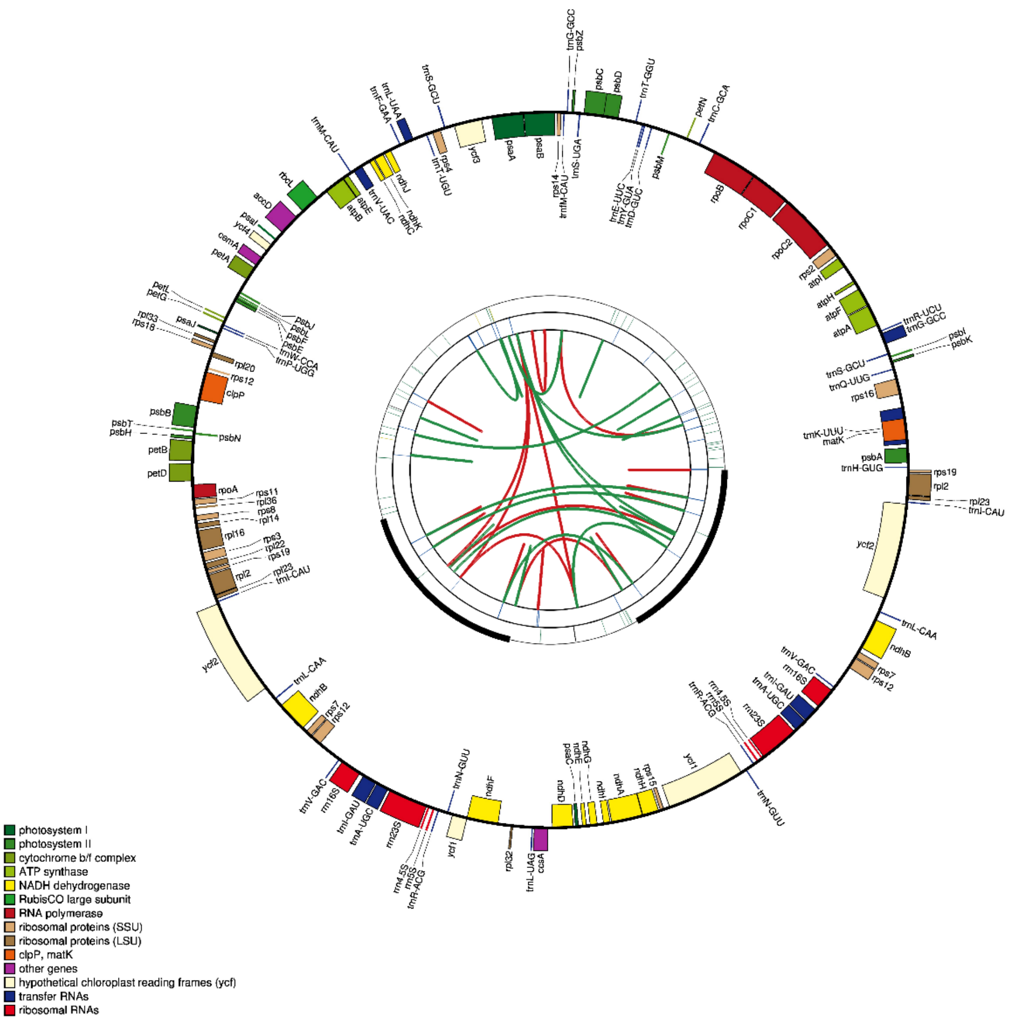

Figure S8. Genome map of *P. salicina* ' Cuihong plum' plastome. The map has four rings, from the center outward, with red and green arcs on the first circle connecting forward and reverse repeats, respectively; the second ring shows tandem repeats marked with dashes; the third ring is a MISA-identified microsatellite sequence; and the fourth ring shows the gene structure on the plastome. The colors of these genes are classified according to their function, as shown in the lower left corner.

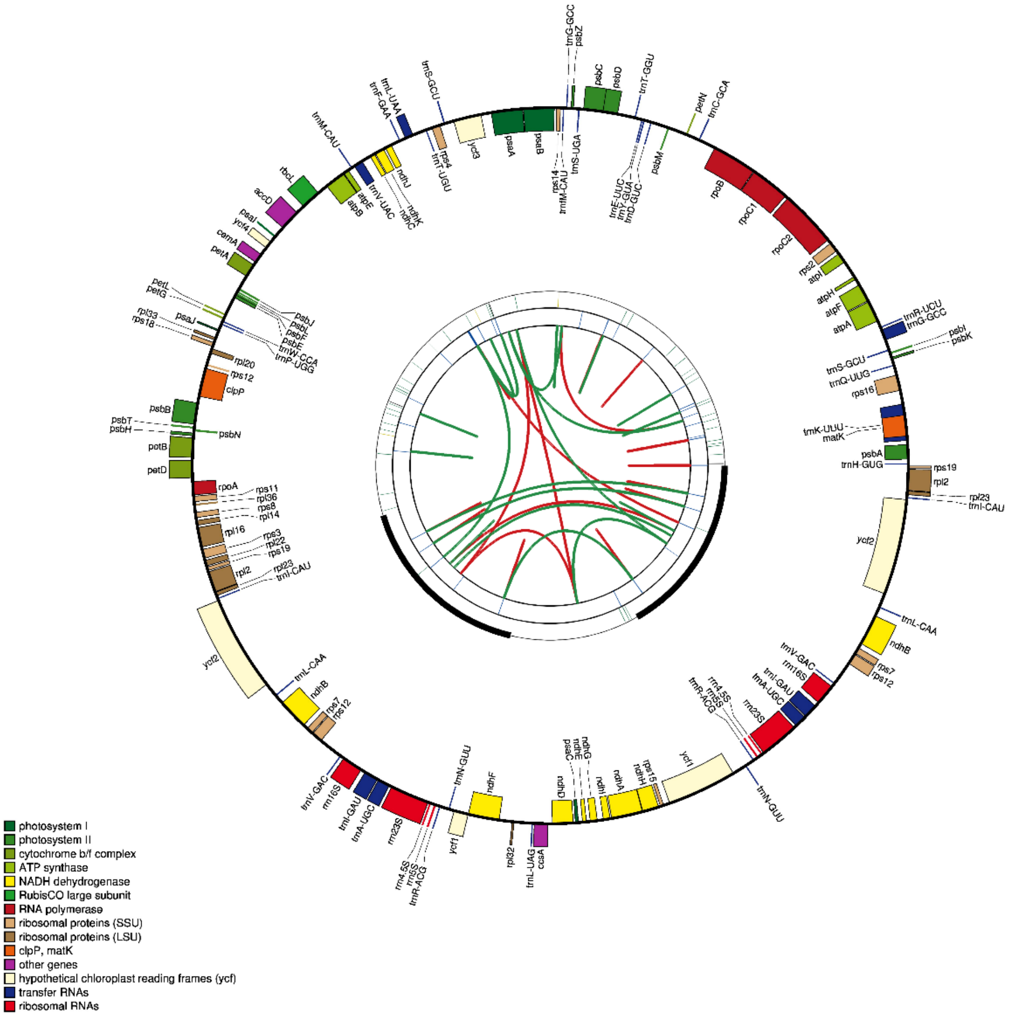

Figure S9. Genome map of *P. cerasifera* 'Hollywood' plastome. The map has four rings, from the center outward, with red and green arcs on the first circle connecting forward and reverse repeats, respectively; the second ring shows tandem repeats marked with dashes; the third ring is a MISA-identified microsatellite sequence; and the fourth ring shows the gene structure on the plastome. The colors of these genes are classified according to their function, as shown in the lower left corner.

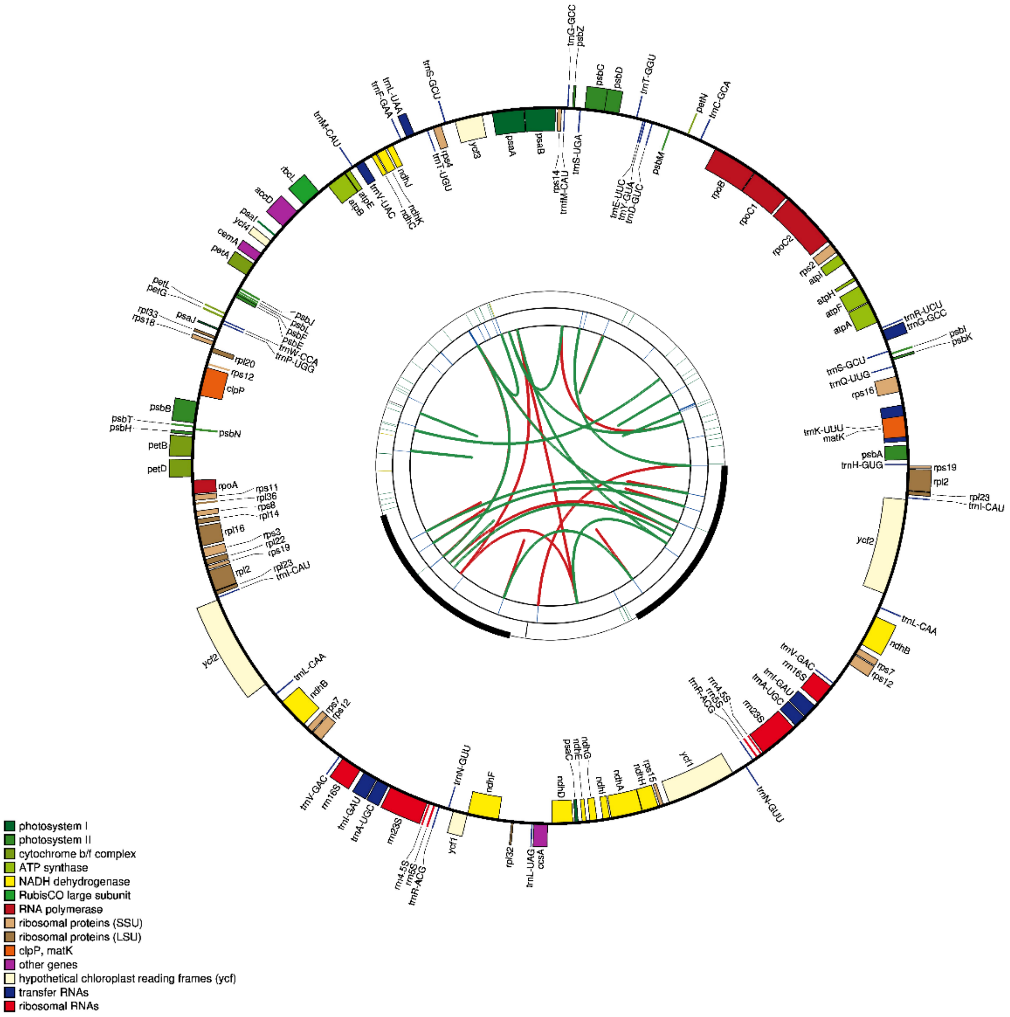

Figure S10. Genome map of *P. domestica* 'Bingtang plum' plastome. The map has four rings, from the center outward, with red and green arcs on the first circle connecting forward and reverse repeats, respectively; the second ring shows tandem repeats marked with dashes; the third ring is a MISA-identified microsatellite sequence; and the fourth ring shows the gene structure on the plastome. The colors of these genes are classified according to their function, as shown in the lower left corner.

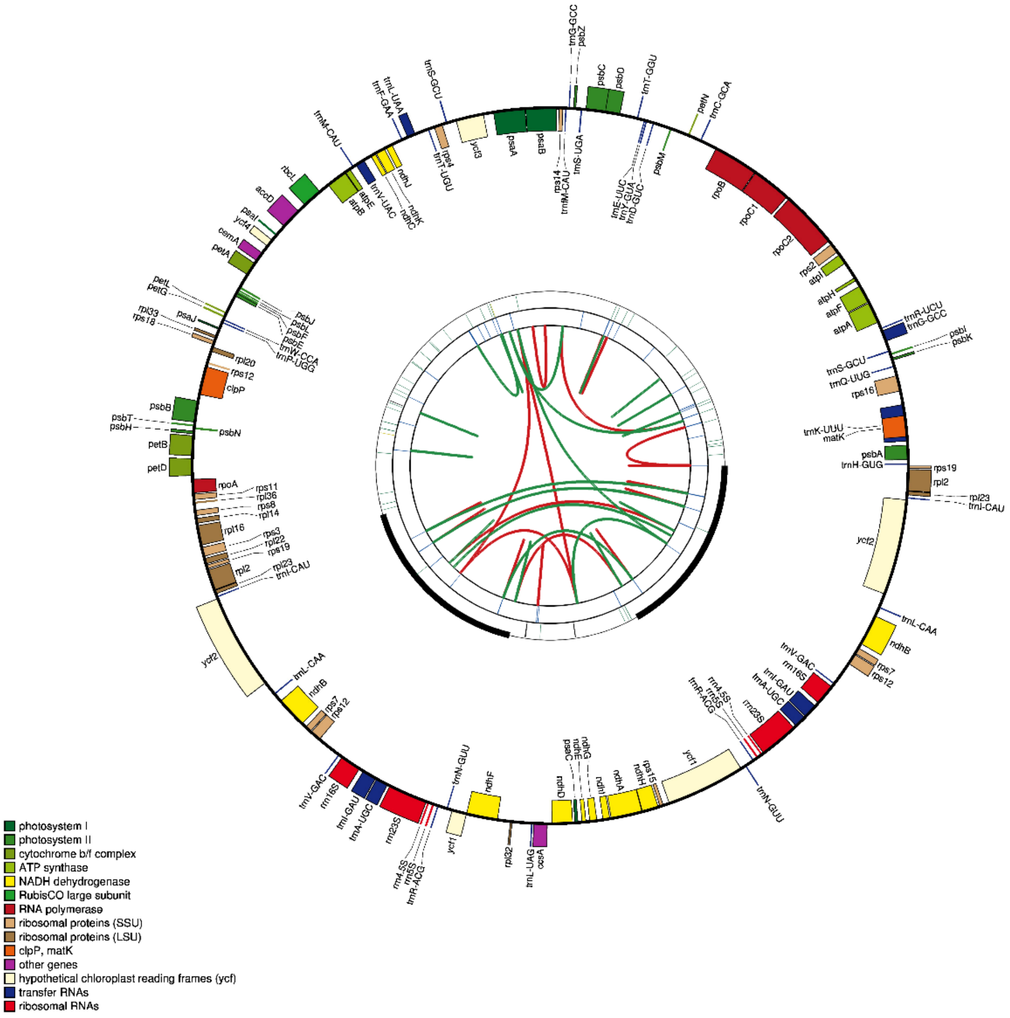

Figure S11. Genome map of *P. salicina* 'No.2 Guofeng' plastome. The map has four rings, from the center outward, with red and green arcs on the first circle connecting forward and reverse repeats, respectively; the second ring shows tandem repeats marked with dashes; the third ring is a MISA-identified microsatellite sequence; and the fourth ring shows the gene structure on the plastome. The colors of these genes are classified according to their function, as shown in the lower left corner.

A.

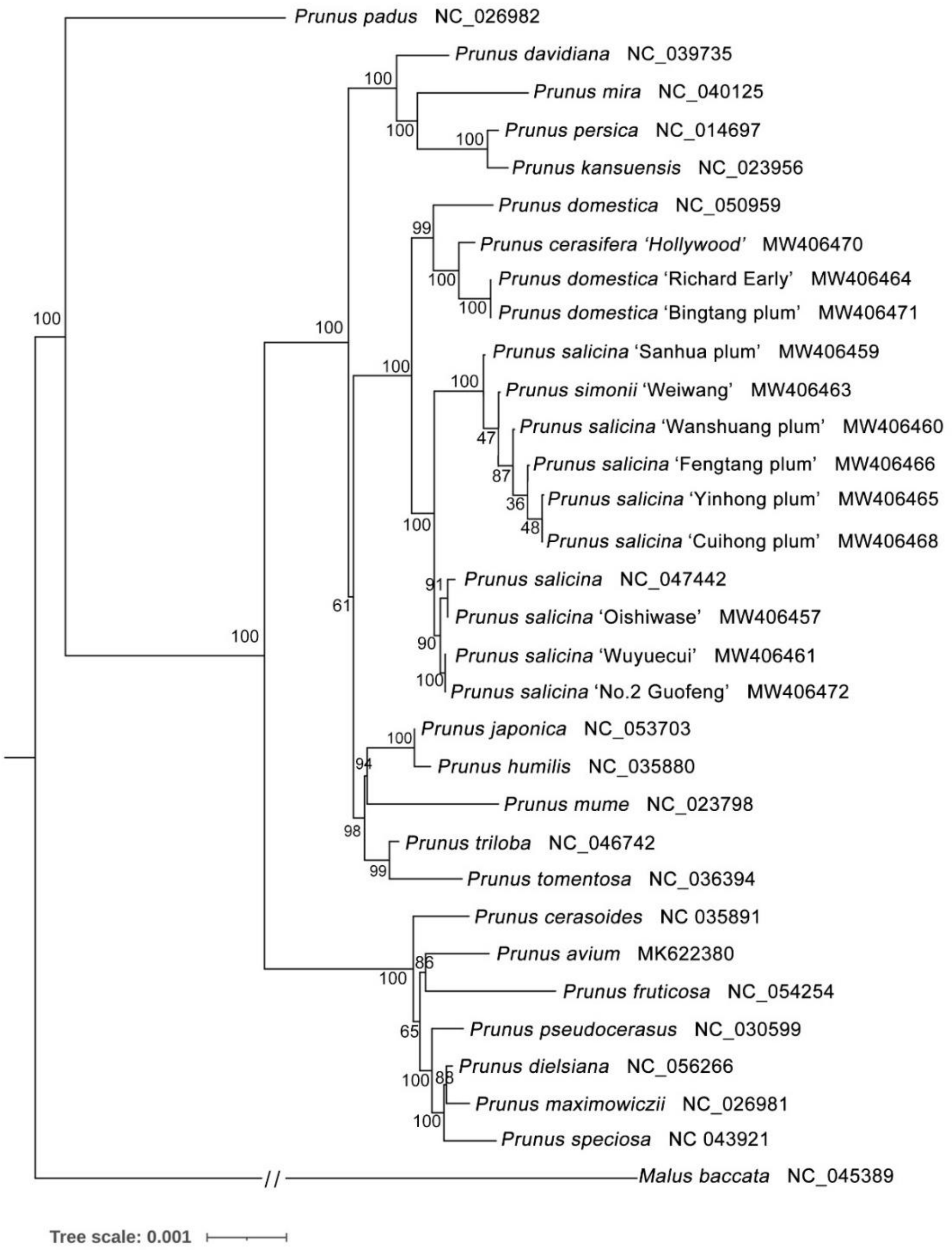

B.

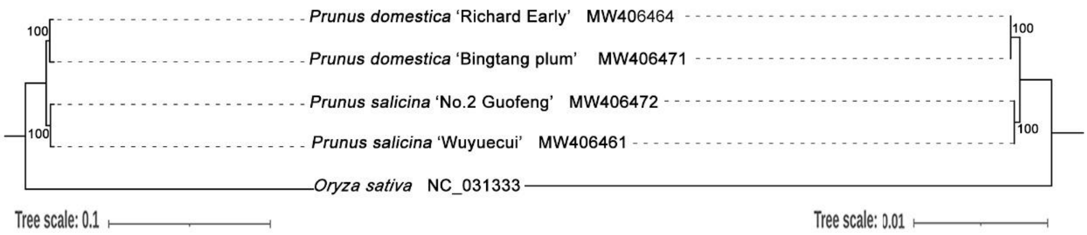

Figure S12. Phylogenetic relationships of species from *Prunus* (Rosaceae) inferred using Maximum likelihood (ML) method. A.The phylogenetic tree was constructed using 71

common protein-coding genes among the 32 plastomes. The number at the bottom of the scale, 0.001, means that the length of the branch represents the replacement frequency of bases at each site of the genome at 0.001. Bootstrap values were calculated from 1000 replicates. B. The phylogenetic tree constructed using nuclear genes among five plastomes.

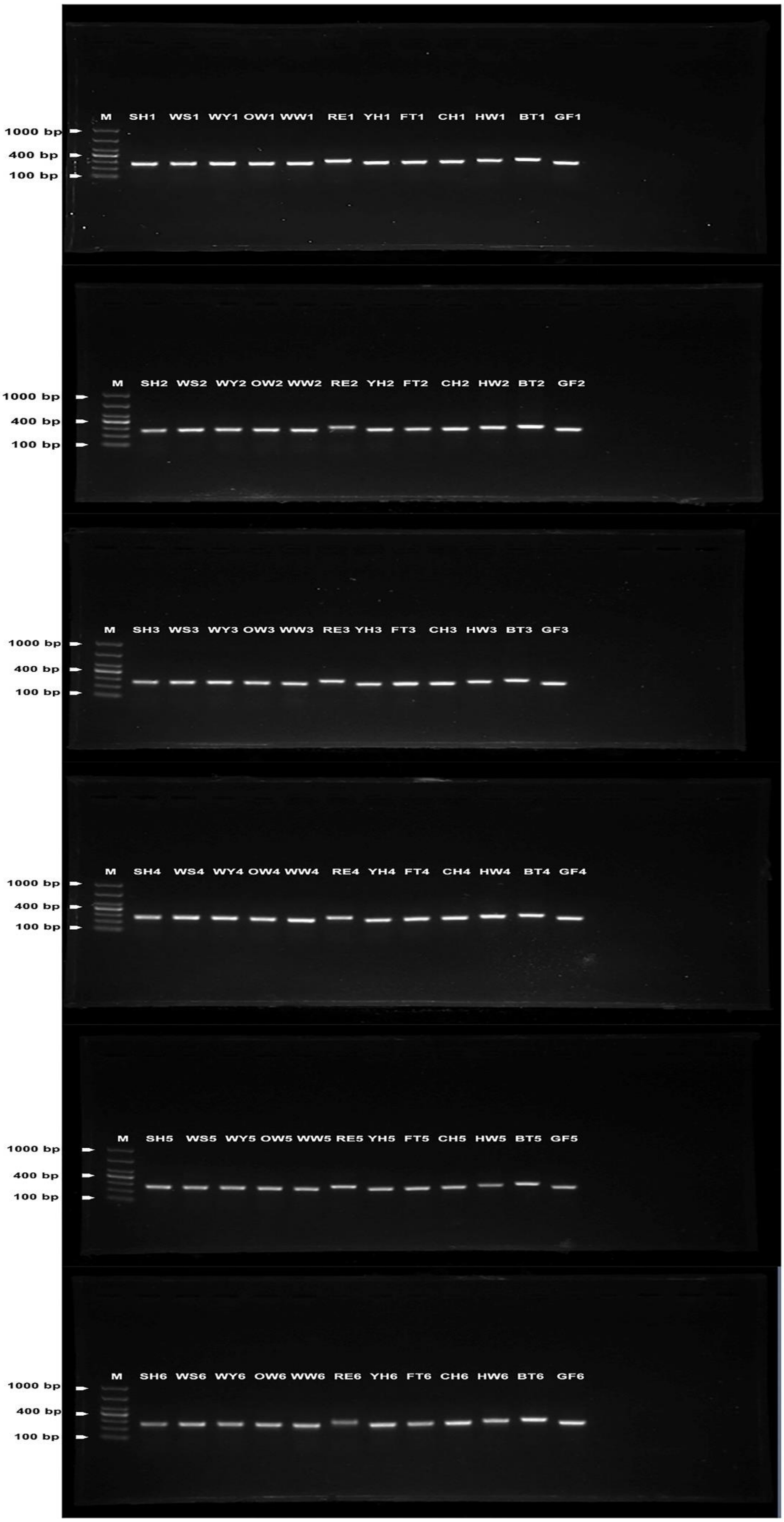
M SH1 WS1 WY1 OW1 WW1 RE1 YH1 FT1 CH1 HW1 BT1 GF1
1000 bp
400 bp
100 bp
M SH2 WS2 WY2 OW2 WW2 RE2 YH2 FT2 CH2 HW2 BT2 GF2
1000 bp
400 bp
100 bp
M SH3 WS3 WY3 OW3 WW3 RE3 YH3 FT3 CH3 HW3 BT3 GF3
1000 bp
400 bp
100 bp
M SH4 WS4 WY4 OW4 WW4 RE4 YH4 FT4 CH4 HW4 BT4 GF4
1000 bp
400 bp
100 bp
M SH5 WS5 WY5 OW5 WW5 RE5 YH5 FT5 CH5 HW5 BT5 GF5
1000 bp
400 bp
100 bp
M SH6 WS6 WY6 OW6 WW6 RE6 YH6 FT6 CH6 HW6 BT6 GF6
1000 bp
400 bp
100 bp

Figure S13. The gel electrophoresis results of the amplification of DNA barcodes using designed primer LZ01. Lane M was the marker of DL1000. Lane M was the marker of DL1000. The lanes from left to right corresponded to products amplificated from the first individual of *P. salicina* 'Sanhua plum' (SH); *P. salicina* 'Wanshuang plum' (WS); *P. salicina* 'Wuyuecui' (WY); *P. salicina* 'Oishiwase' (OW); *P. simonii* 'Weiwang' (WW); *P. domestica* 'Richard Early' (RE); *P. salicina* 'Yinhong plum' (YH); *P. salicina* 'Fengtang plum' (FT); *P. salicina* 'Cuihong plum' (CH); *P. cerasifera* 'Hollywood' (HW); *P. domestica* 'Bingtang plum' (BT) and *P. salicina* 'No.2 Guofeng' (GF). Arabic numerals represent different individuals.

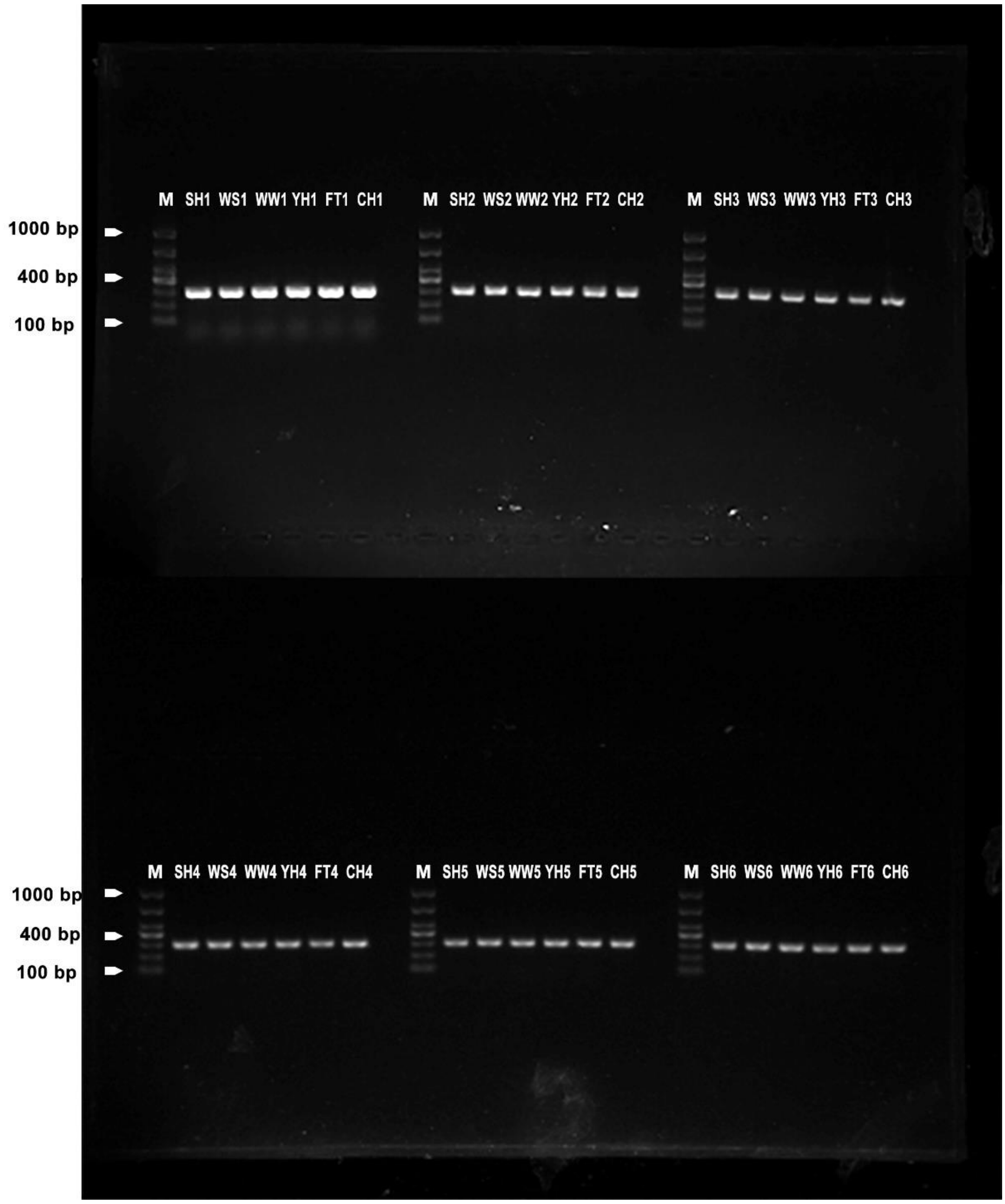

Figure S14. The gel electrophoresis results of the amplification of DNA barcodes using designed primer LZ02. Lane M was the marker of DL1000. Lane M was the marker of DL1000. The lanes from left to right corresponded to products amplificated from the first individual of *P. salicina* 'Sanhua plum' (SH); *P. salicina* 'Wanshuang plum' (WS); *P. simonii* 'Weiwang' (WW); *P. salicina* 'Yinhong plum' (YH); *P. salicina* 'Fengtang plum' (FT) and *P. salicina* 'Cuihong plum' (CH). Arabic numerals represent different individuals.

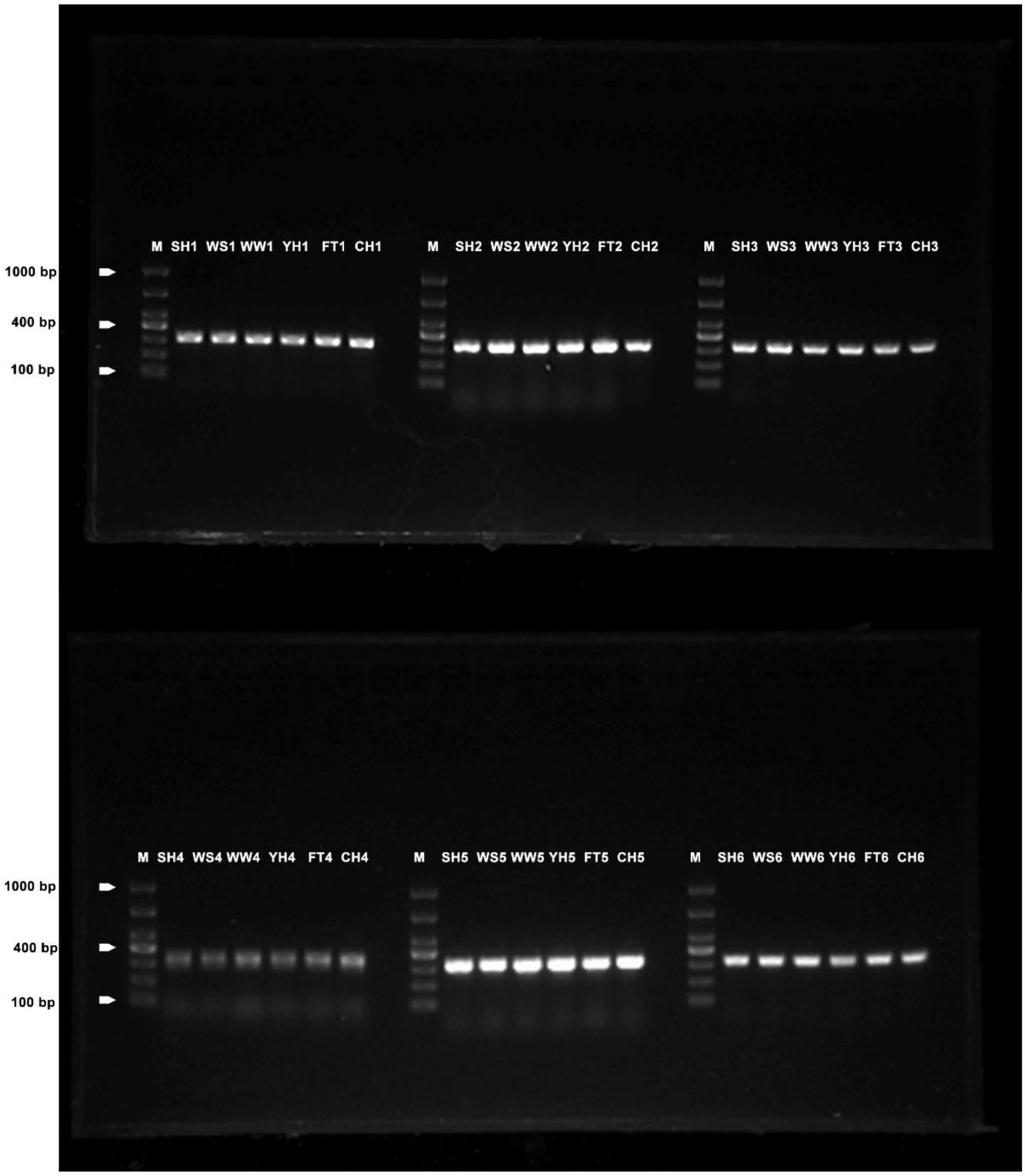

Figure S15. The gel electrophoresis results of the amplification of DNA barcodes using designed primer LZ03. Lane M was the marker of DL1000. Lane M was the marker of DL1000. The lanes from left to right corresponded to products amplificated from the first individual of *P. salicina* 'Sanhua plum' (SH); *P. salicina* 'Wanshuang plum' (WS); *P. simonii* 'Weiwang' (WW); *P. salicina* 'Yinhong plum' (YH); *P. salicina* 'Fengtang plum' (FT) and *P. salicina* 'Cuihong plum' (CH). Arabic numerals represent different individuals.

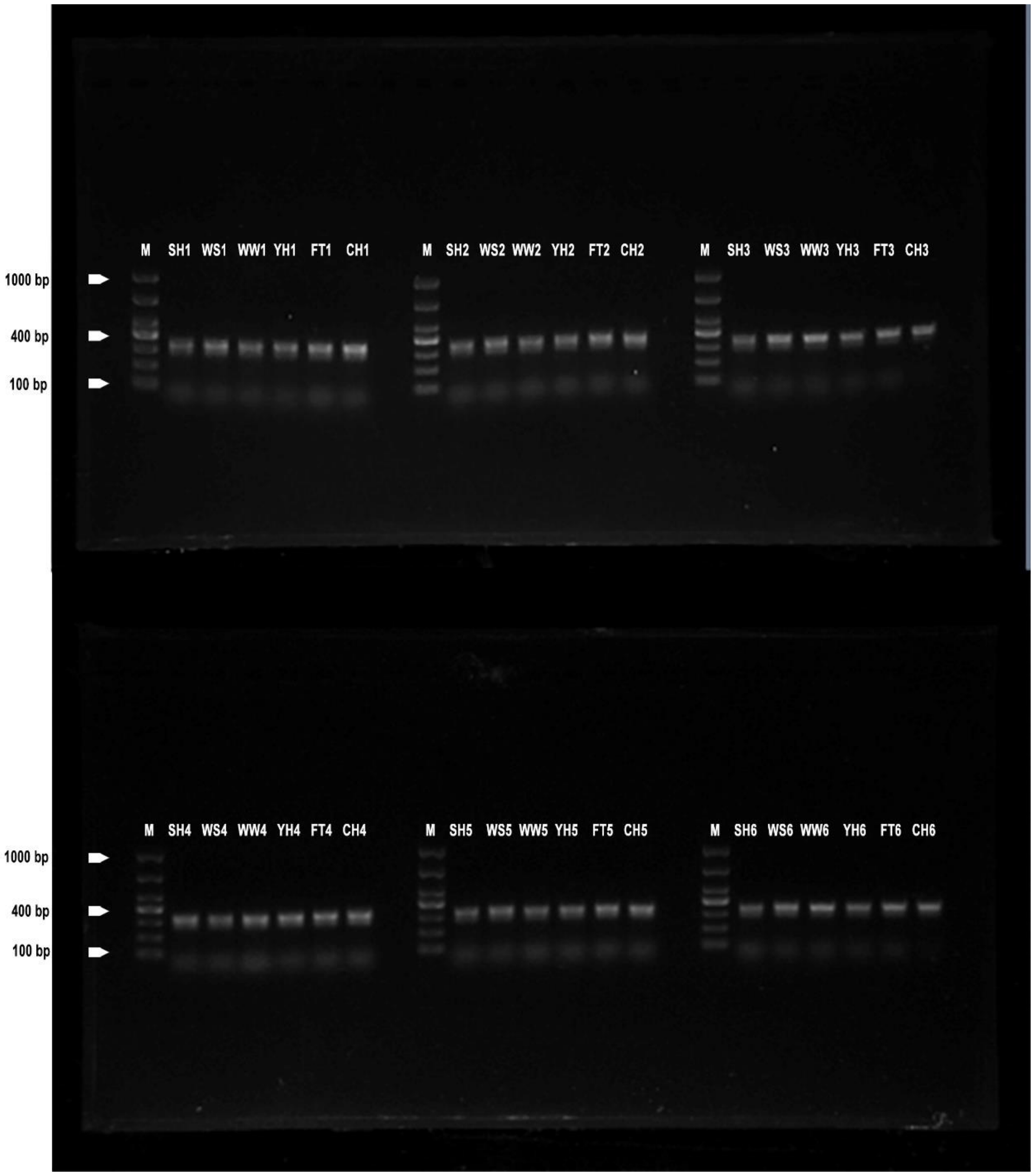

Figure S16. The gel electrophoresis results of the amplification of DNA barcodes using designed primer LZ04. Lane M was the marker of DL1000. Lane M was the marker of DL1000. The lanes from left to right corresponded to products amplificated from the first individual of *P. salicina* 'Sanhua plum' (SH); *P. salicina* 'Wanshuang plum' (WS); *P. simonii* 'Weiwang' (WW); *P. salicina* 'Yinhong plum' (YH); *P. salicina* 'Fengtang plum' (FT) and *P. salicina* 'Cuihong plum' (CH). Arabic numerals represent different individuals.

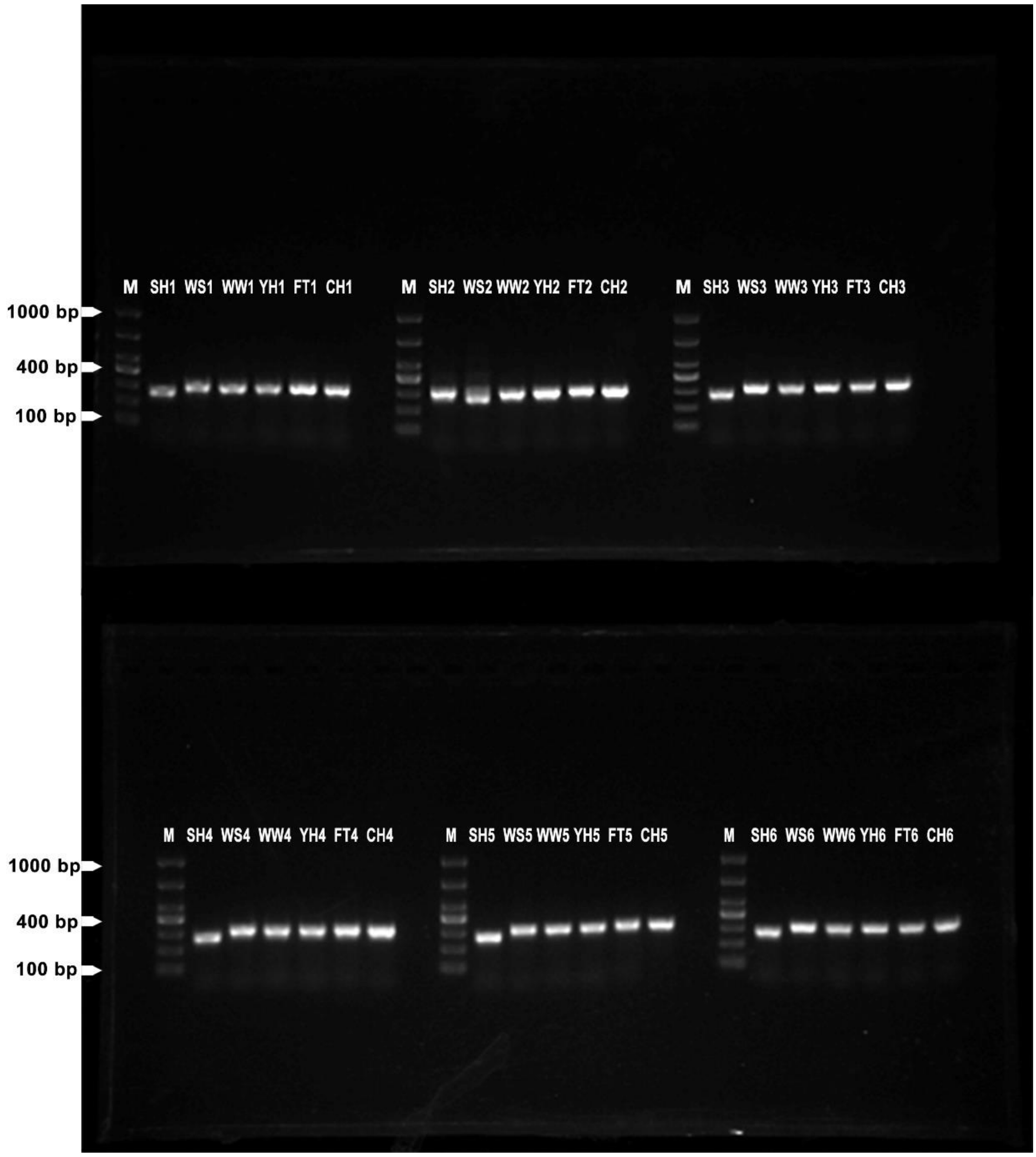

Figure S17. The gel electrophoresis results of the amplification of DNA barcodes using designed primer LZ05. Lane M was the marker of DL1000. Lane M was the marker of DL1000. The lanes from left to right corresponded to products amplificated from the first individual of *P. salicina* 'Sanhua plum' (SH); *P. salicina* 'Wanshuang plum' (WS); *P. simonii* 'Weiwang' (WW); *P. salicina* 'Yinhong plum' (YH); *P. salicina* 'Fengtang plum' (FT) and *P. salicina* 'Cuihong plum' (CH). Arabic numerals represent different individuals.

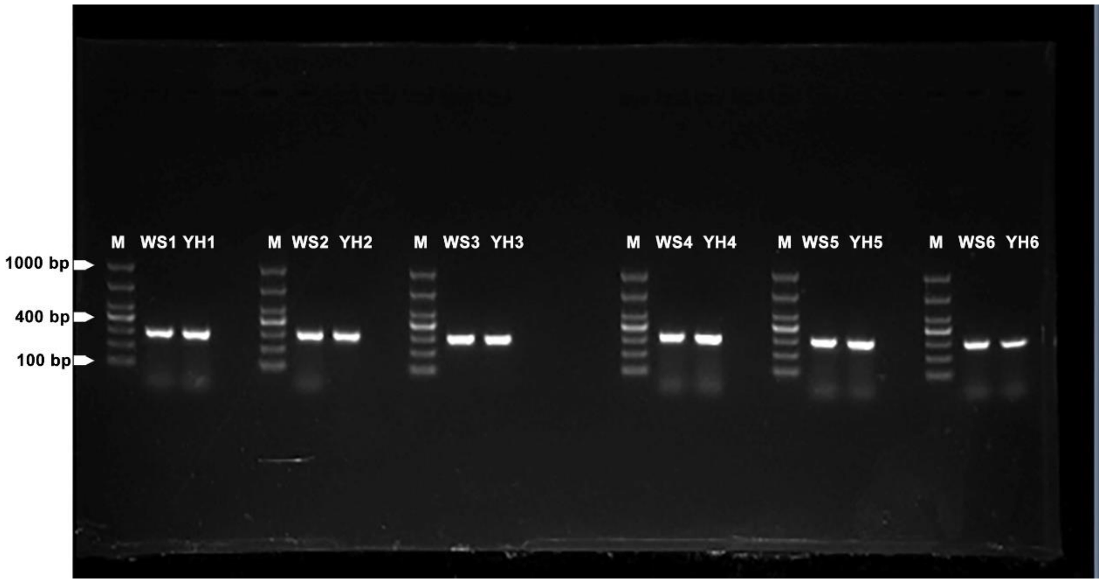

Figure S18. The gel electrophoresis results of the amplification of DNA barcodes using designed primer LZ06. Lane M was the marker of DL1000. Lane M was the marker of DL1000. The lanes from left to right corresponded to products amplificated from the first individual of *P. salicina* 'Wanshuang plum' (WS) and *P. salicina* 'Yinhong plum' (YH). Arabic numerals represent different individuals.

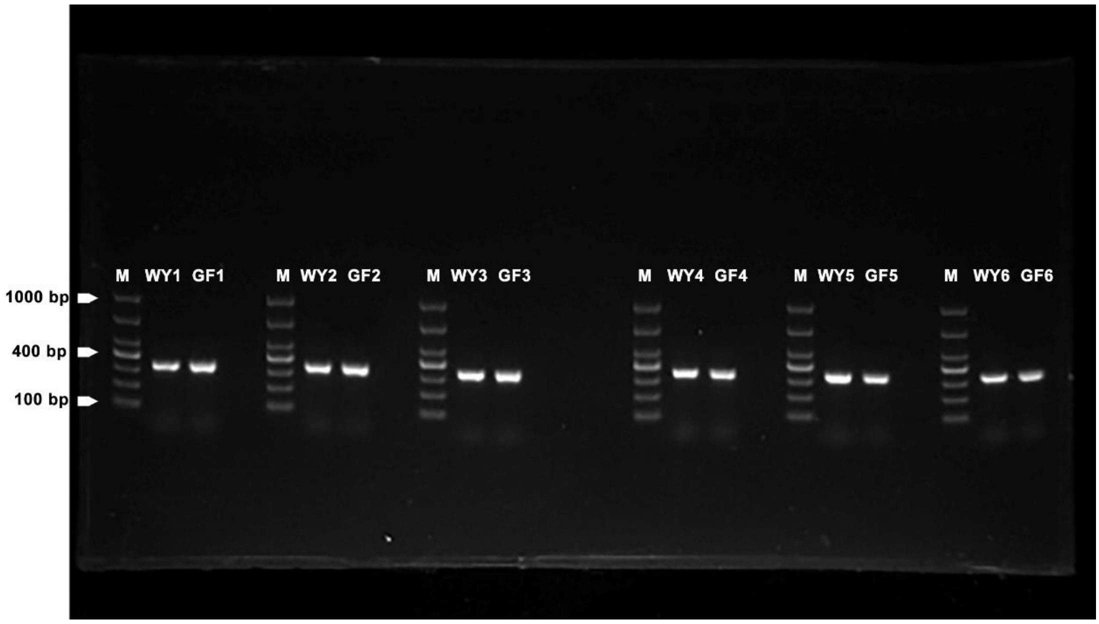

Figure S19. The gel electrophoresis results of the amplification of DNA barcodes using designed primer LZ07. Lane M was the marker of DL1000. Lane M was the marker of DL1000. The lanes from left to right corresponded to products amplificated from the first individual of *P. salicina* 'Wuyuecui' (WY) and *P. salicina* 'No.2 Guofeng' (GF). Arabic numerals represent different individuals.

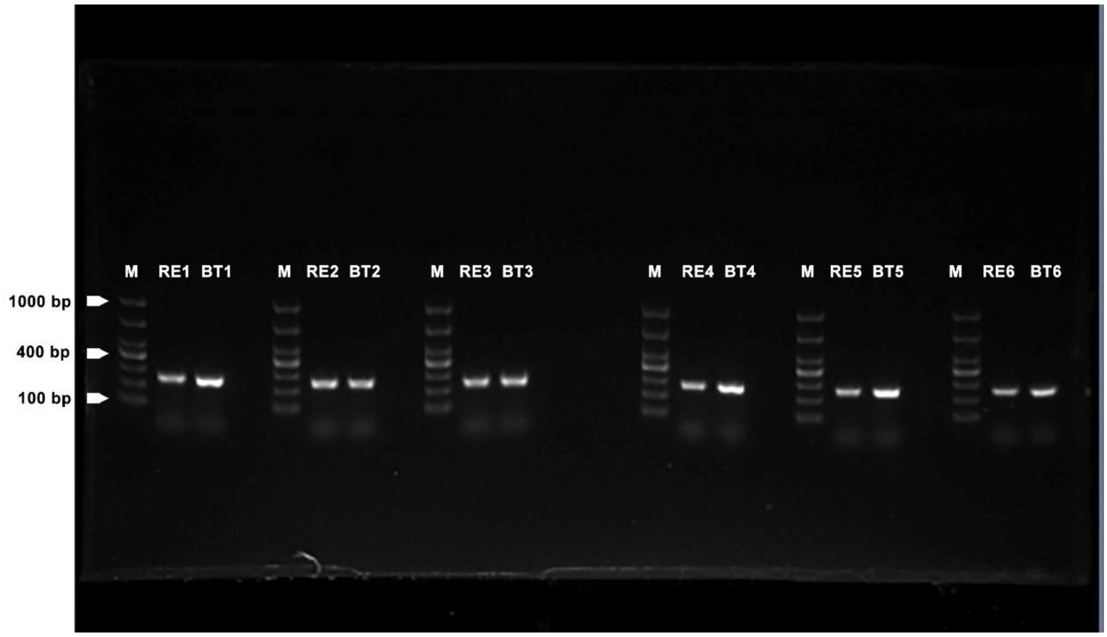

Figure S20. The gel electrophoresis results of the amplification of DNA barcodes using designed primer LZ08. Lane M was the marker of DL1000. Lane M was the marker of DL1000. The lanes from left to right corresponded to products amplificated from the first individual of *P. domestica* 'Richard Early' (RE) and *P. domestica* 'Bingtang plum' (BT). Arabic numerals represent different individuals.

```
SH1_LZ01 : ---gaatctttg-taagatgtataagattttgatagataca-t---act :  42
WS1_LZ01 : -------cttttg-aaagatgtataagattttgatagataca-t---act :  38
WY1_LZ01 : ----aatccattg-ttagatgtataagattttgatagataca-t---act :  41
OW1_LZ01 : ------caatttg-ttagatgtataagattttgatagataca-t---act :  39
WW1_LZ01 : ----tatcctttt-gaagatgtataagattttgatagataca-t---act :  41
RE1_LZ01 : -----acccattg-aaagatgtataagattttgatagataca-t---act :  40
YH1_LZ01 : --------aattt-gtagatgtataagattttgatagataca-t---act :  37
FT1_LZ01 : ------cctttg-aaagatgtataagattttgatagataca-t---act :  39
CH1_LZ01 : -----------gg-caagatgtataagattttgatagataca-t---act :  34
HW1_LZ01 : ------------ggatagatgtataagattttgatagataca-t---act :  34
BT1_LZ01 : ----gatcaattg-taagatgtataagattttgatagataca-t---act :  41
GF1_LZ01 : -------tttttg-aaagatgtataagattttgatagataca-t---act :  38
SH2_LZ01 : ------catttg-taagatgtataagattttgatagataca-t---act :  39
WS2_LZ01 : -----------tg-aaagatgtataagattttgatagataca-t---act :  34
WY2_LZ01 : ----------tggcatcgatgtataagattttgatagataca-t---act :  36
OW2_LZ01 : --------ctttg-taagatgtataagattttgatagataca-t---act :  37
WW2_LZ01 : ------------g-gaagatgtataagattttgatagataca-t---act :  33
RE2_LZ01 : ------tcatttg-ttagatgtataagattttgatagataca-t---act :  39
YH2_LZ01 : -------cttttg-taagatgtataagattttgatagataca-t---act :  38
FT2_LZ01 : ------catttg-ttcgatgtataagattttgatagataca-t---act :  39
CH2_LZ01 : -------tttttg-taagatgtataagattttgatagataca-t---act :  38
HW2_LZ01 : -----------tgtttagatgtataagattttgatagataca-t---act :  35
BT2_LZ01 : -------ctattg-aaagatgtataagattttgatagataca-t---act :  38
GF2_LZ01 : ----gatcaattg-taagatgtataagattttgatagataca-t---act :  41
SH3_LZ01 : ttaatcttttggt-tttgatgtataagattttgatagataca-t---act :  45
WS3_LZ01 : ---aatcctttg-tttgatgtataagattttgatagataca-t---act :  42
WY3_LZ01 : -caatattttgt-atagatgtataagattttgatagataca-t---act :  44
OW3_LZ01 : ---tggtctattg-caagatgtataagattttgatagataca-t---act :  42
WW3_LZ01 : ----gatctttt-gaagatgtataagattttgatagataca-t---act :  41
RE3_LZ01 : -----gatcttg-ttagatgtataagattttgatagataca-t---act :  40
YH3_LZ01 : ---aatcctttga-atagatgtataagattttgatagataca-t---act :  42
FT3_LZ01 : -gaacccatttgt-aaagatgtataagattttgatagataca-t---act :  44
CH3_LZ01 : -aaatcttttgt-ttagatgtataagattttgatagataca-t---act :  44
HW3_LZ01 : ---aattccattt-gtagatgtataagattttgatagataca-t---act :  42
BT3_LZ01 : ---gatcatttg-taagatgtataagattttgatagataca-t---act :  42
GF3_LZ01 : --gatccatttg-ttagatgtataagattttgatagataca-t---act :  43
```

```
SH4_LZ01 : ----gtggtgttg-taagatgtatagatttttgatagatacatt---act :  42
WS4_LZ01 : -----aggtattc-gaagatgtatagatttttgatagataca-t---act :  40
WY4_LZ01 : -----atatatcc-gcagatgtatagatttttgatagataca-t---act :  40
OW4_LZ01 : ---gtgaaagctg-ttcgatgtatcgatttttgatagatacatt---act :  43
WW4_LZ01 : ----atggcagct-gttgatgtatcgatttttgatagataca-t---tcc :  41
RE4_LZ01 : -----aggccatt-gttgatgtatagatttttgatagataca-t---act :  40
YH4_LZ01 : ---aggaattttg-atagatgtatagatttttgatagataca-t---act :  42
FT4_LZ01 : ----gtacagttg-tatgatgtatcgatttttgatagataca-ttacttt :  44
CH4_LZ01 : ----atgccgctg-tatgatgtatcgatttttgatagataca-t---tcc :  41
HW4_LZ01 : ----agactctcc-gaagatgtatagatttttgatagataca-t---act :  41
BT4_LZ01 : ----attgaagct-gttgatgtatagatttttgatagataca-t---cct :  41
GF4_LZ01 : ----gtggtattc-gaagatgtatagatttttgatagataca-t---act :  41
SH5_LZ01 : ----aggtcgaac-gcagatgtatagatttttgatagataca-t---act :  41
WS5_LZ01 : ----gtaccgatt-gaagatgtatagatttttgatagataca-t---act :  41
WY5_LZ01 : ---attggtagac-gaagatgtatagatttttgatagataca-t---act :  42
OW5_LZ01 : ----agagtcgat-gatgatgtatagatttttgatagataca-t---act :  41
WW5_LZ01 : ----atgaccgat-gaagatgtatagatttttgatagataca-t---act :  41
RE5_LZ01 : ----ggggatcat-gaagatgtatagatttttgatagataca-t---act :  41
YH5_LZ01 : ----agaactatg-atagatgtatagatttttgatagataca-t---act :  41
FT5_LZ01 : ----gtaaccgat-gaagatgtatagatttttgatagataca-t---act :  41
CH5_LZ01 : ---gtgatatatt-gaagatgtatagatttttgatagataca-t---act :  42
HW5_LZ01 : ----gtttagatt-gaagatgtatagatttttgatagataca-t---act :  41
BT5_LZ01 : -----tgaacgat-gaagatgtatagatttttgatagataca-t---act :  40
GF5_LZ01 : ----atatcgatg-ctagatgtatagatttttgatagataca-t---act :  41
SH6_LZ01 : -----gaatcatt-gaagatgtatagatttttgatagataca-t---act :  40
WS6_LZ01 : -----gggtcatt-gaagatgtatagatttttgatagataca-t---act :  40
WY6_LZ01 : --acgggtctttt-gaagatgtatagatttttgatagataca-t---act :  43
OW6_LZ01 : --ggggtctattg-taagatgtatagatttttgatagataca-t---act :  43
WW6_LZ01 : ---gggatcattg-taagatgtatagatttttgatagataca-t---act :  42
RE6_LZ01 : ---ggatcttttg-taagatgtatagatttttgatagataca-t---act :  42
YH6_LZ01 : ---ggatctttgt-atagatgtatagatttttgatagataca-t---act :  42
FT6_LZ01 : ---gggatctttg-taagatgtatagatttttgatagataca-t---act :  42
CH6_LZ01 : ----agatctttg-taagatgtatagatttttgatagataca-t---act :  41
HW6_LZ01 : --gggatcttttg-ttagatgtatagatttttgatagataca-t---act :  43
BT6_LZ01 : ---agatcattg-taagatgtatagatttttgatagataca-t---act :  42
GF6_LZ01 : --aggatctattg-taagatgtatagatttttgatagataca-t---act :  43
```

```
SH1_LZ01 : tat--a-t-----agatatacaagatcttaaatacaaaaatataagacga : 84
WS1_LZ01 : tat--a-t-----agatatacaagatcttaaatacaaaaatataagacga : 80
WY1_LZ01 : tat--a-t-----agatatacaagatcttaaatacaaaaatataagacga : 83
OW1_LZ01 : tat--a-t-----agatatacaagatcttaaatacaaaaatataagacga : 81
WW1_LZ01 : tat--a-t-----agatatacaagatcttaaatacaaaaatataagacga : 83
RE1_LZ01 : tat--a-t-----agatatacaagatcttaaatacaaaaatataagacga : 82
YH1_LZ01 : tat--a-t-----agatatacaagatcttaaatacaaaaatataagacga : 79
FT1_LZ01 : tat--a-t-----agatatacaagatcttaaatacaaaaatataagacga : 81
CH1_LZ01 : tat--a-t-----agatatacaagatcttaaatacaaaaatataagacga : 76
HW1_LZ01 : tat--a-t-----agatatacaagatcttaaatacaaaaatataagacga : 76
BT1_LZ01 : tat--a-t-----agatatacaagatcttaaatacaaaaatataagacga : 83
GF1_LZ01 : tat--a-t-----agatatacaagatcttaaatacaaaaatataagacga : 80
SH2_LZ01 : tat--a-t-----agatatacaagatcttaaatacaaaaatataagacga : 81
WS2_LZ01 : tat--a-t-----agatatacaagatcttaaatacaaaaatataagacga : 76
WY2_LZ01 : tat--a-t-----agatatacaagatcttaaatacaaaaatataagacga : 78
OW2_LZ01 : tat--a-t-----agatatacaagatcttaaatacaaaaatataagacga : 79
WW2_LZ01 : tat--a-t-----agatatacaagatcttaaatacaaaaatataagacga : 75
RE2_LZ01 : tat--a-t-----agatatacaagatcttaaatacaaaaatataagacga : 81
YH2_LZ01 : tat--a-t-----agatatacaagatcttaaatacaaaaatataagacga : 80
FT2_LZ01 : tat--a-t-----agatatacaagatcttaaatacaaaaatataagacga : 81
CH2_LZ01 : tat--a-t-----agatatacaagatcttaaatacaaaaatataagacga : 80
HW2_LZ01 : tat--a-t-----agatatacaagatcttaaatacaaaaatataagacga : 77
BT2_LZ01 : tat--a-t-----agatatacaagatcttaaatacaaaaatataagacga : 80
GF2_LZ01 : tat--a-t-----agatatacaagatcttaaatacaaaaatataagacga : 83
SH3_LZ01 : tat--a-t-----agatatacaagatcttaaatacaaaaatataagacga : 87
WS3_LZ01 : tat--a-t-----agatatacaagatcttaaatacaaaaatataagacga : 84
WY3_LZ01 : tat--a-t-----agatatacaagatcttaaatacaaaaatataagacga : 86
OW3_LZ01 : tat--a-t-----agatatacaagatcttaaatacaaaaatataagacga : 84
WW3_LZ01 : tat--a-t-----agatatacaagatcttaaatacaaaaatataagacga : 83
RE3_LZ01 : tat--a-t-----agatatacaagatcttaaatacaaaaatataagacga : 82
YH3_LZ01 : tat--a-t-----agatatacaagatcttaaatacaaaaatataagacga : 84
FT3_LZ01 : tat--a-t-----agatatacaagatcttaaatacaaaaatataagacga : 86
CH3_LZ01 : tat--a-t-----agatatacaagatcttaaatacaaaaatataagacga : 86
HW3_LZ01 : tat--a-t-----agatatacaagatcttaaatacaaaaatataagacga : 84
BT3_LZ01 : tat--a-t-----agatatacaagatcttaaatacaaaaatataagacga : 84
GF3_LZ01 : tat--a-t-----agatatacaagatcttaaatacaaaaatataagacga : 85
```

```
SH4_LZ01 : tat--a-t-----agatatacaagatcttaaatacaaaaatataagacga :  84
WS4_LZ01 : tat--a-t-----agatatacaagatcttaaatacaaaaatataagacga :  82
WY4_LZ01 : tat--a-t-----agatatacaagatcttaaatacaaaaatataagacga :  82
OW4_LZ01 : tat--ctt-----cgatatacaagatcttaaatacaaaaatataagacga :  86
WW4_LZ01 : -tt--a-ttcttcgatattacaagatcttaaatacaaaaatataagacga :  87
RE4_LZ01 : tattct-t-----agatatacaagatcttaaatacaaaaatataagacga :  84
YH4_LZ01 : tat--a-t-----agatatacaagatcttaaatacaaaaatataagacga :  84
FT4_LZ01 : att--a-t-----agatatacaagatcttaaatacaaaaatataagacga :  86
CH4_LZ01 : ttt--a-ttcttcgatattccaagatcttaaatacaaaaatataagacga :  88
HW4_LZ01 : tat--a-t-----agatatacaagatcttaaatacaaaaatataagacga :  83
BT4_LZ01 : tat--a-t-----agatatacaagatcttaaatacaaaaatataagacga :  83
GF4_LZ01 : tat--a-t-----agatatacaagatcttaaatacaaaaatataagacga :  83
SH5_LZ01 : tat--a-t-----agatatacaagatcttaaatacaaaaatataagacga :  83
WS5_LZ01 : tat--a-t-----agatatacaagatcttaaatacaaaaatataagacga :  83
WY5_LZ01 : tat--a-t-----agatatacaagatcttaaatacaaaaatataagacga :  84
OW5_LZ01 : tat--a-t-----agatatacaagatcttaaatacaaaaatataagacga :  83
WW5_LZ01 : tat--a-t-----agatatacaagatcttaaatacaaaaatataagacga :  83
RE5_LZ01 : tat--a-t-----agatatacaagatcttaaatacaaaaatataagacga :  83
YH5_LZ01 : tat--a-t-----agatatacaagatcttaaatacaaaaatataagacga :  83
FT5_LZ01 : tat--a-t-----agatatacaagatcttaaatacaaaaatataagacga :  83
CH5_LZ01 : tat--a-t-----agatatacaagatcttaaatacaaaaatataagacga :  84
HW5_LZ01 : tat--a-t-----agatatacaagatcttaaatacaaaaatataagacga :  83
BT5_LZ01 : tat--a-t-----agatatacaagatcttaaatacaaaaatataagacga :  82
GF5_LZ01 : tat--a-t-----agatatacaagatcttaaatacaaaaatataagacga :  83
SH6_LZ01 : tat--a-t-----agatatacaagatcttaaatacaaaaatataagacga :  82
WS6_LZ01 : tat--a-t-----agatatacaagatcttaaatacaaaaatataagacga :  82
WY6_LZ01 : tat--a-t-----agatatacaagatcttaaatacaaaaatataagacga :  85
OW6_LZ01 : tat--a-t-----agatatacaagatcttaaatacaaaaatataagacga :  85
WW6_LZ01 : tat--a-t-----agatatacaagatcttaaatacaaaaatataagacga :  84
RE6_LZ01 : tat--a-t-----agatatacaagatcttaaatacaaaaatataagacga :  84
YH6_LZ01 : tat--a-t-----agatatacaagatcttaaatacaaaaatataagacga :  84
FT6_LZ01 : tat--a-t-----agatatacaagatcttaaatacaaaaatataagacga :  84
CH6_LZ01 : tat--a-t-----agatatacaagatcttaaatacaaaaatataagacga :  83
HW6_LZ01 : tat--a-t-----agatatacaagatcttaaatacaaaaatataagacga :  85
BT6_LZ01 : tat--a-t-----agatatacaagatcttaaatacaaaaatataagacga :  84
GF6_LZ01 : tat--a-t-----agatatacaagatcttaaatacaaaaatataagacga :  85
```

```
SH1_LZ01 : aacaactaattctttta--tt-gttgggttggatccacaattaatcctat : 131
WS1_LZ01 : aacaactaattctttta--tt-gttgggttggatccacaattaatcctat : 127
WY1_LZ01 : aacaactaattctttta--tt-gttgggttggatccacaattaatcctat : 130
OW1_LZ01 : aacaactaattctttta--tt-gttgggttggatccacaattaatcctat : 128
WW1_LZ01 : aacaactaattctttta--tt-gttgggttggatccacaattaatcctat : 130
RE1_LZ01 : aacaactaattctttta--tt-gttgggttggatccacaattaatcctat : 129
YH1_LZ01 : aacaactaattctttta--tt-gttgggttggatccacaattaatcctat : 126
FT1_LZ01 : aacaactaattctttta--tt-gttgggttggatccacaattaatcctat : 128
CH1_LZ01 : aacaactaattctttta--tt-gttgggttggatccacaattaatcctat : 123
HW1_LZ01 : aacaactaattctttta--tt-gttgggttggatccacaattaatcctat : 123
BT1_LZ01 : aacaactaattctttta--tt-gttgggttggatccacaattaatcctat : 130
GF1_LZ01 : aacaactaattctttta--tt-gttgggttggatccacaattaatcctat : 127
SH2_LZ01 : aacaactaattctttta--tt-gttgggttggatccacaattaatcctat : 128
WS2_LZ01 : aacaactaattctttta--tt-gttgggttggatccacaattaatcctat : 123
WY2_LZ01 : aacaactaattctttta--tt-gttgggttggatccacaattaatcctat : 125
OW2_LZ01 : aacaactaattctttta--tt-gttgggttggatccacaattaatcctat : 126
WW2_LZ01 : aacaactaattctttta--tt-gttgggttggatccacaattaatcctat : 122
RE2_LZ01 : aacaactaattctttta--tt-gttgggttggatccacaattaatcctat : 128
YH2_LZ01 : aacaactaattctttta--tt-gttgggttggatccacaattaatcctat : 127
FT2_LZ01 : aacaactaattctttta--tt-gttgggttggatccacaattaatcctat : 128
CH2_LZ01 : aacaactaattctttta--tt-gttgggttggatccacaattaatcctat : 127
HW2_LZ01 : aacaactaattctttta--tt-gttgggttggatccacaattaatcctat : 124
BT2_LZ01 : aacaactaattctttta--tt-gttgggttggatccacaattaatcctat : 127
GF2_LZ01 : aacaactaattctttta--tt-gttgggttggatccacaattaatcctat : 130
SH3_LZ01 : aacaactaattctttta--tt-gttgggttggatccacaattaatcctat : 134
WS3_LZ01 : aacaactaattctttta--tt-gttgggttggatccacaattaatcctat : 131
WY3_LZ01 : aacaactaattctttta--tt-gttgggttggatccacaattaatcctat : 133
OW3_LZ01 : aacaactaattctttta--tt-gttgggttggatccacaattaatcctat : 131
WW3_LZ01 : aacaactaattctttta--tt-gttgggttggatccacaattaatcctat : 130
RE3_LZ01 : aacaactaattctttta--tt-gttgggttggatccacaattaatcctat : 129
YH3_LZ01 : aacaactaattctttta--tt-gttgggttggatccacaattaatcctat : 131
FT3_LZ01 : aacaactaattctttta--tt-gttgggttggatccacaattaatcctat : 133
CH3_LZ01 : aacaactaattctttta--tt-gttgggttggatccacaattaatcctat : 133
HW3_LZ01 : aacaactaattctttta--tt-gttgggttggatccacaattaatcctat : 131
BT3_LZ01 : aacaactaattctttta--tt-gttgggttggatccacaattaatcctat : 131
GF3_LZ01 : aacaactaattctttta--tt-gttgggttggatccacaattaatcctat : 132
```

```
SH4_LZ01 : aacaactaattctttta--tt-gttgggttggatccacaattaatcctat : 131
WS4_LZ01 : aacaactaattctttta--tt-gttgggttggatccacaattaatcctat : 129
WY4_LZ01 : aacaactaattctttta--tt-gttgggttggatccacaattaatcctat : 129
OW4_LZ01 : aacaactaattctttta--tt-gttgggttggatccacaattaatcctat : 133
WW4_LZ01 : aacaactaattctttta--tt-gttgggttggatccacaattaatcctat : 134
RE4_LZ01 : aacaactaattctttta--tt-gttgggttggatccacaattaatcctat : 131
YH4_LZ01 : aacaactaattctttta--tt-gttgggttggatccacaattaatcctat : 131
FT4_LZ01 : aacaactaattctttta--ttcgttgggttggatccacaattaatcctat : 134
CH4_LZ01 : aacaactaattctttta--tt-gttgggttggatccacaattaatcctat : 135
HW4_LZ01 : aacaactaattctttta--tt-gttgggttggatccacaattaatcctat : 130
BT4_LZ01 : aacaactaattctttta--tt-gttgggttggatccacaattaatcctat : 130
GF4_LZ01 : aacaactaattctttta--tt-gttgggttggatccacaattaatcctat : 130
SH5_LZ01 : aacaactaattctttta--tt-gttgggttggatccacaattaatcctat : 130
WS5_LZ01 : aacaactaattctttta--tt-gttgggttggatccacaattaatcctat : 130
WY5_LZ01 : aacaactaattctttta--tt-gttgggttggatccacaattaatcctat : 131
OW5_LZ01 : aacaactaattctttta--tt-gttgggttggatccacaattaatcctat : 130
WW5_LZ01 : aacaactaattctttta--tt-gttgggttggatccacaattaatcctat : 130
RE5_LZ01 : aacaactaattctttta--tt-gttgggttggatccacaattaatcctat : 130
YH5_LZ01 : aacaactaattctttta--tt-gttgggttggatccacaattaatcctat : 130
FT5_LZ01 : aacaactaattctttta--tt-gttgggttggatccacaattaatcctat : 130
CH5_LZ01 : aacaactaattctttta--tt-gttgggttggatccacaattaatcctat : 131
HW5_LZ01 : aacaactaattctttta--tt-gttgggttggatccacaattaatcctat : 130
BT5_LZ01 : aacaactaattctttta--tt-gttgggttggatccacaattaatcctat : 129
GF5_LZ01 : aacaactaattctttta--tt-gttgggttggatccacaattaatcctat : 130
SH6_LZ01 : aacaactaattctttta--tt-gttgggttggatccacaattaatcctat : 129
WS6_LZ01 : aacaactaattctttta--tt-gttgggttggatccacaattaatcctat : 129
WY6_LZ01 : aacaactaattctttta--tt-gttgggttggatccacaattaatcctat : 132
OW6_LZ01 : aacaactaattctttta--tt-gttgggttggatccacaattaatcctat : 132
WW6_LZ01 : aacaactaattctttta--tt-gttgggttggatccacaattaatcctat : 131
RE6_LZ01 : aacaactaattctttta--tt-gttgggttggatccacaattaatcctat : 131
YH6_LZ01 : aacaactaattctttta--tt-gttgggttggatccacaattaatcctat : 131
FT6_LZ01 : aacaactaattctttta--tt-gttgggttggatccacaattaatcctat : 131
CH6_LZ01 : aacaactaattctttta--tt-gttgggttggatccacaattaatcctat : 130
HW6_LZ01 : aacaactaattctttta--tt-gttgggttggatccacaattaatcctat : 132
BT6_LZ01 : aacaactaattctttta--tt-gttgggttggatccacaattaatcctat : 131
GF6_LZ01 : aacaactaattctttta--tt-gttgggttggatccacaattaatcctat : 132
```

```
SH1_LZ01 : ggatcctt------------------aggattggcgtattcttataata- : 162
WS1_LZ01 : ggatcctt------------------aggattggcgtattcttataata- : 158
WY1_LZ01 : ggatcctt------------------aggattggcgtattcttataatat : 162
OW1_LZ01 : ggatcctt------------------aggattggcgtattcttataata- : 159
WW1_LZ01 : ggatcctt------------------aggattggcgtattcttataata- : 161
RE1_LZ01 : ggatccttaggattggcgtattcttaaggattggcgtattcttataata- : 178
YH1_LZ01 : ggatcctt------------------aggattggcgtattcttataata- : 157
FT1_LZ01 : ggatcctt------------------aggattggcgtattcttataata- : 159
CH1_LZ01 : ggatcctt------------------aggattggcgtattcttataata- : 154
HW1_LZ01 : ggatcctt------------------aggattggcgtattcttataata- : 154
BT1_LZ01 : ggatccttaggattggcgtattcttaaggattggcgtattcttataata- : 179
GF1_LZ01 : ggatcctt------------------aggattggcgtattcttataatat : 159
SH2_LZ01 : ggatcctt------------------aggattggcgtattcttataata- : 159
WS2_LZ01 : ggatcctt------------------aggattggcgtattcttataata- : 154
WY2_LZ01 : ggatcctt------------------aggattggcgtattcttataatat : 157
OW2_LZ01 : ggatcctt------------------aggattggcgtattcttataata- : 157
WW2_LZ01 : ggatcctt------------------aggattggcgtattcttataata- : 153
RE2_LZ01 : ggatccttaggattggcgtattcttaaggattggcgtattcttataata- : 177
YH2_LZ01 : ggatcctt------------------aggattggcgtattcttataata- : 158
FT2_LZ01 : ggatcctt------------------aggattggcgtattcttataata- : 159
CH2_LZ01 : ggatcctt------------------aggattggcgtattcttataata- : 158
HW2_LZ01 : ggatcctt------------------aggattggcgtattcttataata- : 155
BT2_LZ01 : ggatccttaggattggcgtattcttaaggattggcgtattcttataata- : 176
GF2_LZ01 : ggatcctt------------------aggattggcgtattcttataatat : 162
SH3_LZ01 : ggatcctt------------------aggattggcgtattcttataata- : 165
WS3_LZ01 : ggatcctt------------------aggattggcgtattcttataata- : 162
WY3_LZ01 : ggatcctt------------------aggattggcgtattcttataatat : 165
OW3_LZ01 : ggatcctt------------------aggattggcgtattcttataata- : 162
WW3_LZ01 : ggatcctt------------------aggattggcgtattcttataata- : 161
RE3_LZ01 : ggatccttaggattggcgtattcttaaggattggcgtattcttataata- : 178
YH3_LZ01 : ggatcctt------------------aggattggcgtattcttataata- : 162
FT3_LZ01 : ggatcctt------------------aggattggcgtattcttataata- : 164
CH3_LZ01 : ggatcctt------------------aggattggcgtattcttataata- : 164
HW3_LZ01 : ggatcctt------------------aggattggcgtattcttataata- : 162
BT3_LZ01 : ggatccttaggattggcgtattcttaaggattggcgtattcttataata- : 180
GF3_LZ01 : ggatcctt------------------aggattggcgtattcttataatat : 164
```

```
SH4_LZ01 : ggatcctt------------------aggattggcgtattcttataata- : 162
WS4_LZ01 : ggatcctt------------------aggattggcgtattcttataata- : 160
WY4_LZ01 : ggatcctt------------------aggattggcgtattcttataatat : 161
OW4_LZ01 : ggatcctt------------------aggattggcgtattcttataata- : 164
WW4_LZ01 : ggatcctt------------------aggattggcgtattcttataata- : 165
RE4_LZ01 : ggatccttaggattggcgtattcttaaggattggcgtattcttataata- : 180
YH4_LZ01 : ggatcctt------------------aggattggcgtattcttataata- : 162
FT4_LZ01 : ggatcctt------------------aggattggcgtattcttataata- : 165
CH4_LZ01 : ggatcctt------------------aggattggcgtattcttataata- : 166
HW4_LZ01 : ggatcctt------------------aggattggcgtattcttataata- : 161
BT4_LZ01 : ggatccttaggattggcgtattcttaaggattggcgtattcttataata- : 179
GF4_LZ01 : ggatcctt------------------aggattggcgtattcttataatat : 162
SH5_LZ01 : ggatcctt------------------aggattggcgtattcttataata- : 161
WS5_LZ01 : ggatcctt------------------aggattggcgtattcttataata- : 161
WY5_LZ01 : ggatcctt------------------aggattggcgtattcttataatat : 163
OW5_LZ01 : ggatcctt------------------aggattggcgtattcttataata- : 161
WW5_LZ01 : ggatcctt------------------aggattggcgtattcttataata- : 161
RE5_LZ01 : ggatccttaggattggcgtattcttaaggattggcgtattcttataata- : 179
YH5_LZ01 : ggatcctt------------------aggattggcgtattcttataata- : 161
FT5_LZ01 : ggatcctt------------------aggattggcgtattcttataata- : 161
CH5_LZ01 : ggatcctt------------------aggattggcgtattcttataata- : 162
HW5_LZ01 : ggatcctt------------------aggattggcgtattcttataata- : 161
BT5_LZ01 : ggatccttaggattggcgtattcttaaggattggcgtattcttataata- : 178
GF5_LZ01 : ggatcctt------------------aggattggcgtattcttataatat : 162
SH6_LZ01 : ggatcctt------------------aggattggcgtattcttataata- : 160
WS6_LZ01 : ggatcctt------------------aggattggcgtattcttataata- : 160
WY6_LZ01 : ggatcctt------------------aggattggcgtattcttataatat : 164
OW6_LZ01 : ggatcctt------------------aggattggcgtattcttataata- : 163
WW6_LZ01 : ggatcctt------------------aggattggcgtattcttataata- : 162
RE6_LZ01 : ggatccttaggattggcgtattcttaaggattggcgtattcttataata- : 180
YH6_LZ01 : ggatcctt------------------aggattggcgtattcttataata- : 162
FT6_LZ01 : ggatcctt------------------aggattggcgtattcttataata- : 162
CH6_LZ01 : ggatcctt------------------aggattggcgtattcttataata- : 161
HW6_LZ01 : ggatcctt------------------aggattggcgtattcttataata- : 163
BT6_LZ01 : ggatccttaggattggcgtattcttaaggattggcgtattcttataata- : 180
GF6_LZ01 : ggatcctt------------------aggattggcgtattcttataatat : 164
```

```
SH1_LZ01 : -tttttttttatattagtttagtatattctttttttatatattattatat : 211
WS1_LZ01 : -tttttttttatattagtttagtatattctttttttatatattattatat : 207
WY1_LZ01 : ttttttttttatattagtttagtatattctttttttatatattattatat : 212
OW1_LZ01 : ttttttttttatattagtttagtatattctttttttatatattattatat : 209
WW1_LZ01 : -tttttttttatattagtttagtatattctttttttatatattattatat : 210
RE1_LZ01 : -tttttttttatattagtttagtatattctttttttatatattattatat : 227
YH1_LZ01 : -tttttttttatattagtttagtatattctttttttatatattattatat : 206
FT1_LZ01 : -tttttttttatattagtttagtatattctttttttatatattattatat : 208
CH1_LZ01 : -tttttttttatattagtttagtatattctttttttatatattattatat : 203
HW1_LZ01 : -tttttttttatattagtttagtatattctttttttatatattattatat : 203
BT1_LZ01 : -tttttttttatattagtttagtatattctttttttatatattattatat : 228
GF1_LZ01 : ttttttttttatattagtttagtatattctttttttatatattattatat : 209
SH2_LZ01 : -tttttttttatattagtttagtatattctttttttatatattattatat : 208
WS2_LZ01 : -tttttttttatattagtttagtatattctttttttatatattattatat : 203
WY2_LZ01 : ttttttttttatattagtttagtatattctttttttatatattattatat : 207
OW2_LZ01 : ttttttttttatattagtttagtatattctttttttatatattattatat : 207
WW2_LZ01 : -tttttttttatattagtttagtatattctttttttatatattattatat : 202
RE2_LZ01 : -tttttttttatattagtttagtatattctttttttatatattattatat : 226
YH2_LZ01 : -tttttttttatattagtttagtatattctttttttatatattattatat : 207
FT2_LZ01 : -tttttttttatattagtttagtatattctttttttatatattattatat : 208
CH2_LZ01 : -tttttttttatattagtttagtatattctttttttatatattattatat : 207
HW2_LZ01 : -tttttttttatattagtttagtatattctttttttatatattattatat : 204
BT2_LZ01 : -tttttttttatattagtttagtatattctttttttatatattattatat : 225
GF2_LZ01 : ttttttttttatattagtttagtatattctttttttatatattattatat : 212
SH3_LZ01 : -tttttttttatattagtttagtatattctttttttatatattattatat : 214
WS3_LZ01 : -tttttttttatattagtttagtatattctttttttatatattattatat : 211
WY3_LZ01 : ttttttttttatattagtttagtatattctttttttatatattattatat : 215
OW3_LZ01 : ttttttttttatattagtttagtatattctttttttatatattattatat : 212
WW3_LZ01 : -tttttttttatattagtttagtatattctttttttatatattattatat : 210
RE3_LZ01 : -tttttttttatattagtttagtatattctttttttatatattattatat : 227
YH3_LZ01 : -tttttttttatattagtttagtatattctttttttatatattattatat : 211
FT3_LZ01 : -tttttttttatattagtttagtatattctttttttatatattattatat : 213
CH3_LZ01 : -tttttttttatattagtttagtatattctttttttatatattattatat : 213
HW3_LZ01 : -tttttttttatattagtttagtatattctttttttatatattattatat : 211
BT3_LZ01 : -tttttttttatattagtttagtatattctttttttatatattattatat : 229
GF3_LZ01 : ttttttttttatattagtttagtatattctttttttatatattattatat : 214
```

```
SH4_LZ01 : -tttttttttatattagtttagtatattcttttttttatatattattatat : 211
WS4_LZ01 : -tttttttttatattagtttagtatattcttttttttatatattattatat : 209
WY4_LZ01 : ttttttttttatattagtttagtatattcttttttttatatattattatat : 211
OW4_LZ01 : ttttttttttatattagtttagtatattcttttttttatatattattatat : 214
WW4_LZ01 : -tttttttttatattagtttagtatattcttttttttatatattattatat : 214
RE4_LZ01 : -tttttttttatattagtttagtatattcttttttttatatattattatat : 229
YH4_LZ01 : -tttttttttatattagtttagtatattcttttttttatatattattatat : 211
FT4_LZ01 : -tttttttttatattagtttagtatattcttttttttatatattattatat : 214
CH4_LZ01 : -tttttttttatattagtttagtatattcttttttttatatattattatat : 215
HW4_LZ01 : -tttttttttatattagtttagtatattcttttttttatatattattatat : 210
BT4_LZ01 : -tttttttttatattagtttagtatattcttttttttatatattattatat : 228
GF4_LZ01 : ttttttttttatattagtttagtatattcttttttttatatattattatat : 212
SH5_LZ01 : -tttttttttatattagtttagtatattcttttttttatatattattatat : 210
WS5_LZ01 : -tttttttttatattagtttagtatattcttttttttatatattattatat : 210
WY5_LZ01 : ttttttttttatattagtttagtatattcttttttttatatattattatat : 213
OW5_LZ01 : ttttttttttatattagtttagtatattcttttttttatatattattatat : 211
WW5_LZ01 : -tttttttttatattagtttagtatattcttttttttatatattattatat : 210
RE5_LZ01 : -tttttttttatattagtttagtatattcttttttttatatattattatat : 228
YH5_LZ01 : -tttttttttatattagtttagtatattcttttttttatatattattatat : 210
FT5_LZ01 : -tttttttttatattagtttagtatattcttttttttatatattattatat : 210
CH5_LZ01 : -tttttttttatattagtttagtatattcttttttttatatattattatat : 211
HW5_LZ01 : -tttttttttatattagtttagtatattcttttttttatatattattatat : 210
BT5_LZ01 : -tttttttttatattagtttagtatattcttttttttatatattattatat : 227
GF5_LZ01 : ttttttttttatattagtttagtatattcttttttttatatattattatat : 212
SH6_LZ01 : -tttttttttatattagtttagtatattcttttttttatatattattatat : 209
WS6_LZ01 : -tttttttttatattagtttagtatattcttttttttatatattattatat : 209
WY6_LZ01 : ttttttttttatattagtttagtatattcttttttttatatattattatat : 214
OW6_LZ01 : -tttttttttatattagtttagtatattcttttttttatatattattatat : 212
WW6_LZ01 : -tttttttttatattagtttagtatattcttttttttatatattattatat : 211
RE6_LZ01 : -tttttttttatattagtttagtatattcttttttttatatattattatat : 229
YH6_LZ01 : -tttttttttatattagtttagtatattcttttttttatatattattatat : 211
FT6_LZ01 : -tttttttttatattagtttagtatattcttttttttatatattattatat : 211
CH6_LZ01 : -tttttttttatattagtttagtatattcttttttttatatattattatat : 210
HW6_LZ01 : -tttttttttatattagtttagtatattcttttttttatatattattatat : 212
BT6_LZ01 : -tttttttttatattagtttagtatattcttttttttatatattattatat : 229
GF6_LZ01 : ttttttttttatattagtttagtatattcttttttttatatattattatat : 214
```

```
SH1_LZ01 : cct--------------------gatcc-tgcgtt------ : 225
WS1_LZ01 : cct--------------------gatcc-tgcgttta---- : 223
WY1_LZ01 : cct--------------------gatcc-tgcgttaaa--- : 229
OW1_LZ01 : cct--------------------gatcc-tgcgttaaa--- : 226
WW1_LZ01 : cct--------------------gatcc-tgcgtttaa--- : 227
RE1_LZ01 : ttttttatatattattatatcctgatccctgcgttaaa--- : 265
YH1_LZ01 : cct--------------------gatcc-tgcgttta---- : 222
FT1_LZ01 : cct--------------------gatcc-tgcgttta---- : 224
CH1_LZ01 : cct--------------------gatcc-tgcgttta---- : 219
HW1_LZ01 : ttttttatatattattatatcctgatcc-tgcgttt----- : 238
BT1_LZ01 : ttttttatatattattatatcctgatcc-tgcgttta---- : 264
GF1_LZ01 : cct--------------------gatcc-tgcgttt----- : 224
SH2_LZ01 : cct--------------------gatcc-tgcgtta----- : 223
WS2_LZ01 : cct--------------------gatcc-tgcgtttaa--- : 220
WY2_LZ01 : cct--------------------gatcc-tgcgtta----- : 222
OW2_LZ01 : cct--------------------gatcc-tgcgttaaa--- : 224
WW2_LZ01 : cct--------------------gatcc-tgcgttta---- : 218
RE2_LZ01 : ttttttatatattattatatcctg-tcc-tgcgttaaa--- : 262
YH2_LZ01 : cct--------------------gatcc-tgcgttta---- : 223
FT2_LZ01 : cct--------------------gatcc-tgcgttta---- : 224
CH2_LZ01 : cct--------------------gatcc-tgcgttta---- : 223
HW2_LZ01 : ttttttatatattattatatcctgatcc-tgcgttt----- : 239
BT2_LZ01 : ttttttatatattattatatcctgatcc-tgcgttt----- : 260
GF2_LZ01 : cct--------------------gatcc-tgcgttta---- : 228
SH3_LZ01 : cct--------------------gatcc-tgcgttt----- : 229
WS3_LZ01 : cct--------------------gatcc-tgcgtta----- : 226
WY3_LZ01 : cct--------------------gatcc-tgcgtttaa--- : 232
OW3_LZ01 : cct--------------------gatcc-tgcgttta---- : 228
WW3_LZ01 : cct--------------------gatcc-tgcgttt----- : 225
RE3_LZ01 : ttttttatatattattatatcctgatcc-tgcgttta---- : 263
YH3_LZ01 : cct--------------------gatcc-tgcgtt------ : 225
FT3_LZ01 : cct--------------------gatcc-tgcgttt----- : 228
CH3_LZ01 : cct--------------------gatcc-tgcgttt----- : 228
HW3_LZ01 : ttttttatatattattatatcctgatcc-tgcgtt------ : 245
BT3_LZ01 : ttttttatatattattatatcctgatcc-tgcgtt------ : 263
GF3_LZ01 : cct--------------------gatcc-tgcgttaaa--- : 231
```

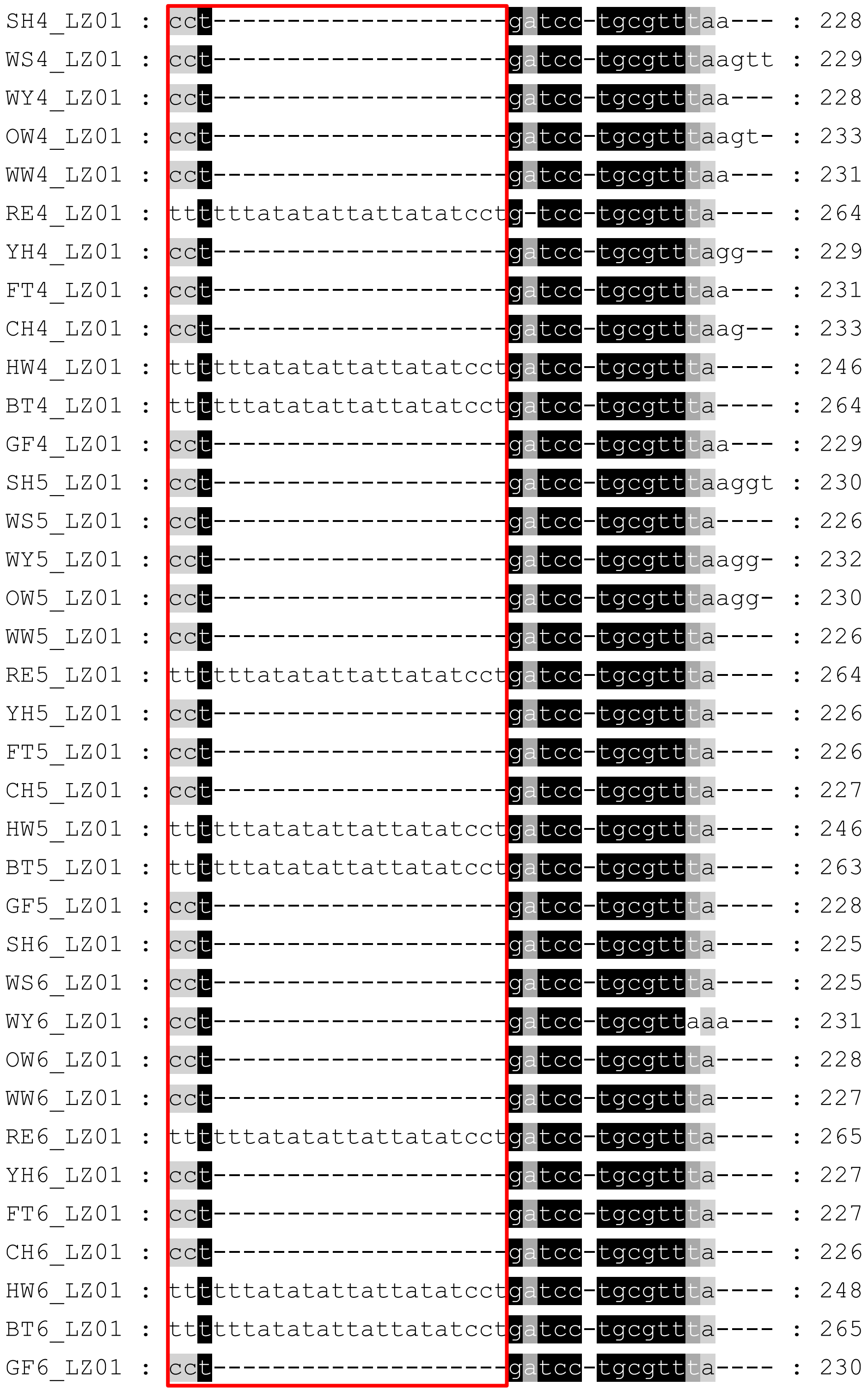

Figure S21. The alignment of amplicons produced by designed LZ01 primer. The SNP and Indel regions are highlighted with red squares. The nucleotides identical across all plastomes are shaded in black, whereas those conserved in 60 % of the sequences are shaded in gray. SH: *P. salicina* 'Sanhua plum'; WS: *P. salicina* 'Wanshuang plum'; WY: *P. salicina* 'Wuyuecui'; OW: *P. salicina* 'Oishiwase'; WW: *P. simonii* 'Weiwang'; RE: *P. domestica* 'Richard Early'; YH: *P. salicina* 'Yinhong plum'; FT: *P. salicina* 'Fengtang plum'; CH: *P. salicina* 'Cuihong plum'; HW: *P. cerasifera* 'Hollywood'; BT: *P. domestica* 'Bingtang plum'; GF: *P. salicina* 'No.2 Guofeng'. Arabic numerals represent different individuals.

```
SH1_LZ02 : ---gattccatcaccgtagtttttgtg-attcgtacaacttttgttcttt :  46
WS1_LZ02 : ----tttttcttcacgtagtttttgtgaattcgtacaacttttgttcttt :  46
WW1_LZ02 : ----tttatcatcccgtagtttttgtg-attcgtacaacttttgttcttt :  45
YH1_LZ02 : ------gtccatcacgtagtttttgtg-attcgtacaacttttgttcttt :  43
FT1_LZ02 : ------gatcatgacgtagtttttgtgaattcgtacaacttttgttcttt :  44
CH1_LZ02 : ------tcacatcccgtagtttttgtg-attcgtacaacttttgttcttt :  43
SH2_LZ02 : ------ttttcatacgtagtttttgtg-attcgtacaacttttgttcttt :  43
WS2_LZ02 : ----ttttcaatcacgtagtttttgtg-attcgtacaacttttgttcttt :  45
WW2_LZ02 : ----atctcaatcccgtagtttttgtg-attcgtacaacttttgttcttt :  45
YH2_LZ02 : -----ttttcatcacgtagtttttgtg-attcgtacaacttttgttcttt :  44
FT2_LZ02 : -----tcttcaatacgtagtttttgtg-attcgtacaacttttgttcttt :  44
CH2_LZ02 : ------gttcttcacgtagtttttgtg-attcgtacaacttttgttcttt :  43
SH3_LZ02 : -----tgttcattacgtagtttttgtg-attcgtacaacttttgttcttt :  44
WS3_LZ02 : ---------cttcccgtagtttttgtg-attcgtacaacttttgttcttt :  40
WW3_LZ02 : ---------ccgaccgtagtttttgtg-attcgtacaacttttgttcttt :  40
YH3_LZ02 : ---tgtttctttaccgtagtttttgtg-attcgtacaacttttgttcttt :  46
FT3_LZ02 : ---tggtttcatcacgtagtttttgtg-attcgtacaacttttgttcttt :  46
CH3_LZ02 : -----tgctcttcacgtagtttttgtg-attcgtacaacttttgttcttt :  44
SH4_LZ02 : ----ttgttcatcacgtagtttttgtg-attcgtacaacttttgttcttt :  45
WS4_LZ02 : -----tgatcatcacgtagtttttgtg-attcgtacaacttttgttcttt :  44
WW4_LZ02 : --taccttcatcccgtagtttttgtg-attcgtacaacttttgttcttt :  47
YH4_LZ02 : ----cacttcatcccgtagtttttgtg-attcgtacaacttttgttcttt :  45
FT4_LZ02 : ----tagggtcatacgtagtttttgtg-attcgtacaacttttgttcttt :  45
CH4_LZ02 : tttaggctccatcacgtagtttttgtg-attcgtacaacttttgttcttt :  49
SH5_LZ02 : -----ttgtcttcacgtagtttttgtg-attcgtacaacttttgttcttt :  44
WS5_LZ02 : ----ttagtcttcacgtagtttttgtg-attcgtacaacttttgttcttt :  45
WW5_LZ02 : ----taagtcttcacgtagtttttgtg-attcgtacaacttttgttcttt :  45
YH5_LZ02 : ---ttaggtcttcacgtagtttttgtg-attcgtacaacttttgttcttt :  46
FT5_LZ02 : -----tactcttcacgtagtttttgtg-attcgtacaacttttgttcttt :  44
CH5_LZ02 : ------atatcatccgtagtttttgtg-attcgtacaacttttgttcttt :  43
SH6_LZ02 : ------ttttcatacgtagtttttgtg-attcgtacaacttttgttcttt :  43
WS6_LZ02 : -----ttttcatcccgtagtttttgtgaattcgtacaacttttgttcttt :  45
WW6_LZ02 : ----tttttcatcacgtagtttttgtg-attcgtacaacttttgttcttt :  45
YH6_LZ02 : ----ttttccatcacgtagtttttgtg-attcgtacaacttttgttcttt :  45
FT6_LZ02 : ----atttcaatcacgtagtttttgtg-attcgtacaacttttgttcttt :  45
CH6_LZ02 : ---atttttcatcacgtagtttttgtg-attcgtacaacttttgttcttt :  46
```

```
SH1_LZ02 : tcttacatttctttctggtttctttttttgaaccgttctttgtttttttt : 96
WS1_LZ02 : tcttacatttctttctggtttctttttttgaaccgttctttgtttttttt : 96
WW1_LZ02 : tcttacatttctttctggtttctttttttgaaccgttctttgtttttttt : 95
YH1_LZ02 : tcttacatttctttctggtttctttttttgaaccgttctttgtttttttt : 93
FT1_LZ02 : tcttacatttctttctggtttctttttttgaaccgttctttg-ttttttt : 93
CH1_LZ02 : tcttacatttctttctggtttctttttttgaaccgttctttgtttttttt : 93
SH2_LZ02 : tcttacatttctttctggtttctttttttgaaccgttctttgtttttttt : 93
WS2_LZ02 : tcttacatttctttctggtttctttttttgaaccgttctttgtttttttt : 95
WW2_LZ02 : tcttacatttctttctggtttctttttttgaaccgttctttgtttttttt : 95
YH2_LZ02 : tcttacatttctttctggtttctttttttgaaccgttctttgtttttttt : 94
FT2_LZ02 : tcttacatttctttctggtttctttttttgaaccgttctttg-ttttttt : 93
CH2_LZ02 : tcttacatttctttctggtttctttttttgaaccgttctttgtttttttt : 93
SH3_LZ02 : tcttacatttctttctggtttctttttttgaaccgttctttgtttttttt : 94
WS3_LZ02 : tcttacatttctttctggtttctttttttgaaccgttctttgtttttttt : 90
WW3_LZ02 : tcttacatttctttctggtttctttttttgaaccgttctttgtttttttt : 90
YH3_LZ02 : tcttacatttctttctggtttctttttttgaaccgttctttgtttttttt : 96
FT3_LZ02 : tcttacatttctttctggtttctttttttgaaccgttctttg-ttttttt : 95
CH3_LZ02 : tcttacatttctttctggtttctttttttgaaccgttctttgtttttttt : 94
SH4_LZ02 : tcttacatttctttctggtttctttttttgaaccgttctttgtttttttt : 95
WS4_LZ02 : tcttacatttctttctggtttctttttttgaaccgttctttgtttttttt : 94
WW4_LZ02 : tcttacatttctttctggtttctttttttgaaccgttctttgtttttttt : 97
YH4_LZ02 : tcttacatttctttctggtttctttttttgaaccgttctttgtttttttt : 95
FT4_LZ02 : tcttacatttctttctggtttctttttttgaaccgttctttg-ttttttt : 94
CH4_LZ02 : tcttacatttctttctggtttctttttttgaaccgttctttgtttttttt : 99
SH5_LZ02 : tcttacatttctttctggtttctttttttgaaccgttctttgtttttttt : 94
WS5_LZ02 : tcttacatttctttctggtttctttttttgaaccgttctttgtttttttt : 95
WW5_LZ02 : tcttacatttctttctggtttctttttttgaaccgttctttgtttttttt : 95
YH5_LZ02 : tcttacatttctttctggtttctttttttgaaccgttctttgtttttttt : 96
FT5_LZ02 : tcttacatttctttctggtttctttttttgaaccgttctttg-ttttttt : 93
CH5_LZ02 : tcttacatttctttctggtttctttttttgaaccgttctttgtttttttt : 93
SH6_LZ02 : tcttacatttctttctggtttctttttttgaaccgttctttgtttttttt : 93
WS6_LZ02 : tcttacatttctttctggtttctttttttgaaccgttctttgtttttttt : 95
WW6_LZ02 : tcttacatttctttctggtttctttttttgaaccgttctttgtttttttt : 95
YH6_LZ02 : tcttacatttctttctggtttctttttttgaaccgttctttgtttttttt : 95
FT6_LZ02 : tcttacatttctttctggtttctttttttgaaccgttctttg-ttttttt : 94
CH6_LZ02 : tcttacatttctttctggtttctttttttgaaccgttctttgtttttttt : 96
```

```
SH1_LZ02 : tttttcgtgatttaaattcattcaatattcaattcacaattacagatgaa : 146
WS1_LZ02 : tttttcgtgatttaaattcattcaatattcaattcacaattacagatgaa : 146
WW1_LZ02 : tttttcgtgatttaaattcattcaatattcaattcacaattacagatgaa : 145
YH1_LZ02 : tttttcgtgatttaaattcattcaatattcaattcacaattacagatgaa : 143
FT1_LZ02 : tttttcgtgatttaaattcattcaatattcaattcacaattacagatgaa : 143
CH1_LZ02 : tttttcgtgatttaaattcattcaatattcaattcacaattacagatgaa : 143
SH2_LZ02 : tttttcgtgatttaaattcattcaatattcaattcacaattacagatgaa : 143
WS2_LZ02 : tttttcgtgatttaaattcattcaatattcaattcacaattacagatgaa : 145
WW2_LZ02 : tttttcgtgatttaaattcattcaatattcaattcacaattacagatgaa : 145
YH2_LZ02 : tttttcgtgatttaaattcattcaatattcaattcacaattacagatgaa : 144
FT2_LZ02 : tttttcgtgatttaaattcattcaatattcaattcacaattacagatgaa : 143
CH2_LZ02 : tttttcgtgatttaaattcattcaatattcaattcacaattacagatgaa : 143
SH3_LZ02 : tttttcgtgatttaaattcattcaatattcaattcacaattacagatgaa : 144
WS3_LZ02 : tttttcgtgatttaaattcattcaatattcaattcacaattacagatgaa : 140
WW3_LZ02 : tttttcgtgatttaaattcattcaatattcaattcacaattacagatgaa : 140
YH3_LZ02 : tttttcgtgatttaaattcattcaatattcaattcacaattacagatgaa : 146
FT3_LZ02 : tttttcgtgatttaaattcattcaatattcaattcacaattacagatgaa : 145
CH3_LZ02 : tttttcgtgatttaaattcattcaatattcaattcacaattacagatgaa : 144
SH4_LZ02 : tttttcgtgatttaaattcattcaatattcaattcacaattacagatgaa : 145
WS4_LZ02 : tttttcgggatttaaattcattcaatattcaattcacaattacagatgaa : 144
WW4_LZ02 : tttttcgtgatttaaattcattcaatattcaattcacaattacagatgaa : 147
YH4_LZ02 : tttttcgtgatttaaattcattcaatattcaattcacaattacagatgaa : 145
FT4_LZ02 : tttttcgtgatttaaattcattcaatattcaattcacaattacagatgaa : 144
CH4_LZ02 : tttttcgggatttaaattcattcaatattcaattcacaattacagatgaa : 149
SH5_LZ02 : tttttcgtgatttaaattcattcaatattcaattcacaattacagatgaa : 144
WS5_LZ02 : tttttcgtgatttaaattcattcaatattcaattcacaattacagatgaa : 145
WW5_LZ02 : tttttcgtgatttaaattcattcaatattcaattcacaattacagatgaa : 145
YH5_LZ02 : tttttcgtgatttaaattcattcaatattcaattcacaattacagatgaa : 146
FT5_LZ02 : tttttcgtgatttaaattcattcaatattcaattcacaattacagatgaa : 143
CH5_LZ02 : tttttcgggatttaaattcattcaatattcaattcacaattacagatgaa : 143
SH6_LZ02 : tttttcgtgatttaaattcattcaatattcaattcacaattacagatgaa : 143
WS6_LZ02 : tttttcgtgatttaaattcattcaatattcaattcacaattacagatgaa : 145
WW6_LZ02 : tttttcgtgatttaaattcattcaatattcaattcacaattacagatgaa : 145
YH6_LZ02 : tttttcgtgatttaaattcattcaatattcaattcacaattacagatgaa : 145
FT6_LZ02 : tttttcgtgatttaaattcattcaatattcaattcacaattacagatgaa : 144
CH6_LZ02 : tttttcgtgatttaaattcattcaatattcaattcacaattacagatgaa : 146
```

```
SH1_LZ02 : acggaaaaacttccattggaatccctatctaaattcacaatagtaggaca : 196
WS1_LZ02 : acggaaaaacttccattggaatccctatctaaattcacaatagtaggaca : 196
WW1_LZ02 : acggaaaaacttccattggaatccctatctaaattcacaatagtaggaca : 195
YH1_LZ02 : acggaaaaacttccattggaatccctatctaaattcacaatagtaggaca : 193
FT1_LZ02 : acggaagaacttccattggaatccctatctaaattcacaatagtaggaca : 193
CH1_LZ02 : acggaaaaacttccattggaatccctatctaaattcacaatagtaggaca : 193
SH2_LZ02 : acggaaaaacttccattggaatccctatctaaattcacaatagtaggaca : 193
WS2_LZ02 : acggaaaaacttccattggaatccctatctaaattcccaatagtaggaca : 195
WW2_LZ02 : acggaaaaacttccattggaatccctatctaaattcacaatagtaggaca : 195
YH2_LZ02 : acggaaaaacttccattggaatccctatctaaattcacaatagtaggaca : 194
FT2_LZ02 : acggaaaaacttccattggaatccctatctaaattcacaatagtaggaca : 193
CH2_LZ02 : acggaaaaacttccattggaatccctatctaaattcacaatagtaggaca : 193
SH3_LZ02 : acggaaaaacttccattggaatccctatctaaattcacaatagtaggaca : 194
WS3_LZ02 : acggaaaaacttccattggaatccctatctaaattcacaatagtaggaca : 190
WW3_LZ02 : acggaaaaacttccattggaatccctatctaaattcacaatagtaggaca : 190
YH3_LZ02 : acggaaaaacttccattggaatccctatctaaattcacaatagtaggaca : 196
FT3_LZ02 : acggaaaaacttccattggaatccctatctaaattcacaatagtaggaca : 195
CH3_LZ02 : acggaaaaacttccattggaatccctatctaaattcacaatagtaggaca : 194
SH4_LZ02 : acggaaaaacttccattggaatccctatctaaattcacaatagtaggaca : 195
WS4_LZ02 : acggaaaaacttccattggaatccctatctaaattcacaatagtaggaca : 194
WW4_LZ02 : acggaaaaacttccattggaatccctatctaaattcacaatagtaggaca : 197
YH4_LZ02 : acggaaaaacttccattggaatccctatctaaattcacaatagtaggaca : 195
FT4_LZ02 : acggaagaacttccattggaatccctatctaaattcacaatagtaggaca : 194
CH4_LZ02 : acggaaaaacttccattggaatccctatctaaattcacaatagtaggaca : 199
SH5_LZ02 : acggaaaaacttccattggaatccctatctaaattcacaatagtaggaca : 194
WS5_LZ02 : acggaaaaacttccattggaatccctatctaaattcacaatagtaggaca : 195
WW5_LZ02 : acggaaaaacttccattggaatccctatctaaattcacaatagtaggaca : 195
YH5_LZ02 : acggaaaaacttccattggaatccctatctaaattcacaatagtaggaca : 196
FT5_LZ02 : acggaaaaacttccattggaatccctatctaaattcacaatagtaggaca : 193
CH5_LZ02 : acggaaaaacttccattggaatccctatctaaattcacaatagtaggaca : 193
SH6_LZ02 : acggaaaaacttccattggaatccctatctaaattcacaatagtaggaca : 193
WS6_LZ02 : acggaaaaacttccattggaatccctatctaaattcacaatagtaggaca : 195
WW6_LZ02 : acggaaaaacttccattggaatccctatctaaattcacaatagtaggaca : 195
YH6_LZ02 : acggaaaaacttccattggaatccctatctaaattcacaatagtaggaca : 195
FT6_LZ02 : acggaaaaacttccattggaatccctatctaaattcacaatagtaggaca : 194
CH6_LZ02 : acggaaaaacttccattggaatccctatctaaattcacaatagtaggaca : 196
```

```
SH1_LZ02 : aattaaatatatctttagttcatatatacctagttaatatcacatataca : 246
WS1_LZ02 : aattaaatatatctttagttcatatatacctagttaatatcacatataca : 246
WW1_LZ02 : aattaaatatatctttagttcatatatacctagttaatatcacatataca : 245
YH1_LZ02 : aattaaatatatctttagttcatatatacctagttaatatcacatataca : 243
FT1_LZ02 : aattaaatatatctttagttcatatatacctagttaatatcacatataca : 243
CH1_LZ02 : aattaaatatatctttagttcatatatacctagttaatatcacatataca : 243
SH2_LZ02 : aattaaatatatctttagttcatatatacctagttaatatcacatataca : 243
WS2_LZ02 : aattaaatatatctttagttcatatatacctagttaatatcacatataca : 245
WW2_LZ02 : aattaaatatatctttagttcatatatacctagttaatatcacatataca : 245
YH2_LZ02 : aattaaatatatctttagttcatatatacctagttaatatcacatataca : 244
FT2_LZ02 : aattaaatatatctttagttcatatatacctagttaatatcacatataca : 243
CH2_LZ02 : aattaaatatatctttagttcatatatacctagttaatatcacatataca : 243
SH3_LZ02 : aattaaatatatctttagttcatatatacctagttaatatcacatataca : 244
WS3_LZ02 : aattaaatatatctttagttcatatatacctagttaatatcacatataca : 240
WW3_LZ02 : aattaaatatatctttagttcatatatacctagttaatatcacatataca : 240
YH3_LZ02 : aattaaatatatctttagttcatatatacctagttaatatcacatataca : 246
FT3_LZ02 : aattaaatatatctttagttcatatatacctagttaatatcacatataca : 245
CH3_LZ02 : aattaaatatatctttagttcatatatacctagttaatatcacatataca : 244
SH4_LZ02 : aattaaatatatctttagttcatatatacctagttaatatcacatataca : 245
WS4_LZ02 : aattaaatatatctttagttcatatatacctagttaatatcacatataca : 244
WW4_LZ02 : aattaaatatatctttagttcatatatacctagttaatatcacatataca : 247
YH4_LZ02 : aattaaatatatctttagttcatatatacctagttaatatcacatataca : 245
FT4_LZ02 : aattagatatatctttagttcatatatacctagttaatatcacatataca : 244
CH4_LZ02 : aattaaatatatctttagttcatatatacctagttaatatcacatataca : 249
SH5_LZ02 : aattaaatatatctttagttcatatatacctagttaatatcacatataca : 244
WS5_LZ02 : aattaaatatatctttagttcatatatacctagttaatatcacatataca : 245
WW5_LZ02 : aattaaatatatctttagttcatatatacctagttaatatcacatataca : 245
YH5_LZ02 : aattaaatatatctttatttcatatatacctagttaatatcacatataca : 246
FT5_LZ02 : aattaaatatatctttagttcatatatacctagttaatatcacatataca : 243
CH5_LZ02 : aattaaatatatctttagttcatatatacctagttaatatcacatatact : 243
SH6_LZ02 : aattaaatatatctttagttcatatatacctagttaatatcacatataca : 243
WS6_LZ02 : aattaaatatatctttagttcatatatacctagttaatatcacatataca : 245
WW6_LZ02 : aattaaatatatctttagttcatatatacctagttaatatcacatataca : 245
YH6_LZ02 : aattaaatatatctttagttcatatatacctagttaatatcacatataca : 245
FT6_LZ02 : aattaaatatatctttagttcatatatacctagttaatatcacatataca : 244
CH6_LZ02 : aattaaatatatctttagttcatatatacctagttaatatcacatataca : 246
```

```
SH1_LZ02 : tgcctttccaca-- : 258
WS1_LZ02 : tgcctttccaca-- : 258
WW1_LZ02 : tgcctttccaca-- : 257
YH1_LZ02 : tgcctttccaca-- : 255
FT1_LZ02 : tgcctttcccca-- : 255
CH1_LZ02 : tgcctttccaca-- : 255
SH2_LZ02 : tgcctttccaca-- : 255
WS2_LZ02 : tgcctttccaca-- : 257
WW2_LZ02 : tgcctttccaca-- : 257
YH2_LZ02 : ggcctttccaca-- : 256
FT2_LZ02 : tgcctttcccca-- : 255
CH2_LZ02 : tgcctttccaca-- : 255
SH3_LZ02 : tgcctttccaaa-- : 256
WS3_LZ02 : tgcctttccaca-- : 252
WW3_LZ02 : tgcctttccaaa-- : 252
YH3_LZ02 : tgcctttccaca-- : 258
FT3_LZ02 : tgcctttcccca-- : 257
CH3_LZ02 : tgcctttccaca-- : 256
SH4_LZ02 : tgcctttcccca-- : 257
WS4_LZ02 : ggcctttcccaa-- : 256
WW4_LZ02 : ggcctttccaaa-- : 259
YH4_LZ02 : ggcctttcccaa-- : 257
FT4_LZ02 : tgcctttccaaa-- : 256
CH4_LZ02 : ggcctttccaaa-- : 261
SH5_LZ02 : ggcctttcccca-- : 256
WS5_LZ02 : tgcctttcccaa-- : 257
WW5_LZ02 : ggcctttcccca-- : 257
YH5_LZ02 : tgcctttcccca-- : 258
FT5_LZ02 : ggcctttcccca-- : 255
CH5_LZ02 : ggcctttcccaa-- : 255
SH6_LZ02 : gccctttcccaa-- : 255
WS6_LZ02 : tgcctttcccaa-- : 257
WW6_LZ02 : tgcctttcccca-- : 257
YH6_LZ02 : tgcctttcccaa-- : 257
FT6_LZ02 : tgcctttcccca-- : 256
CH6_LZ02 : ggcctttcccca-- : 258
```

Figure S22. The alignment of amplicons produced by designed LZ02 primer. The Indel regions are highlighted with red squares. The nucleotides identical across all plastomes are shaded in black, whereas those conserved in 60 % of the sequences are shaded in gray. SH: *P. salicina* 'Sanhua plum'; WS: *P. salicina* 'Wanshuang plum'; WW: *P. simonii* 'Weiwang'; YH: *P. salicina* 'Yinhong plum'; FT: *P. salicina* 'Fengtang plum' and CH: *P. salicina* 'Cuihong plum'. Arabic numerals represent different individuals.

```
SH1_LZ03 : ccaa---accctatagaatttttcgtac-taaacttttttcgtggattca : 46
WS1_LZ03 : acaaaa-aaaatatagaatttttcgtac-taaacttttttcgtggattca : 48
WW1_LZ03 : ttaaa--aactaatagaatttttcgtac-taaacttttttcgtggattca : 47
YH1_LZ03 : caaaa--atcgtata-gatttttcgtac-taaacttttttcgtggattca : 46
FT1_LZ03 : caa----aacatata-gatttttcgtac-taaacttttttcgtggattca : 44
CH1_LZ03 : ca-----aacatata-gatttttcgtac-taaacttttttcgtggattca : 43
SH2_LZ03 : ccata--aacatata-gatttttcgtac-taaacttttttcgtggattca : 46
WS2_LZ03 : gctt---aacatataggatttttcgtac-taaacttttttcgtggattca : 46
WW2_LZ03 : ccaa---aacgtata-gatttttcgtac-taaacttttttcgtggattca : 45
YH2_LZ03 : caa----aaaatatagaatttttcgtac-taaacttttttcgtggattca : 45
FT2_LZ03 : ct-----aaactatagaatttttcgtac-taaacttttttcgtggattca : 44
CH2_LZ03 : ca-----aaaatata-gatttttcgtac-taaacttttttcgtggattca : 43
SH3_LZ03 : aaa----ataataga-aatttttcgtac-taaacttttttcgtggattca : 44
WS3_LZ03 : gcctaacaatatagg-aatttttcgtac-taaacttttttcgtggattca : 48
WW3_LZ03 : ccat---aacgtata-gatttttcgtac-taaacttttttcgtggattca : 45
YH3_LZ03 : ccat---aacatata-gatttttcgtac-taaacttttttcgtggattca : 45
FT3_LZ03 : ccaaaaaccatataagaatttttcgtac-taaacttttttcgtggattca : 49
CH3_LZ03 : cta----aaaatatagaatttttcgtac-taaacttttttcgtggattca : 45
SH4_LZ03 : ccta---aacatata-gatttttcgtac-taaacttttttcgtggattca : 45
WS4_LZ03 : ccctaataacatattagatttttcgtac-taaacttttttcgtggattca : 49
WW4_LZ03 : tcctat-acaggata-gatttttcgtac-taaacttttttcgtggattca : 47
YH4_LZ03 : tcctta-cacatata-gatttttcgtac-taaacttttttcgtggattca : 47
FT4_LZ03 : cctt---aacatata-gatttttcgtac-taaacttttttcgtggattca : 45
CH4_LZ03 : tccta--aacatata-gatttttcgtac-taaacttttttcgtggattca : 46
SH5_LZ03 : ctaaaa-acataatagaatttttcgtac-taaacttttttcgtggattca : 48
WS5_LZ03 : caaaa--accatatagaatttttcgtac-taaacttttttcgtggattca : 47
WW5_LZ03 : ccaa---aacatatagaatttttcgtac-taaacttttttcgtggattca : 46
YH5_LZ03 : caaaaa-aaattatagaatttttcgtac-taaacttttttcgtggattca : 48
FT5_LZ03 : caaaaaaacatattagaatttttcgtac-taaacttttttcgtggattca : 49
CH5_LZ03 : aaaaaa-acatataagaatttttcgtac-taaacttttttcgtggattca : 48
SH6_LZ03 : tcaatc-ccattatagaatttttcgtacttcaacttttttcgtggattca : 49
WS6_LZ03 : agcata-aacgtata-gatttttcgtac-taaacttttttcgtggattca : 47
WW6_LZ03 : agctt--aacgtata-gatttttcgtac-taaacttttttcgtggattca : 46
YH6_LZ03 : cgcta--aacatata-gatttttcgtac-taaacttttttcgtggattca : 46
FT6_LZ03 : tctca--tacatata-gatttttcgtac-taaacttttttcgtggattca : 46
CH6_LZ03 : ccct---aacatata-gatttttcgtac-taaacttttttcgtggattca : 45
```

```
SH1_LZ03 : -tcggttcattgttaaaaaaaaaattctcgtagaagagagatgtttttac : 95
WS1_LZ03 : -tcggttcattgttaaaaaaaaaattctcgtagaagagagatgtttttac : 97
WW1_LZ03 : -tcggttcattgttaaaaaaaaaattctcgtagaagagagatgtttttac : 96
YH1_LZ03 : -tcggttcattgttaaaaaaaaaattctcgtagaagagagatgtttttac : 95
FT1_LZ03 : -tcggttcattgttaaaaaaaaaattctcgtagaagagagatgtttttac : 93
CH1_LZ03 : -tcggttcattgttaaaaaaaaaattctcgtagaagagagatgcttttac : 92
SH2_LZ03 : -tcggttcattgttaaaaaaaaaattctcgtagaagagagatgtttttac : 95
WS2_LZ03 : -tcggttcattgttaaaaaaaaaattctcgtagaagagagatgtttttac : 95
WW2_LZ03 : -tcggttcattgttaaaaaaaaaattctcgtagaagagagatgtttttac : 94
YH2_LZ03 : -tcggttcattgttaaaaaaaaaattctcgtagaagagagatgtttttac : 94
FT2_LZ03 : -tcggttcattgttaaaaaaaaaattctcgtagaagagagatgtttttac : 93
CH2_LZ03 : -tcggttcattgttaaaaaaaaaattctcgtagaagagagatgcttttac : 92
SH3_LZ03 : -tcggttcattgttaaaaaaaaaattctcgtagaagagagatgtttttac : 93
WS3_LZ03 : -tcggttcattgttaaaaaaaaaattctcgtagaagagagatgtttttac : 97
WW3_LZ03 : -tcggttcattgttaaaaaaaaaattctcgtagaagagagatgtttttac : 94
YH3_LZ03 : -tcggttcattgttaaaaaaaaaattctcgtagaagagagatgtttttac : 94
FT3_LZ03 : -tcggttcattgttaaaaaaaaaattctcgtagaagagagatgtttttac : 98
CH3_LZ03 : -tcggttcattgttaaaaaaaaaattctcgtagaagagagatgcttttac : 94
SH4_LZ03 : -tcggttcattgttaaaaaaaaaattctcgtagaagagagatgtttttac : 94
WS4_LZ03 : -tcggttcattgttaaaaaaaaaattctcgtagaagagagatgtttttac : 98
WW4_LZ03 : -tcggttcattgttaaaaaaaaaattctcgtagaagagagatgtttttac : 96
YH4_LZ03 : -tcggttcattgttaaaaaaaaaattctcgtagaagagagatgtttttac : 96
FT4_LZ03 : -tcggttcattgttaaaaaaaaaattctcgtagaagagagatgtttttac : 94
CH4_LZ03 : -tcggttcattgttaaaaaaaaaattctcgtagaagagagatgcttttac : 95
SH5_LZ03 : -tcggttcattgttaaaaaaaaaattctcgtagaagagagatgtttttac : 97
WS5_LZ03 : -tcggttcattgttaaaaaaaaaattctcgtagaagagagatgtttttac : 96
WW5_LZ03 : -tcggttcattgttaaaaaaaaaattctcgtagaagagagatgtttttac : 95
YH5_LZ03 : -tcggttcattgttaaaaaaaaaattctcgtagaagagagatgtttttac : 97
FT5_LZ03 : -tcggttcattgttaaaaaaaaaattctcgtagaagagagatgtttttac : 98
CH5_LZ03 : -tcggttcattgttaaaaaaaaaattctcgtagaagagagatgcttttac : 97
SH6_LZ03 : ttcggttcattgttaaaaaaaaaattctcgtagaagagagatgtttttac : 99
WS6_LZ03 : -tcggttcattgttaaaaaaaaaattctcgtagaagagagatgtttttac : 96
WW6_LZ03 : -tcggttcattgttaaaaaaaaaattctcgtagaagagagatgtttttac : 95
YH6_LZ03 : -tcggttcattgttaaaaaaaaaattctcgtagaagagagatgtttttac : 95
FT6_LZ03 : -tcggttcattgttaaaaaaaaaattctcgtagaagagagatgtttttac : 95
CH6_LZ03 : -tcggttcattgttaaaaaaaaaattctcgtagaagagagatgcttttac : 94
```

```
SH1_LZ03 : ctcttttttttttagtcgaatttgtagaatatgattgaagtgtataattga : 145
WS1_LZ03 : ctcttttttttttagtcgaatttgtagaatatgattgaagtgtataattga : 147
WW1_LZ03 : ctcttttttttttagtcgaatttgtagaatatgattgaagtgtataattga : 146
YH1_LZ03 : ctcttttttttttagtcgaatttgtagaatatgattgaagtgtataattga : 145
FT1_LZ03 : ctcttttttttttagtcgaatttgtagaatatgattgaagtgtataattga : 143
CH1_LZ03 : ctcttttttttttagtcgaatttgtagaatatgattgaagtgtataattga : 142
SH2_LZ03 : ctcttttttttttagtcgaatttgtagaatatgattgaagtgtataattga : 145
WS2_LZ03 : ctcttttttttttagtcgaatttgtagaatatgattgaagtgtataattga : 145
WW2_LZ03 : ctcttttttttttagtcgaatttgtagaatatgattgaagtgtataattga : 144
YH2_LZ03 : ctcttttttttttagtcgaatttgtagaatatgattgaagtgtataattga : 144
FT2_LZ03 : ctcttttttttttagtcgaatttgtagaatatgattgaagtgtataattga : 143
CH2_LZ03 : ctcttttttttttagtcgaatttgtagaatatgattgaagtgtataattga : 142
SH3_LZ03 : ctcttttttttttagtcgaatttgtagaatatgattgaagtgtataattga : 143
WS3_LZ03 : ctcttttttttttagtcgaatttgtagaatatgattgaagtgtataattga : 147
WW3_LZ03 : ctcttttttttttagtcgaatttgtagaatatgattgaagtgtataattga : 144
YH3_LZ03 : ctcttttttttttagtcgaatttgtagaatatgattgaagtgtataattga : 144
FT3_LZ03 : ctcttttttttttagtcgaatttgtagaatatgattgaagtgtataattga : 148
CH3_LZ03 : ctcttttttttttagtcgaatttgtagaatatgattgaagtgtataattga : 144
SH4_LZ03 : ctcttttttttttagtcgaatttgtagaatatgattgaagtgtataattga : 144
WS4_LZ03 : ctcttttttttttagtcgaatttgtagaatatgattgaagtgtataattga : 148
WW4_LZ03 : ctcttttttttttagtcgaatttgtagaatatgattgaagtgtataattga : 146
YH4_LZ03 : ctcttttttttttagtcgaatttgtagaatatgattgaagtgtataattga : 146
FT4_LZ03 : ctcttttttttttagtcgaatttgtagaatatgattgaagtgtataattga : 144
CH4_LZ03 : ctcttttttttttagtcgaatttgtagaatatgattgaagtgtataattga : 145
SH5_LZ03 : ctcttttttttttagtcgaatttgtagaatatgattgaagtgtataattga : 147
WS5_LZ03 : ctcttttttttttagtcgaatttgtagaatatgattgaagtgtataattga : 146
WW5_LZ03 : ctcttttttttttagtcgaatttgtagaatatgattgaagtgtataattga : 145
YH5_LZ03 : ctcttttttttttagtcgaatttgtagaatatgattgaagtgtataattga : 147
FT5_LZ03 : ctcttttttttttagtcgaatttgtagaatatgattgaagtgtataattga : 148
CH5_LZ03 : ctcttttttttttagtcgaatttgtagaatatgattgaagtgtataattga : 147
SH6_LZ03 : ctcttttttttttagtcgaatttgtagaatatgattgaagtgtataattga : 149
WS6_LZ03 : ctcttttttttttagtcgaatttgtagaatatgattgaagtgtataattga : 146
WW6_LZ03 : ctcttttttttttagtcgaatttgtagaatatgattgaagtgtataattga : 145
YH6_LZ03 : ctcttttttttttagtcgaatttgtagaatatgattgaagtgtataattga : 145
FT6_LZ03 : ctcttttttttttagtcgaatttgtagaatatgattgaagtgtataattga : 145
CH6_LZ03 : ctcttttttttttagtcgaatttgtagaatatgattgaagtgtataattga : 144
```

```
SH1_LZ03 : agtgtataataaggctgattttgattaataaacatgtgcagatctaaatc : 195
WS1_LZ03 : agtgtataataaggctgattttgattaataaacatgtgcagatctaaatc : 197
WW1_LZ03 : agtgtataataaggctgattttgattaataaacatgtgcagatctaaatc : 196
YH1_LZ03 : agtgtataataaggctgattttgattaataaacatgtgcagatctaaatc : 195
FT1_LZ03 : agtgtataataaggctgattttgattaataaacatgtgcagatctaaatc : 193
CH1_LZ03 : agtgtataataaggctgattttgattaataaacatgtgcagatctaaatc : 192
SH2_LZ03 : agtgtataataaggctgattttgattaataaacatgtgcagatctaaatc : 195
WS2_LZ03 : agtgtataataaggctgattttgattaataaacatgtgcagatctaaatc : 195
WW2_LZ03 : agtgtataataaggctgattttgattaataaacatgtgcagatctaaatc : 194
YH2_LZ03 : agtgtataataaggctgattttgattaataaacatgtgcagatctaaatc : 194
FT2_LZ03 : agtgtataataaggctgattttgattaataaacatgtgcagatctaaatc : 193
CH2_LZ03 : agtgtataataaggctgattttgattaataaacatgtgcagatctaaatc : 192
SH3_LZ03 : agtgtataataaggctgattttgattaataaacatgtgcagatctaaatc : 193
WS3_LZ03 : agtgtataataaggctgattttgattaataaacatgtgcagatctaaatc : 197
WW3_LZ03 : agtgtataataaggctgattttgattaataaacatgtgcagatctaaatc : 194
YH3_LZ03 : agtgtataataaggctgattttgattaataaacatgtgcagatctaaatc : 194
FT3_LZ03 : agtgtataataaggctgattttgattaataaacatgtgcagatctaaatc : 198
CH3_LZ03 : agtgtataataaggctgattttgattaataaacatgtgcagatctaaatc : 194
SH4_LZ03 : agtgtataataaggctgattttgattaataaacatgtgcagatctaaatc : 194
WS4_LZ03 : agtgtataataaggctgattttgattaataaacatgtgcagatctaaatc : 198
WW4_LZ03 : agtgtataataaggctgattttgattaataaacatgtgcagatctaaatc : 196
YH4_LZ03 : agtgtataataaggctgattttgattaataaacatgtgcagatctaaatc : 196
FT4_LZ03 : agtgtataataaggctgattttgattaataaacatgtgcagatctaaatc : 194
CH4_LZ03 : agtgtataataaggctgattttgattaataaacatgtgcagatctaaatc : 195
SH5_LZ03 : agtgtataataaggctgattttgattaataaacatgtgcagatctaaatc : 197
WS5_LZ03 : agtgtataataaggctgattttgattaataaacatgtgcagatctaaatc : 196
WW5_LZ03 : agtgtataataaggctgattttgattaataaacatgtgcagatctaaatc : 195
YH5_LZ03 : agtgtataataaggctgattttgattaataaacatgtgcagatctaaatc : 197
FT5_LZ03 : agtgtataataaggctgattttgattaataaacatgtgcagatctaaatc : 198
CH5_LZ03 : agtgtataataaggctgattttgattaataaacatgtgcagatctaaatc : 197
SH6_LZ03 : agtgtataataaggctgattttgattaataaacatgtgcagatctaaatc : 199
WS6_LZ03 : agtgtataataaggctgattttgattaataaacatgtgcagatctaaatc : 196
WW6_LZ03 : agtgtataataaggctgattttgattaataaacatgtgcagatctaaatc : 195
YH6_LZ03 : agtgtataataaggctgattttgattaataaacatgtgcagatctaaatc : 195
FT6_LZ03 : agtgtataataaggctgattttgattaataaacatgtgcagatctaaatc : 195
CH6_LZ03 : agtgtataataaggctgattttgattaataaacatgtgcagatctaaatc : 194
```

```
SH1_LZ03 : taactatagctatctatatatacatatgccgcttcccctttattagagct : 245
WS1_LZ03 : taactatagctatctatatatacatatgccgcttcccctttattagagct : 247
WW1_LZ03 : taactatagctatctatatatacatatgccgcttcccctttattagagct : 246
YH1_LZ03 : taactatagctatctatatatacatatgccgcttcccctttattagagct : 245
FT1_LZ03 : taactatagctatctatatatacatatgccgcttcccctttattagagct : 243
CH1_LZ03 : taactatagctatctatatatacatatgccgcttcccctttattagagct : 242
SH2_LZ03 : taactatagctatctatatatacatatgccgcttcccctttattagagct : 245
WS2_LZ03 : taactatagctatctatatatacatatgccgcttcccctttattagagct : 245
WW2_LZ03 : taactatagctatctatatatacatatgccgcttcccctttattagagct : 244
YH2_LZ03 : taactatagctatctatatatacatatgccgcttcccctttattagagct : 244
FT2_LZ03 : taactatagctatctatatatacatatgccgcttcccctttattagagct : 243
CH2_LZ03 : taactatagctatctatatatacatatgccgcttcccctttattagagct : 242
SH3_LZ03 : taactatagctatctatatatacatatgccgcttcccctttattagagct : 243
WS3_LZ03 : taactatagctatctatatatacatatgccgcttcccctttattagagct : 247
WW3_LZ03 : taactatagctatctatatatacatatgccgcttcccctttattagagct : 244
YH3_LZ03 : taactatagctatctatatatacatatgccgcttcccctttattagagct : 244
FT3_LZ03 : taactatagctatctatatatacatatgccgcttcccctttattagagct : 248
CH3_LZ03 : taactatagctatctatatatacatatgccgcttcccctttattagagct : 244
SH4_LZ03 : taactatagctatctatatatacatatgccgcttcccctttattagagct : 244
WS4_LZ03 : taactatagctatctatatatacatatgccgcttcccctttattagagct : 248
WW4_LZ03 : taactatagctatctatatatacatatgccgcttcccctttattagagct : 246
YH4_LZ03 : taactatagctatctatatatacatatgccgcttcccctttattagagct : 246
FT4_LZ03 : taactatagctatctatatatacatatgccgcttcccctttattagagct : 244
CH4_LZ03 : taactatagctatctatatatacatatgccgcttcccctttattagagct : 245
SH5_LZ03 : taactatagctatctatatatacatatgccgcttcccctttattagagct : 247
WS5_LZ03 : taactatagctatctatatatacatatgccgcttcccctttattagagct : 246
WW5_LZ03 : taactatagctatctatatatacatatgccgcttcccctttattagagct : 245
YH5_LZ03 : taactatagctatctatatatacatatgccgcttcccctttattagagct : 247
FT5_LZ03 : taactatagctatctatatatacatatgccgcttcccctttattagagct : 248
CH5_LZ03 : taactatagctatctatatatacatatgccgcttcccctttattagagct : 247
SH6_LZ03 : taactatagctatctatatatacatatgccgcttcccctttattagagct : 249
WS6_LZ03 : taactatagctatctatatatacatatgccgcttcccctttattagagct : 246
WW6_LZ03 : taactatagctatctatatatacatatgccgcttcccctttattagagct : 245
YH6_LZ03 : taactatagctatctatatatacatatgccgcttcccctttattagagct : 245
FT6_LZ03 : taactatagctatctatatatacatatgccgcttcccctttattagagct : 245
CH6_LZ03 : taactatagctatctatatatacatatgccgcttcccctttattagagct : 244
```

```
SH1_LZ03 : ctattcaggacagaacatgtt---- : 266
WS1_LZ03 : ctattcaggacagaacatgtt---- : 268
WW1_LZ03 : ctattcaggacagaacatgtta--- : 268
YH1_LZ03 : ctattcaggacagaacatgtaaa-- : 268
FT1_LZ03 : ctattcaggacagaacatgtta--- : 265
CH1_LZ03 : ctattcaggacagaacatgttaa-- : 265
SH2_LZ03 : ctattcaggacagaacatgtta--- : 267
WS2_LZ03 : ctattcaggacagaacatgtta--- : 267
WW2_LZ03 : ctattcaggacagaacatgtta--- : 266
YH2_LZ03 : ctattcaggacagaacatgtt---- : 265
FT2_LZ03 : ctattcaggacagaacatgtt---- : 264
CH2_LZ03 : ctattcaggacagaacatgtta--- : 264
SH3_LZ03 : ctattcaggacagaacatgtta--- : 265
WS3_LZ03 : ctattcaggacagaacatgtta--- : 269
WW3_LZ03 : ctattcaggacagaacatgtta--- : 266
YH3_LZ03 : ctattcaggacagaacatgtta--- : 266
FT3_LZ03 : ctattcaggacagaacatgtta--- : 270
CH3_LZ03 : ctattcaggacagaacatgtta--- : 266
SH4_LZ03 : ctattcaggacagaacatgtta--- : 266
WS4_LZ03 : ctattcagga-ataacatgtta--- : 269
WW4_LZ03 : ctattcagga-agaacatgtta--- : 267
YH4_LZ03 : ctattcaggacagaacatgtta--- : 268
FT4_LZ03 : ctattcaggacagaacatgtta--- : 266
CH4_LZ03 : ctattcaggacagaacatgtta--- : 267
SH5_LZ03 : ctattcaggaataaacatgttaa-- : 270
WS5_LZ03 : ctattcaggacagaacatgttaa-- : 269
WW5_LZ03 : ctattcagga-ataacatgttaa-- : 267
YH5_LZ03 : ctattcaggacagaacatgttaa-- : 270
FT5_LZ03 : ctattcaggaagcaacatgttaacc : 273
CH5_LZ03 : ctattcaggacagaacatgttaa-- : 270
SH6_LZ03 : ctattcaggaagcaacatgttaa-- : 272
WS6_LZ03 : ctattcagga-agaacatgttaa-- : 268
WW6_LZ03 : ctattcagga-agaacatgtta--- : 266
YH6_LZ03 : ctattcaggacagaacatgtta--- : 267
FT6_LZ03 : ctattcagga-agaacatgtta--- : 266
CH6_LZ03 : ctattcagga-agaacatgtta--- : 265
```

Figure S23. The alignment of amplicons produced by designed LZ03 primer. The SNP regions are highlighted with red squares. The nucleotides identical across all plastomes are shaded in black, whereas those conserved in 60 % of the sequences are shaded in gray. SH: *P. salicina* 'Sanhua plum'; WS: *P. salicina* 'Wanshuang plum'; WW: *P. simonii* 'Weiwang'; YH: *P. salicina* 'Yinhong plum'; FT: *P. salicina* 'Fengtang plum' and CH: *P. salicina* 'Cuihong plum'. Arabic numerals represent different individuals.

```
SH1_LZ04 : ------ctccgaaagtatcata-t----ggat--gg-aatatttagtaca : 36
WS1_LZ04 : --------tggaatggaaaata-t----ggat--gg-aatatttagtaca : 34
WW1_LZ04 : ------acgggatgggatcata-t----ggat--ggaaatatttagtaca : 37
YH1_LZ04 : -----accgaaatggaatcata-t----ggat--gg-aatatttagtaca : 37
FT1_LZ04 : ------aacgaatggaatcata-t----ggat--gg-aatatttagtaca : 36
CH1_LZ04 : ------aaacgaatgaatcata-t----ggat--gg-aatatttagtaca : 36
SH2_LZ04 : -----cttcgaaatggatcata-t----ggat--ggaaatatttagtaca : 38
WS2_LZ04 : --------tggaattgataata-t----ggat--gg-aatatttagtaca : 34
WW2_LZ04 : ---------tgaattgtatata-t----ggat--ggaaatatttagtaca : 34
YH2_LZ04 : -------ctcggattggatcta-t----ggat--ggaaatatttagtaca : 36
FT2_LZ04 : -----cacgaatttgtatcata-t----ggat--ggaaatatttagtaca : 38
CH2_LZ04 : -------tggaatgttataata-t----ggat--gg-aatatttagtaca : 35
SH3_LZ04 : -----acccgttatgtataata-t----ggat--ggaaatatttagtaca : 38
WS3_LZ04 : -aaattcggatatggaataattat----ggat--gg-aatatttagtaca : 42
WW3_LZ04 : -----acatcgggattatcata-t----ggat--gg-aatatttagtaca : 37
YH3_LZ04 : ------atggtatgtaatcata-t----ggat--gg-aatatttagtaca : 36
FT3_LZ04 : ------atcgaaatgaatcata-t----ggat--gg-aatatttagtaca : 36
CH3_LZ04 : ----------tgatggttaata-t----ggat--ggaaatatttagtaca : 33
SH4_LZ04 : -------tcacgcatgatcata-t----ggat--gg-aatatttagtaca : 35
WS4_LZ04 : --------tatcgctgatcata-t----ggat--gg-aatatttagtaca : 34
WW4_LZ04 : --------tcacgctgatcata-t----ggat--gg-aatatttagtaca : 34
YH4_LZ04 : -------acgacgatgatcata-t----ggat--gg-aatatttagtaca : 35
FT4_LZ04 : ------cccacggatgatcata-t----ggat--gg-aatatttagtaca : 36
CH4_LZ04 : --------tatcgctgatcata-t----ggat--gg-aatatttagtaca : 34
SH5_LZ04 : -gaacccttcggaatgttcata-t----ggat--gg-aatatttagtaca : 41
WS5_LZ04 : ------aaggtttggtttcata-t----ggat--ggaaatatttagtaca : 37
WW5_LZ04 : ------caccaattggatcata-t----ggat--gg-aatatttagtaca : 36
YH5_LZ04 : ----aaccgtatgggaataata-t----ggat--ggaaatatttagtaca : 39
FT5_LZ04 : ctcggaatggtattcaattatg------ggat--ggaaatatttagtaca : 42
CH5_LZ04 : ------acgattttgtttaatatt----ggat--ggaaatatttagtaca : 38
SH6_LZ04 : -----------acgctgatata-t----ggat--gg-aatatttagtaca : 31
WS6_LZ04 : --------tcgaatggatcata-t----ggat--gg-aatatttagtaca : 34
WW6_LZ04 : ---------atcgaatgatcta-t----ggat--gg-aatatttagtaca : 33
YH6_LZ04 : ----------tgaatgatcata-t----ggat--gg-aatatttagtaca : 32
FT6_LZ04 : ----------tgatgtttcgta-t----ggat--gg-aatatttagtaca : 32
CH6_LZ04 : --------tcgaatgtatcata-t----ggat--gg-aatatttagtaca : 34
```

```
SH1_LZ04 : tgaaata-gttatttgctagatatataaatagatagataa-gatataact : 84
WS1_LZ04 : tgaaata-gttatttgctagatatataaatagatagataa-gatataact : 82
WW1_LZ04 : tgaaata-gttatttgctagatatataaatagatagataa-gatataact : 85
YH1_LZ04 : tgaaata-gttatttgctagatatataaatagatagataa-gatataact : 85
FT1_LZ04 : tgaaata-gttatttgctagatatataaatagatagataa-gatataact : 84
CH1_LZ04 : tgaaata-gttatttgctagatatataaatagatagataa-gatataact : 84
SH2_LZ04 : tgaaata-gttatttgctagatatataaatagatagataa-gatataact : 86
WS2_LZ04 : tgaaata-gttatttgctagatatataaatagatagataa-gatataact : 82
WW2_LZ04 : tgaaata-gttatttgctagatatataaatagatagataa-gatataact : 82
YH2_LZ04 : tgaaata-gttatttgctagatatataaatagatagataa-gatataact : 84
FT2_LZ04 : tgaaata-gttatttgctagatatataaatagatagataa-gatataact : 86
CH2_LZ04 : tgaaata-gttatttgctagatatataaatagatagataa-gatataact : 83
SH3_LZ04 : tgaaata-gttatttgctagatatataaatagatagataa-gatataact : 86
WS3_LZ04 : tgaaataggttatttgctagatatataaatagatagataa-gatataact : 91
WW3_LZ04 : tgaaata-gttatttgctagatatataaatagatagataa-gatataact : 85
YH3_LZ04 : tgaaata-gttatttgctagatatataaatagatagataa-gatataact : 84
FT3_LZ04 : tgaaata-gttatttgctagatatataaatagatagataa-gatataact : 84
CH3_LZ04 : tgaaata-gttatttgctagatatataaatagatagataa-gatataact : 81
SH4_LZ04 : tgaaata-gttatttgctagatatataaatagatagataa-gatataact : 83
WS4_LZ04 : tgaaata-gttatttgctagatatataaatagatagataa-gatataact : 82
WW4_LZ04 : tgaaata-gttatttgctagatatataaatagatagataa-gatataact : 82
YH4_LZ04 : tgaaata-gttatttgctagatatataaatagatagataa-gatataact : 83
FT4_LZ04 : tgaaata-gttatttgctagatatataaatagatagataa-gatataact : 84
CH4_LZ04 : tgaaata-gttatttgctagatatataaatagatagataa-gatataact : 82
SH5_LZ04 : tgaaata-gttatttgctagatatataaatagatagataa-gatataact : 89
WS5_LZ04 : tgaaata-gttatttgctagatatataaatagatagataattatataact : 86
WW5_LZ04 : tgaaata-gttatttgctagatatataaatagatagataa-gatataact : 84
YH5_LZ04 : tgaaata-gttatttgctagatatataaatagatagataa-gatataact : 87
FT5_LZ04 : tgaaata-gttatttgctagatatataaatagatagataa-gatataact : 90
CH5_LZ04 : tgaaata-gttatttgctagatatataaatagatagataa-gatataact : 86
SH6_LZ04 : tgaaata-gttatttgctagatatataaatagatagataa-gatataact : 79
WS6_LZ04 : tgaaata-gttatttgctagatatataaatagatagataa-gatataact : 82
WW6_LZ04 : tgaaata-gttatttgctagatatataaatagatagataa-gatataact : 81
YH6_LZ04 : tgaaata-gttatttgctagatatataaatagatagataa-gatataact : 80
FT6_LZ04 : tgaaata-gttatttgctagatatataaatagatagataa-gatataact : 80
CH6_LZ04 : tgaaata-gttatttgctagatatataaatagatagataa-gatataact : 82
```

```
SH1_LZ04 : aataaaataaataaaataaaaattgatcttgtattctggaattggaatca : 134
WS1_LZ04 : aataaaataaataaaataaaaattgatcttgtattctggaattggaatca : 132
WW1_LZ04 : aataaaataaataaaataaaaattgatcttgtattctggaattggaatca : 135
YH1_LZ04 : aataaaataaataaaataaaaattgatcttgtattctggaattggaatca : 135
FT1_LZ04 : aataaaataaataaaataaaaattgatcttgtattctggaattggaatca : 134
CH1_LZ04 : aataaaataaataaaataaaaattgatcttgtattctggaattggaatca : 134
SH2_LZ04 : aataaaataaataaaataaaaattgatcttgtattctggaattggaatca : 136
WS2_LZ04 : aataaaataaataaaataaaaattgatcttgtattctggaattggaatca : 132
WW2_LZ04 : aataaaataaataaaataaaaattgatcttgtattctggaattggaatca : 132
YH2_LZ04 : aataaaataaataaaataaaaattgatcttgtattctggaattggaatca : 134
FT2_LZ04 : aataaaataaataaaataaaaattgatcttgtattctggaattggaatca : 136
CH2_LZ04 : aataaaataaataaaataaaaattgatcttgtattctggaattggaatca : 133
SH3_LZ04 : aataaaataaataaaataaaaattgatcttgtattctggaattggaatca : 136
WS3_LZ04 : aataaaataaataaaataaaaattgatcttgtattctggaattggaatca : 141
WW3_LZ04 : aataaaataaataaaataaaaattgatcttgtattctggaattggaatca : 135
YH3_LZ04 : aataaaataaataaaataaaaattgatcttgtattctggaattggaatca : 134
FT3_LZ04 : aataaaataaataaaataaaaattgatcttgtattctggaattggaatca : 134
CH3_LZ04 : aataaaataaataaaataaaaattgatcttgtattctggaattggaatca : 131
SH4_LZ04 : aataaaataaataaaataaaaattgatcttgtattctggaattggaatca : 133
WS4_LZ04 : aataaaataaataaaataaaaattgatcttgtattctggaattggaatca : 132
WW4_LZ04 : aataaaataaataaaataaaaattgatcttgtattctggaattggaatca : 132
YH4_LZ04 : aataaaataaataaaataaaaattgatcttgtattctggaattggaatca : 133
FT4_LZ04 : aataaaataaataaaataaaaattgatcttgtattctggaattggaatca : 134
CH4_LZ04 : aataaaataaataaaataaaaattgatcttgtattctggaattggaatca : 132
SH5_LZ04 : aataaaataaataaaataaaaattgatcttgtattctggaattggaatca : 139
WS5_LZ04 : aataaaataaataaaataaaaattgatcttgtattctggaattggaatca : 136
WW5_LZ04 : aataaaataaataaaataaaaattgatcttgtattctggaattggaatca : 134
YH5_LZ04 : aataaaataaataaaataaaaattgatcttgtattctggaattggaatca : 137
FT5_LZ04 : aataaaataaataaaataaaaattgatcttgtattctggaattggaatca : 140
CH5_LZ04 : aataaaataaataaaataaaaattgatcttgtattctggaattggaatca : 136
SH6_LZ04 : aataaaataaataaaataaaaattgatcttgtattctggaattggaatca : 129
WS6_LZ04 : aataaaataaataaaataaaaattgatcttgtattctggaattggaatca : 132
WW6_LZ04 : aataaaataaataaaataaaaattgatcttgtattctggaattggaatca : 131
YH6_LZ04 : aataaaataaataaaataaaaattgatcttgtattctggaattggaatca : 130
FT6_LZ04 : aataaaataaataaaataaaaattgatcttgtattctggaattggaatca : 130
CH6_LZ04 : aataaaataaataaaataaaaattgatcttgtattctggaattggaatca : 132
```

```
SH1_LZ04 : aagaaagatatttaaaacggcgtgacttgtaataaatgtttctctgtaaa : 184
WS1_LZ04 : aagaaagatatttaaaacggcgtgacttgtaataaatgtttctctgtaaa : 182
WW1_LZ04 : aagaaagatatttaaaacggcgtgacttgtaataaatgtttctctgtaaa : 185
YH1_LZ04 : aagaaagatatttaaaacggcgtgacttgtaataaatgtttctctgtaaa : 185
FT1_LZ04 : aagaaagatatttaaaacggcgtgacttgtaataaatgtttctctgtaaa : 184
CH1_LZ04 : aagaaagatatttaaaacggcgtgacttgtaataaatgtttctctgtaaa : 184
SH2_LZ04 : aagaaagatatttaaaacggcgtgacttgtaataaatgtttctctgtaaa : 186
WS2_LZ04 : aagaaagatatttaaaacggcgtgacttgtaataaatgtttctctgtaaa : 182
WW2_LZ04 : aagaaagatatttaaaacggcgtgacttgtaataaatgtttctctgtaaa : 182
YH2_LZ04 : aagaaagatatttaaaacggcgtgacttgtaataaatgtttctctgtaaa : 184
FT2_LZ04 : aagaaagatatttaaaacggcgtgacttgtaataaatgtttctctgtaaa : 186
CH2_LZ04 : aagaaagatatttaaaacggcgtgacttgtaataaatgtttctctgtaaa : 183
SH3_LZ04 : aagaaagatatttaaaacggcgtgacttgtaataaatgtttctctgtaaa : 186
WS3_LZ04 : aagaaagatatttaaaacggcgtgacttgtaataaatgtttctctgtaaa : 191
WW3_LZ04 : aagaaagatatttaaaacggcgtgacttgtaataaatgtttctctgtaaa : 185
YH3_LZ04 : aagaaagatatttaaaacggcgtgacttgtaataaatgtttctctgtaaa : 184
FT3_LZ04 : aagaaagatatttaaaacggcgtgacttgtaataaatgtttctctgtaaa : 184
CH3_LZ04 : aagaaagatatttaaaacggcgtgacttgtaataaatgtttctctgtaaa : 181
SH4_LZ04 : aagaaagatatttaaaacggcgtgacttgtaataaatgtttctctgtaaa : 183
WS4_LZ04 : aagaaagatatttaaaacggcgtgacttgtaataaatgtttctctgtaaa : 182
WW4_LZ04 : aagaaagatatttaaaacggcgtgacttgtaataaatgtttctctgtaaa : 182
YH4_LZ04 : aagaaagatatttaaaacggcgtgacttgtaataaatgtttctctgtaaa : 183
FT4_LZ04 : aagaaagatatttaaaacggcgtgacttgtaataaatgtttctctgtaaa : 184
CH4_LZ04 : aagaaagatatttaaaacggcgtgacttgtaataaatgtttctctgtaaa : 182
SH5_LZ04 : aagaaagatatttaaaacggcgtgacttgtaataaatgtttctctgtaaa : 189
WS5_LZ04 : aagaaagatatttaaaacggcgtgacttgtaataaatgtttctctgtaaa : 186
WW5_LZ04 : aagaaagatatttaaaacggcgtgacttgtaataaatgtttctctgtaaa : 184
YH5_LZ04 : aagaaagatatttaaaacggcgtgacttgtaataaatgtttctctgtaaa : 187
FT5_LZ04 : aagaaagatatttaaaacggcgtgacttgtaataaatgtttctctgtaaa : 190
CH5_LZ04 : aagaaagatatttaaaacggcgtgacttgtaataaatgtttctctgtaaa : 186
SH6_LZ04 : aagaaagatatttaaaacggcgtgacttgtaataaatgtttctctgtaaa : 179
WS6_LZ04 : aagaaagatatttaaaacggcgtgacttgtaataaatgtttctctgtaaa : 182
WW6_LZ04 : aagaaagatatttaaaacggcgtgacttgtaataaatgtttctctgtaaa : 181
YH6_LZ04 : aagaaagatatttaaaacggcgtgacttgtaataaatgtttctctgtaaa : 180
FT6_LZ04 : aagaaagatatttaaaacggcgtgacttgtaataaatgtttctctgtaaa : 180
CH6_LZ04 : aagaaagatatttaaaacggcgtgacttgtaataaatgtttctctgtaaa : 182
```

```
SH1_LZ04 : ggaggggaatagtaatttattcattcctaatttattcctttcctatagaa : 234
WS1_LZ04 : ggaggggaatagtaatttattcattcctaatttattcctttcctatagaa : 232
WW1_LZ04 : ggaggggaatagtaatttattcattcctaatttattcctttcctatagaa : 235
YH1_LZ04 : ggaggggaatagtaatttattcattcctaatttattcctttcctatagaa : 235
FT1_LZ04 : ggaggggaatagtaatttattcattcctaatttattcctttcctatagaa : 234
CH1_LZ04 : ggaggggaatagtaatttattcattcctaatttattcctttcctatagaa : 234
SH2_LZ04 : ggaggggaatagtaatttattcattcctaatttattcctttcctatagaa : 236
WS2_LZ04 : ggaggggaatagtaatttattcattcctaatttattcctttcctatagaa : 232
WW2_LZ04 : ggaggggaatagtaatttattcattcctaatttattcctttcctatagaa : 232
YH2_LZ04 : ggaggggaatagtaatttattcattcctaatttattcctttcctatagaa : 234
FT2_LZ04 : ggaggggaatagtaatttattcattcctaatttattcctttcctatagaa : 236
CH2_LZ04 : ggaggggaatagtaatttattcattcctaatttattcctttcctatagaa : 233
SH3_LZ04 : ggaggggaatagtaatttattcattcctaatttattcctttcctatagaa : 236
WS3_LZ04 : ggaggggaatagtaatttattcattcctaatttattcctttcctatagaa : 241
WW3_LZ04 : ggaggggaatagtaatttattcattcctaatttattcctttcctatagaa : 235
YH3_LZ04 : ggaggggaatagtaatttattcattcctaatttattcctttcctatagaa : 234
FT3_LZ04 : ggaggggaatagtaatttattcattcctaatttattcctttcctatagaa : 234
CH3_LZ04 : ggaggggaatagtaatttattcattcctaatttattcctttcctatagaa : 231
SH4_LZ04 : ggaggggaatagtaatttattcattcctaatttattcctttcctatagaa : 233
WS4_LZ04 : ggaggggaatagtaatttattcattcctaatttattcctttcctatagaa : 232
WW4_LZ04 : ggaggggaatagtaatttattcattcctaatttattcctttcctatagaa : 232
YH4_LZ04 : ggaggggaatagtaatttattcattcctaatttattcctttcctatagaa : 233
FT4_LZ04 : ggaggggaatagtaatttattcattcctaatttattcctttcctatagaa : 234
CH4_LZ04 : ggaggggaatagtaatttattcattcctaatttattcctttcctatagaa : 232
SH5_LZ04 : ggaggggaatagtaatttattcattcctaatttattcctttcctatagaa : 239
WS5_LZ04 : ggaggggaatagtaatttattcattcctaatttattcctttcctatagaa : 236
WW5_LZ04 : ggaggggaatagtaatttattcattcctaatttattcctttcctatagaa : 234
YH5_LZ04 : ggaggggaatagtaatttattcattcctaatttattcctttcctatagaa : 237
FT5_LZ04 : ggaggggaatagtaatttattcattcctaatttattcctttcctatagaa : 240
CH5_LZ04 : ggaggggaatagtaatttattcattcctaatttattcctttcctatagaa : 236
SH6_LZ04 : ggaggggaatagtaatttattcattcctaatttattcctttcctatagaa : 229
WS6_LZ04 : ggaggggaatagtaatttattcattcctaatttattcctttcctatagaa : 232
WW6_LZ04 : ggaggggaatagtaatttattcattcctaatttattcctttcctatagaa : 231
YH6_LZ04 : ggaggggaatagtaatttattcattcctaatttattcctttcctatagaa : 230
FT6_LZ04 : ggaggggaatagtaatttattcattcctaatttattcctttcctatagaa : 230
CH6_LZ04 : ggaggggaatagtaatttattcattcctaatttattcctttcctatagaa : 232
```

```
SH1_LZ04 : catctaggaacaagaggatttaatcggatatatcgacagattcccgcgta : 284
WS1_LZ04 : catctaggaacaagaggatttaatcggatatatcgacagattcccgcgta : 282
WW1_LZ04 : catctaggaacaagaggattttatcggatatatcgacagattcccgcgta : 285
YH1_LZ04 : catctaggaacaagaggatttaatcggatatatcgacagattcccgcgta : 285
FT1_LZ04 : catctaggaacaagaggatttaatcggatatatcgacagattcccgcgta : 284
CH1_LZ04 : catctaggaacaagaggatttaatcggatatatcgacagattcccgcgta : 284
SH2_LZ04 : catctaggaacaagaggatttaatcggatatatcgacagattcccgcgta : 286
WS2_LZ04 : catctaggaacaagaggatttaatcggatatatcgacagattcccgcgta : 282
WW2_LZ04 : catctaggaacaagaggattttatcggatatatcgacagattcccgcgta : 282
YH2_LZ04 : catctaggaacaagaggatttaatcggatatatcgacagattcccgcgta : 284
FT2_LZ04 : catctaggaacaagaggatttaatcggatatatcgacagattcccgcgta : 286
CH2_LZ04 : catctaggaacaagaggatttaatcggatatatcgacagattcccgcgta : 283
SH3_LZ04 : catctaggaacaagaggatttaatcggatatatcgacagattcccgcgta : 286
WS3_LZ04 : catctaggaacaagaggatttaatcggatatatcgacagattcccgcgta : 291
WW3_LZ04 : catctaggaacaagaggattttatcggatatatcgacagattcccgcgta : 285
YH3_LZ04 : catctaggaacaagaggatttaatcggatatatcgacagattcccgcgta : 284
FT3_LZ04 : catctaggaacaagaggatttaatcggatatatcgacagattcccgcgta : 284
CH3_LZ04 : catctaggaacaagaggatttaatcggatataccgccagattcccgttta : 281
SH4_LZ04 : catctaggaacaagaggatttaatcggatatatcgacagattcccgcgta : 283
WS4_LZ04 : catctaggaacaagaggatttaatcggatatatcgacagattcccgcgta : 282
WW4_LZ04 : catctaggaacaagaggattttatcggatatatcgacagattcccgcgta : 282
YH4_LZ04 : catctaggaacaagaggatttaatcggatatatcgacagattcccgcgta : 283
FT4_LZ04 : catctaggaacaagaggatttaatcggatatatcgacagattcccgcgta : 284
CH4_LZ04 : catctaggaacaagaggatttaatcggatatatcgacagattcccgcgta : 282
SH5_LZ04 : catctaggaacaagaggatttaatcggatatatcgacagattcccgcgta : 289
WS5_LZ04 : catctaggaacaagaggatttaatcggatatatcgacagattcccgcgta : 286
WW5_LZ04 : catctaggaacaagaggattttatcggatatatcgacagattcccgcgta : 284
YH5_LZ04 : catctaggaacaagaggatttaatcggatatatcgacagattcccgcgta : 287
FT5_LZ04 : catctaggaacaagaggatttaatcggatatatcgacagattcccgcgta : 290
CH5_LZ04 : catctaggaacaagaggatttaatcggatatatcgacagattcccgcgta : 286
SH6_LZ04 : catctaggaacaagaggatttaatcggatatatcgacagattcccgcgta : 279
WS6_LZ04 : catctaggaacaagaggatttaatcggatatatcgacagattcccgcgta : 282
WW6_LZ04 : catctaggaacaagaggattttatcggatatatcgacagattcccgcgta : 281
YH6_LZ04 : catctaggaacaagaggatttaatcggatatatcgacagattcccgcgta : 280
FT6_LZ04 : catctaggaacaagaggatttaatcggatatatcgacagattcccgcgta : 280
CH6_LZ04 : catctaggaacaagaggatttaatcggatatatcgacagattcccgcgta : 282
```

```
SH1_LZ04 : gtccaaaa-- : 292
WS1_LZ04 : gtccaaaa-- : 290
WW1_LZ04 : gtccaaaa-- : 293
YH1_LZ04 : gtccaaaa-- : 293
FT1_LZ04 : gtccaaaa-- : 292
CH1_LZ04 : gtccaaaa-- : 292
SH2_LZ04 : gtccaaaa-- : 294
WS2_LZ04 : gtccaaaa-- : 290
WW2_LZ04 : gtccaaaa-- : 290
YH2_LZ04 : gtccaaaa-- : 292
FT2_LZ04 : gtccaaaa-- : 294
CH2_LZ04 : gtccaaaa-- : 291
SH3_LZ04 : gtccaaaa-- : 294
WS3_LZ04 : gtccaaaa-- : 299
WW3_LZ04 : gtccaaaa-- : 293
YH3_LZ04 : gtccaaaa-- : 292
FT3_LZ04 : gtccaaaa-- : 292
CH3_LZ04 : atccaaaatt : 291
SH4_LZ04 : gtccaaa--- : 290
WS4_LZ04 : gtccaaaa-- : 290
WW4_LZ04 : gtccaaaa-- : 290
YH4_LZ04 : gtccaaaa-- : 291
FT4_LZ04 : gtccaaaa-- : 292
CH4_LZ04 : gtccaaaa-- : 290
SH5_LZ04 : gtccaaaa-- : 297
WS5_LZ04 : gtccaaaa-- : 294
WW5_LZ04 : gtccaaaa-- : 292
YH5_LZ04 : gtccaaaa-- : 295
FT5_LZ04 : gtccaaaa-- : 298
CH5_LZ04 : gtccaaaa-- : 294
SH6_LZ04 : gtccaaa--- : 286
WS6_LZ04 : gtccaaaa-- : 290
WW6_LZ04 : gtccaaaa-- : 289
YH6_LZ04 : gtccaaaa-- : 288
FT6_LZ04 : gtccaaaa-- : 288
CH6_LZ04 : gtccaaaa-- : 290
```

Figure S24. The alignment of amplicons produced by designed LZ04 primer. The SNP regions are highlighted with red squares. The nucleotides identical across all plastomes are shaded in black, whereas those conserved in 60 % of the sequences are shaded in gray. SH: *P. salicina* 'Sanhua plum'; WS: *P. salicina* 'Wanshuang plum'; WW: *P. simonii* 'Weiwang'; YH: *P. salicina* 'Yinhong plum'; FT: *P. salicina* 'Fengtang plum' and CH: *P. salicina* 'Cuihong plum'. Arabic numerals represent different individuals.

```
SH1_LZ05 : ccccccaaaagtgtt-gattgaatcgtacca-gtctctactgggggttat : 48
WS1_LZ05 : -cgggggcaggtgtt-gattg-atcgtacca-gtctctactgggggttat : 46
WW1_LZ05 : -tgccggaaggtgtt-gattg-atcgtacca-gtctctactgggggttat : 46
YH1_LZ05 : --cgccggcagtgtt-gattg-atcgtacca-gtctctactgggggttat : 45
FT1_LZ05 : --tggcggaagtgtt-gattg-atcgtacca-gtctctactgggggttat : 45
CH1_LZ05 : cgccgggcaggtgat-gattg-atcgtacca-gtctctactgggggttat : 47
SH2_LZ05 : --cgccggaagtgtt-gattg-atcgtacca-gtctctactgggggttat : 45
WS2_LZ05 : ---gtggcaagtgtt-gattg-atcgtacca-gtctctactgggggttat : 44
WW2_LZ05 : ggcgggcccggtgttggattgaatcgtacca-gtctctactgggggttat : 49
YH2_LZ05 : --ggccgccagtgtt-gattg-atcgtacca-gtctctactgggggttat : 45
FT2_LZ05 : ---ctcggcagtgtt-gattg-atcgtacca-gtctctactgggggttat : 44
CH2_LZ05 : --cggggaaggtgtt-gattg-atcgtaccacgtctctactgggggttat : 46
SH3_LZ05 : gggggggaaggtgtt-gattg-atcgtacca-gtctctactgggggttat : 47
WS3_LZ05 : tggggggaaggtgtt-gattg-atcgtacca-gtctctactgggggttat : 47
WW3_LZ05 : cgccggcaaggtgtt-gattg-atcgtacca-gtctctactgggggttat : 47
YH3_LZ05 : ----ccggaagtgtt-gattg-atcgtacca-gtctctactgggggttat : 43
FT3_LZ05 : --ggcgagcggtgtt-gattg-atcgtacca-gtctctactgggggttat : 45
CH3_LZ05 : -tagcgggtcgtgtt-gattg-atcgtacca-gtctctactgggggttat : 46
SH4_LZ05 : --cggggaaagtgtt-gattg-atcgtacca-gtctctactgggggttat : 45
WS4_LZ05 : -ttggggaaagtgtt-gattg-atcgtacca-gtctctactgggggttat : 46
WW4_LZ05 : --gtgcgaaagtgtt-gattg-atcgtacca-gtctctactgggggttat : 45
YH4_LZ05 : -ctggatacagtgtt-gattg-atcgtacca-gtctctactgggggttat : 46
FT4_LZ05 : gtgcggtacagtgtt-gattg-atcgtacca-gtctctactgggggttat : 47
CH4_LZ05 : -gtgcggtcagtgtt-gattg-atcgtacca-gtctctactgggggttat : 46
SH5_LZ05 : ggcggcaactgtgtt-gattg-atcgtacca-gtctctactgggggttat : 47
WS5_LZ05 : ggggggaaaggtgtt-gattg-atcgtacca-gtctctactgggggttat : 47
WW5_LZ05 : -gcccaggacgtgtt-gattg-atcgtacca-gtctctactgggggttat : 46
YH5_LZ05 : tgcgaggactgtgtt-gattg-atcgtacca-gtctctactgggggttat : 47
FT5_LZ05 : --ggcggactgtgtt-gattg-atcgtacca-gtctctactgggggttat : 45
CH5_LZ05 : -ggccgcactgtgtt-gattg-atcgtacca-gtctctactgggggttat : 46
SH6_LZ05 : ---ctgcggcgtgtt-gattg-atcgtacca-gtctctactgggggttat : 44
532_LZ05 : ---gtgcgaagtgtt-gattg-atcgtacca-gtctctactgggggttat : 44
WW6_LZ05 : --gtgcggacgtgtt-gattg-atcgtacca-gtctctactgggggttat : 45
YH6_LZ05 : ---ttgcggcgtgtt-gattg-atcgtacca-gtctctactgggggttat : 44
FT6_LZ05 : ---ggcggaagtgtt-gattg-atcgtacca-gtctctactgggggttat : 44
CH6_LZ05 : ----cgcgatgtgtt-gattg-atcgtacca-gtctctactgggggttat : 43
```

```
SH1_LZ05 : tactcatttttgtacttgctgttttattttccaattatttcttta----- : 93
WS1_LZ05 : tactcatttttgtacttgctgttttattttccaattatttctttaattaa : 96
WW1_LZ05 : tactcatttttgtacttgctgttttattttccaattatttctttaattaa : 96
YH1_LZ05 : tactcatttttgtacttgctgttttattttccaattatttctttaattaa : 95
FT1_LZ05 : tactcatttttgtacttgctgttttattttccaattatttctttaattaa : 95
CH1_LZ05 : tactcatttttgtacttgctgttttattttccaattatttctttaattaa : 97
SH2_LZ05 : tactcatttttgtacttgctgttttattttccaattatttcttta----- : 90
WS2_LZ05 : tactcatttttgtacttgctgttttattttccaattatttctttaattaa : 94
WW2_LZ05 : tactcatttttgtacttgctgttttattttccaattatttctttaattaa : 99
YH2_LZ05 : tactcatttttgtacttgctgttttattttccaattatttctttaattaa : 95
FT2_LZ05 : tactcatttttgtacttgctgttttattttccaattatttctttaattaa : 94
CH2_LZ05 : tactcatttttgtacttgctgttttattttccaattatttctttaattaa : 96
SH3_LZ05 : tactcatttttgtacttgctgttttattttccaattatttcttta----- : 92
WS3_LZ05 : tactcatttttgtacttgctgttttattttccaattatttctttaattaa : 97
WW3_LZ05 : tactcatttttgtacttgctgttttattttccaattatttctttaattaa : 97
YH3_LZ05 : tactcatttttgtacttgctgttttattttccaattatttctttaattaa : 93
FT3_LZ05 : tactcatttttgtacttgctgttttattttccaattatttctttaattaa : 95
CH3_LZ05 : tactcatttttgtacttgctgttttattttccaattatttctttaattaa : 96
SH4_LZ05 : tactcatttttgtacttgctgttttattttccaattatttcttta----- : 90
WS4_LZ05 : tactcatttttgtacttgctgttttattttccaattatttctttaattaa : 96
WW4_LZ05 : tactcatttttgtacttgctgttttattttccaattatttctttaattaa : 95
YH4_LZ05 : tactcatttttgtacttgctgttttattttccaattatttctttaattaa : 96
FT4_LZ05 : tactcatttttgtacttgctgttttattttccaattatttctttaattaa : 97
CH4_LZ05 : tactcatttttgtacttgctgttttattttccaattatttctttaattaa : 96
SH5_LZ05 : tactcatttttgtacttgctgttttattttccaattatttcttta----- : 92
WS5_LZ05 : tactcatttttgtacttgctgttttattttccaattatttctttaattaa : 97
WW5_LZ05 : tactcatttttgtacttgctgttttattttccaattatttctttaattaa : 96
YH5_LZ05 : tactcatttttgtacttgctgttttattttccaattatttctttaattaa : 97
FT5_LZ05 : tactcatttttgtacttgctgttttattttccaattatttctttaattaa : 95
CH5_LZ05 : tactcatttttgtacttgctgttttattttccaattatttctttaattaa : 96
SH6_LZ05 : tactcatttttgtacttgctgttttattttccaattatttcttta----- : 89
532_LZ05 : tactcatttttgtacttgctgttttattttccaattatttctttaattaa : 94
WW6_LZ05 : tactcatttttgtacttgctgttttattttccaattatttctttaattaa : 95
YH6_LZ05 : tactcatttttgtacttgctgttttattttccaattatttctttaattaa : 94
FT6_LZ05 : tactcatttttgtacttgctgttttattttccaattatttctttaattaa : 94
CH6_LZ05 : tactcatttttgtacttgctgttttattttccaattatttctttaattaa : 93
```

```
SH1_LZ05 : -----------------------------attaaaaaaaagaatataaga : 114
WS1_LZ05 : aaaaaagaatataagaattctcttatcccattaaaaaaaagaatataaga : 146
WW1_LZ05 : aaaaaagaatataagaattctcttatcccattaaaaaaaagaatataaga : 146
YH1_LZ05 : aaaaaagaatataagaattctcttatcccattaaaaaaaagaatataaga : 145
FT1_LZ05 : aaaaaagaatataagaattctcttatcccattaaaaaaaagaatataaga : 145
CH1_LZ05 : aaaaaagaatataagaattctcttatcccattaaaaaaaagaatataaga : 147
SH2_LZ05 : -----------------------------attaaaaaaaagaatataaga : 111
WS2_LZ05 : aaaaaagaatataagaattctcttatcccattaaaaaaaagaatataaga : 144
WW2_LZ05 : aaaaaagaatataagaattctcttatcccattaaaaaaaagaatataaga : 149
YH2_LZ05 : aaaaaagaatataagaattctcttatcccattaaaaaaaagaatataaga : 145
FT2_LZ05 : aaaaaagaatataagaattctcttatcccattaaaaaaaagaatataaga : 144
CH2_LZ05 : aaaaaagaatataagaattctcttatcccattaaaaaaaagaatataaga : 146
SH3_LZ05 : -----------------------------attaaaaaaaagaatataaga : 113
WS3_LZ05 : aaaaaagaatataagaattctcttatcccattaaaaaaaagaatataaga : 147
WW3_LZ05 : aaaaaagaatataagaattctcttatcccattaaaaaaaagaatataaga : 147
YH3_LZ05 : aaaaaagaatataagaattctcttatcccattaaaaaaaagaatataaga : 143
FT3_LZ05 : aaaaaagaatataagaattctcttatcccattaaaaaaaagaatataaga : 145
CH3_LZ05 : aaaaaagaatataagaattctcttatcccattaaaaaaaagaatataaga : 146
SH4_LZ05 : -----------------------------attaaaaaaaagaatataaga : 111
WS4_LZ05 : aaaaaagaatataagaattctcttatcccattaaaaaaaagaatataaga : 146
WW4_LZ05 : aaaaaagaatataagaattctcttatcccattaaaaaaaagaatataaga : 145
YH4_LZ05 : aaaaaagaatataagaattctcttatcccattaaaaaaaagaatataaga : 146
FT4_LZ05 : aaaaaagaatataagaattctcttatcccattaaaaaaaagaatataaga : 147
CH4_LZ05 : aaaaaagaatataagaattctcttatcccattaaaaaaaagaatataaga : 146
SH5_LZ05 : -----------------------------attaaaaaaaagaatataaga : 113
WS5_LZ05 : aaaaaagaatataagaattctcttatcccattaaaaaaaagaatataaga : 147
WW5_LZ05 : aaaaaagaatataagaattctcttatcccattaaaaaaaagaatataaga : 146
YH5_LZ05 : aaaaaagaatataagaattctcttatcccattaaaaaaaagaatataaga : 147
FT5_LZ05 : aaaaaagaatataagaattctcttatcccattaaaaaaaagaatataaga : 145
CH5_LZ05 : aaaaaagaatataagaattctcttatcccattaaaaaaaagaatataaga : 146
SH6_LZ05 : -----------------------------attaaaaaaaagaatataaga : 110
532_LZ05 : aaaaaagaatataagaattctcttatcccattaaaaaaaagaatataaga : 144
WW6_LZ05 : aaaaaagaatataagaattctcttatcccattaaaaaaaagaatataaga : 145
YH6_LZ05 : aaaaaagaatataagaattctcttatcccattaaaaaaaagaatataaga : 144
FT6_LZ05 : aaaaaagaatataagaattctcttatcccattaaaaaaaagaatataaga : 144
CH6_LZ05 : aaaaaagaatataagaattctcttatcccattaaaaaaaagaatataaga : 143
```

```
SH1_LZ05 : attctcttatcccattcggaaagatatcatctcataattatctatggctg : 164
WS1_LZ05 : attctcttatcccattcggaaagatatcatctcataattatctatggctg : 196
WW1_LZ05 : attctcttatcccattcggaaagatatcatctcataattatctatggctg : 196
YH1_LZ05 : attctcttatcccattcggaaagatatcatctcataattatctatggctg : 195
FT1_LZ05 : attctcttatcccattcggaaagatatcatctcataattatctatggctg : 195
CH1_LZ05 : attctcttatcccattcggaaagatatcatctcataattatctatggctg : 197
SH2_LZ05 : attctcttatcccattcggaaagatatcatctcataattatctatggctg : 161
WS2_LZ05 : attctcttatcccattcggaaagatatcatctcataattatctatggctg : 194
WW2_LZ05 : attctcttatcccattcggaaagatatcatctcataattatctatggctg : 199
YH2_LZ05 : attctcttatcccattcggaaagatatcatctcataattatctatggctg : 195
FT2_LZ05 : attctcttatcccattcggaaagatatcatctcataattatctatggctg : 194
CH2_LZ05 : attctcttatcccattcggaaagatatcatctcataattatctatggctg : 196
SH3_LZ05 : attctcttatcccattcggaaagatatcatctcataattatctatggctg : 163
WS3_LZ05 : attctcttatcccattcggaaagatatcatctcataattatctatggctg : 197
WW3_LZ05 : attctcttatcccattcggaaagatatcatctcataattatctatggctg : 197
YH3_LZ05 : attctcttatcccattcggaaagatatcatctcataattatctatggctg : 193
FT3_LZ05 : attctcttatcccattcggaaagatatcatctcataattatctatggctg : 195
CH3_LZ05 : attctcttatcccattcggaaagatatcatctcataattatctatggctg : 196
SH4_LZ05 : attctcttatcccattcggaaagatatcatctcataattatctatggctg : 161
WS4_LZ05 : attctcttatcccattcggaaagatatcatctcataattatctatggctg : 196
WW4_LZ05 : attctcttatcccattcggaaagatatcatctcataattatctatggctg : 195
YH4_LZ05 : attctcttatcccattcggaaagatatcatctcataattatctatggctg : 196
FT4_LZ05 : attctcttatcccattcggaaagatatcatctcataattatctatggctg : 197
CH4_LZ05 : attctcttatcccattcggaaagatatcatctcataattatctatggctg : 196
SH5_LZ05 : attctcttatcccattcggaaagatatcatctcataattatctatggctg : 163
WS5_LZ05 : attctcttatcccattcggaaagatatcatctcataattatctatggctg : 197
WW5_LZ05 : attctcttatcccattcggaaagatatcatctcataattatctatggctg : 196
YH5_LZ05 : attctcttatcccattcggaaagatatcatctcataattatctatggctg : 197
FT5_LZ05 : attctcttatcccattcggaaagatatcatctcataattatctatggctg : 195
CH5_LZ05 : attctcttatcccattcggaaagatatcatctcataattatctatggctg : 196
SH6_LZ05 : attctcttatcccattcggaaagatatcatctcataattatctatggctg : 160
532_LZ05 : attctcttatcccattcggaaagatatcatctcataattatctatggctg : 194
WW6_LZ05 : attctcttatcccattcggaaagatatcatctcataattatctatggctg : 195
YH6_LZ05 : attctcttatcccattcggaaagatatcatctcataattatctatggctg : 194
FT6_LZ05 : attctcttatcccattcggaaagatatcatctcataattatctatggctg : 194
CH6_LZ05 : attctcttatcccattcggaaagatatcatctcataattatctatggctg : 193
```

```
SH1_LZ05 : tttatgtctcgagcatgaccacttgataaaatgtggaggaaagtaggaca : 214
WS1_LZ05 : tttatgtctcgagcatgaccacttgataaaatgtggaggaaagtaggaca : 246
WW1_LZ05 : tttatgtctcgagcatgaccacttgataaaatgtggaggaaagtaggac- : 245
YH1_LZ05 : tttatgtctcgagcatgaccacttgataaaatgtggaggaaagtaggaca : 245
FT1_LZ05 : tttatgtctcgagcatgaccacttgataaaatgtggaggaaagtaggaca : 245
CH1_LZ05 : tttatgtctcgagcatgaccacttgataaaatgtggaggaaagtaggaca : 247
SH2_LZ05 : tttatgtctcgagcatgaccacttgataaaatgtggaggaaagtaggaca : 211
WS2_LZ05 : tttatgtctcgagcatgaccacttgataaaatgtggaggaaagtaggac- : 243
WW2_LZ05 : tttatgtctcgagcatgaccacttgataaaatgtggaggaaagtaggaca : 249
YH2_LZ05 : tttatgtctcgagcatgaccacttgataaaatgtggaggaaagtaggac- : 244
FT2_LZ05 : tttatgtctcgagcatgaccacttgataaaatgtggaggaaagtaggac- : 243
CH2_LZ05 : tttatgtctcgagcatgaccacttgataaaatgtggaggaaagtaggac- : 245
SH3_LZ05 : tttatgtctcgagcatgaccacttgataaaatgtggaggaaagtaggaca : 213
WS3_LZ05 : tttatgtctcgagcatgaccacttgataaaatgtggaggaaagtaggac- : 246
WW3_LZ05 : tttatgtctcgagcatgaccacttgataaaatgtggaggaaagtaggaca : 247
YH3_LZ05 : tttatgtctcgagcatgaccacttgataaaatgtggaggaaagtaggac- : 242
FT3_LZ05 : tttatgtctcgagcatgaccacttgataaaatgtggaggaaagtaggac- : 244
CH3_LZ05 : tttatgtctcgagcatgaccacttgataaaatgtggaggaaagtaggac- : 245
SH4_LZ05 : tttatgtctcgagcatgaccacttgataaaatgtggaggaaagtaggaca : 211
WS4_LZ05 : tttatgtctcgagcatgaccacttgataaaatgtggaggaaagtaggac- : 245
WW4_LZ05 : tttatgtctcgagcatgaccacttgataaaatgtggaggaaagtaggaca : 245
YH4_LZ05 : tttatgtctcgagcatgaccacttgataaaatgtggaggaaagtaggac- : 245
FT4_LZ05 : tttatgtctcgagcatgaccacttgataaaatgtggaggaaagtaggac- : 246
CH4_LZ05 : tttatgtctcgagcatgaccacttgataaaatgtggaggaaagtaggac- : 245
SH5_LZ05 : tttatgtctcgagcatgaccacttgataaaatgtggaggaaagtaggaca : 213
WS5_LZ05 : tttatgtctcgagcatgaccacttgataaaatgtggaggaaagtaggac- : 246
WW5_LZ05 : tttatgtctcgagcatgaccacttgataaaatgtggaggaaagtaggaca : 246
YH5_LZ05 : tttatgtctcgagcatgaccacttgataaaatgtggaggaaagtaggac- : 246
FT5_LZ05 : tttatgtctcgagcatgaccacttgataaaatgtggaggaaagtaggac- : 244
CH5_LZ05 : tttatgtctcgagcatgaccacttgataaaatgtggaggaaagtaggac- : 245
SH6_LZ05 : tttatgtctcgagcatgaccacttgataaaatgtggaggaaagtaggaca : 210
532_LZ05 : tttatgtctcgagcatgaccacttgataaaatgtggaggaaagtaggac- : 243
WW6_LZ05 : tttatgtctcgagcatgaccacttgataaaatgtggaggaaagtaggaca : 245
YH6_LZ05 : tttatgtctcgagcatgaccacttgataaaatgtggaggaaagtaggac- : 243
FT6_LZ05 : tttatgtctcgagcatgaccacttgataaaatgtggaggaaagtaggaca : 244
CH6_LZ05 : tttatgtctcgagcatgaccacttgataaaatgtggaggaaagtaggaca : 243
```

```
SH1_LZ05 : aatggccgataca-------------------------------------- : 227
WS1_LZ05 : aatggccgataca-------------------------------------- : 259
WW1_LZ05 : aatggccgataca-------------------------------------- : 258
YH1_LZ05 : aatggccgataca-------------------------------------- : 258
FT1_LZ05 : aatggccgataca-------------------------------------- : 258
CH1_LZ05 : aatggccgataca-------------------------------------- : 260
SH2_LZ05 : aatggccgataca-------------------------------------- : 224
WS2_LZ05 : aatggccgataca-------------------------------------- : 256
WW2_LZ05 : aatggccgataca-------------------------------------- : 262
YH2_LZ05 : aatggccgataca-------------------------------------- : 257
FT2_LZ05 : aatggccgataca-------------------------------------- : 256
CH2_LZ05 : aatggccgataca-------------------------------------- : 258
SH3_LZ05 : aatggccgataca-------------------------------------- : 226
WS3_LZ05 : aatggccgataca-------------------------------------- : 259
WW3_LZ05 : aatggccgataca-------------------------------------- : 260
YH3_LZ05 : aatggccgataca-------------------------------------- : 255
FT3_LZ05 : aatggccgataca-------------------------------------- : 257
CH3_LZ05 : aatggccgataca-------------------------------------- : 258
SH4_LZ05 : aatggccgatacaagtaagcagccatagataattatgagatgatatcttt : 261
WS4_LZ05 : aatggccgatacaaataaacagccatagataattatgagatgatatcttt : 295
WW4_LZ05 : aatggccgataca-------------------------------------- : 258
YH4_LZ05 : aatggccgatacaaataaacagccatagataattatgagatgatatcttt : 295
FT4_LZ05 : aatggccgataca-------------------------------------- : 259
CH4_LZ05 : aatggccgataca-------------------------------------- : 258
SH5_LZ05 : aatggccgataca-------------------------------------- : 226
WS5_LZ05 : aatggccgataca-------------------------------------- : 259
WW5_LZ05 : aagggccgataca-------------------------------------- : 259
YH5_LZ05 : aatggccgataca-------------------------------------- : 259
FT5_LZ05 : aatggccgataca-------------------------------------- : 257
CH5_LZ05 : aatggccgataca-------------------------------------- : 258
SH6_LZ05 : aatggccgatacaatgt---------------------------------- : 227
532_LZ05 : aatggccgataca-------------------------------------- : 256
WW6_LZ05 : aatggccgataca-------------------------------------- : 258
YH6_LZ05 : aatggccgataca-------------------------------------- : 256
FT6_LZ05 : aatggccgataca-------------------------------------- : 257
CH6_LZ05 : aatggccgataca-------------------------------------- : 256
```

Figure S25. The alignment of amplicons produced by designed LZ05 primer. The Indel regions are highlighted with red squares. The nucleotides identical across all plastomes are shaded in black, whereas those conserved in 60 % of the sequences are shaded in gray. SH: *P. salicina* 'Sanhua plum'; WS: *P. salicina* 'Wanshuang plum'; WW: *P. simonii* 'Weiwang'; YH: *P. salicina* 'Yinhong plum'; FT: *P. salicina* 'Fengtang plum' and CH: *P. salicina* 'Cuihong plum'. Arabic numerals represent different individuals.

```
WS1_LZ06 : ---------cggattctgtatttgagcagttgttcgaattccttatgatc :  41
YH1_LZ06 : ------gcttctgatctgtatttgagcagttgttcgaattccttatgatc :  44
WS2_LZ06 : ----gcgattctgatctgtatttgagcagttgttcgaattccttatgatc :  46
YH2_LZ06 : ----gggattctgatctgtatttgagcagttgttcgaattccttatgatc :  46
WS3_LZ06 : -----gcttcttgatctgtatttgagcagttgttcgaattccttatgatc :  45
YH3_LZ06 : -----ggcttctgaactgtatttgagcagttgttcgaattccttatgatc :  45
WS4_LZ06 : ----cgcactctgatctgtatttgagcagttgttcgaattccttatgatc :  46
YH4_LZ06 : ----cggaatctgatctgtatttgagcagttgttcgaattccttatgatc :  46
WS5_LZ06 : --ttcgaaatctgaactgtatttgagcagttgttcgaattccttatgatc :  48
YH5_LZ06 : ----cgggatctgatctgtatttgagcagttgttcg-attccttatgatc :  45
WS6_LZ06 : ----ggcaatctgatctgtatttgagcagttgttcgaattccttatgatc :  46
YH6_LZ06 : ----gggactctgatctgtatttgagcagttgttcgaattccttatgatc :  46

WS1_LZ06 : tgcaactgaaacaagttcttgctaatggtaaaaaaggaactttgaatgta :  91
YH1_LZ06 : tgcaactgaaacaagttcttgctaatggtaaaaaaggagctttgaatgta :  94
WS2_LZ06 : tgcaactgaaacaagttcttgctaatggtaaaaaaggaactttgaatgta :  96
YH2_LZ06 : tgcaactgaaacaagttcttgctaatggtaaaaaaggagctttgaatgta :  96
WS3_LZ06 : tgcaactgaaacaagttcttgctaatggtaaaaaaggaactttgaatgta :  95
YH3_LZ06 : tgcaactgaaacaagttcttgctaatggtaaaaaaggagctttgaatgta :  95
WS4_LZ06 : tgcaactgaaacaagttcttgctaatggtaaaaaaggaactttgaatgta :  96
YH4_LZ06 : tgcaactgaaacaagttcttgctaatggtaaaaaaggagctttgaatgta :  96
WS5_LZ06 : tgcaactgaaacaagttcttgctaatggtaaaaaaggaactttgaatgta :  98
YH5_LZ06 : tgcaactgaaacaagttcttgctaatggtaaaaaaggagctttgaatgta :  95
WS6_LZ06 : tgcaactgaaacaagttcttgctaatggtaaaaaaggaactttgaatgta :  96
YH6_LZ06 : tgcaactgaaacaagttcttgctaatggtaaaaaaggagctttgaatgta :  96

WS1_LZ06 : ggggctgttcttattttacccgaggggtttgaattagcccctcccgatcg : 141
YH1_LZ06 : ggggctgttcttattttacccgaggggtttgaattagcccctcccgatcg : 144
WS2_LZ06 : ggggctgttcttattttacccgaggggtttgaattagcccctcccgatcg : 146
YH2_LZ06 : ggggctgttcttattttacccgaggggtttgaattagcccctcccgatcg : 146
WS3_LZ06 : ggggctgttcttattttacccgaggggtttgaattagcccctcccgatcg : 145
YH3_LZ06 : ggggctgttcttattttacccgaggggtttgaattagcccctcccgatcg : 145
WS4_LZ06 : ggggctgttcttattttacccgaggggtttgaattagcccctcccgatcg : 146
YH4_LZ06 : ggggctgttcttattttacccgaggggtttgaattagcccctcccgatcg : 146
WS5_LZ06 : ggggctgttcttattttacccgaggggtttgaattagcccctcccgatcg : 148
YH5_LZ06 : ggggctgttcttattttacccgaggggtttgaattagcccctcccgatcg : 145
WS6_LZ06 : ggggctgttcttattttacccgaggggtttgaattagcccctcccgatcg : 146
YH6_LZ06 : ggggctgttcttattttacccgaggggtttgaattagcccctcccgatcg : 146
```

```
WS1_LZ06 : tattttgcccgagattaaagaaaagataggaaatctgtcttttcagagct : 191
YH1_LZ06 : tattttgcccgagattaaagaaaagataggaaatctgtcttttcagagct : 194
WS2_LZ06 : tattttgcccgagattaaagaaaagataggaaatctgtcttttcagagct : 196
YH2_LZ06 : tattttgcccgagattaaagaaaagataggaaatctgtcttttcagagct : 196
WS3_LZ06 : tattttgcccgagattaaagaaaagataggaaatctgtcttttcagagct : 195
YH3_LZ06 : tattttgcccgagattaaagaaaagataggaaatctgtcttttcagagct : 195
WS4_LZ06 : tattttgcccgagattaaagaaaagataggaaatctgtcttttcagagct : 196
YH4_LZ06 : tattttgcccgagattaaagaaaagataggaaatctgtcttttcagagct : 196
WS5_LZ06 : tattttgcccgagattaaagaaaagataggaaatctgtcttttcagagct : 198
YH5_LZ06 : tattttgcccgagattaaagaaaagataggaaatctgtcttttcagagct : 195
WS6_LZ06 : tattttgcccgagattaaagaaaagataggaaatctgtcttttcagagct : 196
YH6_LZ06 : tattttgcccgagattaaagaaaagataggaaatctgtcttttcagagct : 196

WS1_LZ06 : atcgccccactaaaaaaaatattcttgtgataggtcctgttcctggta : 239
YH1_LZ06 : atcgccccactaaaaaaaatattcttgtgataggtcctgttcctggta : 242
WS2_LZ06 : atcgccccactaaaaaaaatattcttgtgataggtcctgttcctggt- : 243
YH2_LZ06 : atcgccccactaaaaaaaatattcttgtgataggtcctgttcctggt- : 243
WS3_LZ06 : atcgccccactaaaaaaaatattcttgtgataggtcctgttcctggt- : 242
YH3_LZ06 : atcgccccactaaaaaaaatattcttgtgataggtcctgttcctggt- : 242
WS4_LZ06 : atcgccccactaaaaaaaatattcttgtgataggtcctgttcctggta : 244
YH4_LZ06 : atcgccccactaaaaaaaatattcttgtgataggtcctgttcctggt- : 243
WS5_LZ06 : atcgccccactaaaaaaaatattcttgtgataggtcctgttcctggta : 246
YH5_LZ06 : atcgccccactaaaaaaaatattcttgtgataggtcctgttcctggta : 243
WS6_LZ06 : atcgccccactaaaaaaaatattcttgtgataggtcctgttcctggta : 244
YH6_LZ06 : atcgccccactaaaaaaaatattcttgtgataggt-ctgttcctggt- : 242
```

Figure S26. The alignment of amplicons produced by designed LZ06 primer. The SNP regions are highlighted with red squares. The nucleotides identical across all plastomes are shaded in black, whereas those conserved in 60 % of the sequences are shaded in gray. WS: *P. salicina* 'Wanshuang plum' and YH: *P. salicina* 'Yinhong plum'. Arabic numerals represent different individuals.

```
WY1_LZ07 : gggggggcagcatcgagt-ctcgacgaactcgaagaggtttttcttctct :  49
GF1_LZ07 : gagg------ctatcagt-ctcgac--actcgaagaggtttttcttctct :  41
WY2_LZ07 : gtgggcg----atcgagt-ctcgacg-actcgaagaggtttttcttctct :  44
GF2_LZ07 : gtgg--gcataatcgagt-ctcgacg-actcgaagaggtttttcttctct :  46
WY3_LZ07 : gaagcg-----atcgagt-ctcgacg-actcg-agaggtttttcttctct :  42
GF3_LZ07 : gggggcgcgttaacgagt-ctcgacg-actcgaagaggtttttcttctct :  48
WY4_LZ07 : -gga------cgatcagt-ctcgacg-actcgaagaggtttttcttctct :  41
GF4_LZ07 : -gcg------gaatcagt-ctcgacg-actcgaagaggtttttcttctct :  41
WY5_LZ07 : tgga------cgatcagt-ctcgacg-actcgaagaggtttttcttctct :  42
GF5_LZ07 : -gc--------tatcagt-ctcgacg-actcgaagaggtttttcttctct :  39
WY6_LZ07 : ---g------cgatcagt-ctcgacg-actcgaagaggtttttcttctct :  39
GF6_LZ07 : ----------gaatgggtcctcgacg-actcgaagaggtttttcttctct :  39

WY1_LZ07 : ccctgtttgtttctccagaaaaaagaaatgctttcgtgaaaaatctgatt :  99
GF1_LZ07 : ccctgtttgtttctcgagaaaaaagaaatgctttcg---------tgatt :  82
WY2_LZ07 : ccctgtttgtttctcca-aaaaaagaaatgctttcgtgaaaaatctgatt :  93
GF2_LZ07 : ccctgtttgtttctcgagaaaaaagaaatgttttcg---------tgatt :  87
WY3_LZ07 : ccctgtttgtttctcca--aaaaaaaaatgctttcgtgaaaaatctgatt :  90
GF3_LZ07 : ccctgtttgtttctcgagaaaaaagaaatgctttcg---------tgatt :  89
WY4_LZ07 : ccctgtttgtttctc-agaaaaaagaaatgttttcgtgaaaaatctgatt :  90
GF4_LZ07 : ccctgtttgtttctcgagaaaaaagaaatgttttcg---------tgatt :  82
WY5_LZ07 : ccctgtttgtttctcgagaaaaaagaaatgctttcgtgaaaaatctgatt :  92
GF5_LZ07 : ccctgtttgtttctcgagaaaaaagaaatgttttcg---------tgatt :  80
WY6_LZ07 : ccctgtttgtttctcgagaaaaaagaaatgctttcgtgaaaaatctgatt :  89
GF6_LZ07 : ccctgtttgtttctcgagaaaaaagaaatgttttcg---------tgatt :  80

WY1_LZ07 : atttctgttcttttcgtttcaggctctgctgttatcgaattttggtcca : 149
GF1_LZ07 : atttctgttattttcggtttcatgctctgctgct-tcggattttggtcca : 131
WY2_LZ07 : atttctgttcttttcgtttcaggctctgctgttatcgaattttggtcca : 143
GF2_LZ07 : atttctgttattttcggtttcatgctctgctgct-tcggattttggtcca : 136
WY3_LZ07 : atttctgttcttttcgtttcaggctctgctgttatcggattttggtcca : 140
GF3_LZ07 : atttctgttattttcggtttcatgctctgctgct-tcggattttggtcca : 138
WY4_LZ07 : atttctgtttttttcgtttcaggctctgctggtatcggattttggtcca : 140
GF4_LZ07 : atttctgttgttttcggtttcttgctctgctggtatcggattttggtcca : 132
WY5_LZ07 : atttctgttgttttcggtttcaggctctgctggtatcggattttggtcca : 142
GF5_LZ07 : atttctgttgttttcggtttcttgctctgctggtatcggattttggtcca : 130
WY6_LZ07 : atttctgttgttttcggtttcaggctctgctggtatcggattttggtcca : 139
GF6_LZ07 : atttctgttgttttcggtttctggctctgct-gcatcggattttggtcca : 129

WY1_LZ07 : aaaatcacaattaaaattgtgaaaagtttgagggaggacatatttgctgg : 199
GF1_LZ07 : aaaatcacaattaacattgtggaaagtttgaaggaggacatagatgctgg : 181
WY2_LZ07 : aaaatcacaattaaaattgtgaaaagtttgagggaggacatagttgctgg : 193
GF2_LZ07 : aaaatcacaattaacattgtggaaatttttgaaggaggacatagatgctgg : 186
```

```
WY3_LZ07 : aaaatcacaattaaaattgtgaaaagtttgagggaggacatagttgctgg : 190
GF3_LZ07 : aaaatcacaattaacattgtggaaattttgaaggaggacatagatgctgg : 188
WY4_LZ07 : aaaatcacaattaaaattgtggaaagtttgagggaggacatagttgctgg : 190
GF4_LZ07 : aaaatcacaattaacattgtggaaagtttgagggaggacatagttgctgg : 182
WY5_LZ07 : aaaatcacaattaaaattgtgaaaagtttgagggaggacatagttgctgg : 192
GF5_LZ07 : taaatcacaattaacattgtggaaagtttgaaggaggacatggttgctgg : 180
WY6_LZ07 : aaaatcacaattaaaattgtggaaagtttgagggaggacatagttgctgg : 189
GF6_LZ07 : aaaatcacaattaacattgtggaaagtttgaaggaggacatagttgctgg : 179

WY1_LZ07 : aaacctaaaatctgaaaccgaaataatggtaatgttaacaattgaaaagc : 249
GF1_LZ07 : aaagcttaaatctgtaaactaaataaaggtaatgttaacaattgaaaaac : 231
WY2_LZ07 : aaacctaaaatctgaaaccgaaataaaggcaatgttaacaattgaaaagc : 243
GF2_LZ07 : aaagcttaaatctgtaaactaaataaaggtaatgttaacaattgaaaaac : 236
WY3_LZ07 : aaacctaaaatctgaaaccgaaataatggtaatgttaacaattgaaaagc : 240
GF3_LZ07 : aaagcttaaatctgtaaactaaataaaggaaatgttaacaattgaaaaac : 238
WY4_LZ07 : gaagctgaaatctggaagcgaaataaaggtaatgttaacaattgaaaagc : 240
GF4_LZ07 : aaagctgaaatctggaagcgaaataaaggtaatgttaacaattgaaaagc : 232
WY5_LZ07 : aaagctaaaatctggaaccgaaataatggtaatgttaacaattgaaaagc : 242
GF5_LZ07 : aaagctgaaatctggaagctaaataaaggtaatgttgacaattgaaaagc : 230
WY6_LZ07 : gaagctgaaatctggaagcgaaataatggtaatgttaacaattgaaaagc : 239
GF6_LZ07 : aaagctgaaatctggaagctaaataaaggtaatgttaacaattgaaaagc : 229

WY1_LZ07 : cttttatgttcatagcaacatta-ccatcattacctttattgcgcttcgg : 298
GF1_LZ07 : cttttatgttcataacaacattg-gcaacattacctttattacgcttcta : 280
WY2_LZ07 : cttttatgttcatagcaacatta-ccaccattacgtttattgcgcttcgg : 292
GF2_LZ07 : cttttatgtccataacaacattg-gcaacattacctttattacgcttcta : 285
WY3_LZ07 : cttttatgttcataccaacatta-ccaccattacctttattgcgcttcgg : 289
GF3_LZ07 : cttttatgtccataacaacattg-gcaacattacctttattacgcttcta : 287
WY4_LZ07 : cttttatgttcttagcaacattg-gcagcatta----------------- : 272
GF4_LZ07 : cttttatgttcatagcaacattg-gcagcattagcagcatta-------- : 273
WY5_LZ07 : cttttatgttcttagcaacattg-gcagcatta----------------- : 274
GF5_LZ07 : cttttatgttcatagcaacattgagcagcattagcagcatta-------- : 272
WY6_LZ07 : cttttatgttcatagcaacattg-gcagcatta----------------- : 271
GF6_LZ07 : cttttatgttcatagcaacattg-gcagcattagcagcattacg------ : 272
```

```
WY1_LZ07 : gttttcggcttcccggcaactatgtccccccccaaactttccacaatctt : 348
GF1_LZ07 : gatttcagcgttccaggaactatggcctccctgaaactttccacaatctt : 330
WY2_LZ07 : gatttcggcttcccggcaactatgtcctcccccaaactttccacaatctt : 342
GF2_LZ07 : gatttcagcgttccaggaactatggcctccctcaaactttccacaatctt : 335
WY3_LZ07 : gatttcggcttcccggcaactatgtccccccccaaactttccacaatctt : 339
GF3_LZ07 : gatttcagcgttccaggaactatggcctccctcaaactttccacaatctt : 337
WY4_LZ07 : -------------------------------------------------- :  -
GF4_LZ07 : -------------------------------------------------- :  -
WY5_LZ07 : -------------------------------------------------- :  -
GF5_LZ07 : -------------------------------------------------- :  -
WY6_LZ07 : -------------------------------------------------- :  -
GF6_LZ07 : -------------------------------------------------- :  -

WY1_LZ07 : atttgtgatttttggacaaaaatgggataccggcagagccagaaaccgaa : 398
GF1_LZ07 : aattgtgatttttgggccaaaatccgataccagcggagcctgaagcggaa : 380
WY2_LZ07 : atttgtgatttttggacaaaaatgcgataccggcagagcctgaaaccgaa : 392
GF2_LZ07 : aattgtgatttttggaccaaaatccgataccagcggagcctgaagcggaa : 385
WY3_LZ07 : atttgtgatttttggaccaaaatgcgataccggcagagcctgaaaccgaa : 389
GF3_LZ07 : aattgtgatttttgggccaaaatccgataccagcggagcctgaagcggaa : 387
WY4_LZ07 : -------------------------------------------------- :  -
GF4_LZ07 : -------------------------------------------------- :  -
WY5_LZ07 : -------------------------------------------------- :  -
GF5_LZ07 : -------------------------------------------------- :  -
WY6_LZ07 : -------------------------------------------------- :  -
GF6_LZ07 : -------------------------------------------------- :  -

WY1_LZ07 : aagaacagaaataatcagattttccacgaaggcatttcttttttcgcgag : 448
GF1_LZ07 : aacaacagaaataat---------cgcgaaaacatttcttttttcgcgag : 421
WY2_LZ07 : aagaacagaaataatcagattttccacgaaggcatttcttttttcgcgag : 442
GF2_LZ07 : aacaacagaaagaat---------cgcgaaaacatttcttttttcgcgag : 426
WY3_LZ07 : aagaacagaaataatcagattttccacgaaagcatttcttttttcgcgag : 439
GF3_LZ07 : aacaacagaaataat---------cgcgaaaacatttcttttttcgcgag : 428
WY4_LZ07 : -------------------------------------------------- :  -
GF4_LZ07 : -------------------------------------------------- :  -
WY5_LZ07 : -------------------------------------------------- :  -
GF5_LZ07 : -------------------------------------------------- :  -
WY6_LZ07 : -------------------------------------------------- :  -
GF6_LZ07 : -------------------------------------------------- :  -
```

```
WY1_LZ07 : aaacaaacagggagagaagaaaaacctcttcgagtgcgtcggggactcga : 498
GF1_LZ07 : aaacaaacagggagagaagaaaaacctgttcgagttcgtcgaggactcga : 471
WY2_LZ07 : aaacaaacagggagagaagaaaaacctcttcgagtgcgtcggggactcga : 492
GF2_LZ07 : aaacaaacagggagagaagaaaaacctgttcgagttggtcgaggactcga : 476
WY3_LZ07 : aaacaaacagggagagaagaaaaacctcttcgagtgcgtcggggactcga : 489
GF3_LZ07 : aaacaaacagggagagaagaaaaacctgttcgagttggtcgaggactcga : 478
WY4_LZ07 : -------------------------------------------------- :   -
GF4_LZ07 : -------------------------------------------------- :   -
WY5_LZ07 : -------------------------------------------------- :   -
GF5_LZ07 : -------------------------------------------------- :   -
WY6_LZ07 : -------------------------------------------------- :   -
GF6_LZ07 : -------------------------------------------------- :   -

WY1_LZ07 : tgagtgtgagcgagattccagtaga-----------------ggcaagag : 531
GF1_LZ07 : tcagtgtcagcgagattccaggaaaccaagagcatccagta-agcaagag : 520
WY2_LZ07 : tcagtgtgagcgagattccagtaga-----------------ggcaagag : 525
GF2_LZ07 : tgagtgtcagggagattccaggagggcgagagcatccagtagagcaagag : 526
WY3_LZ07 : tcagtgacagcgagattccagtaga------------------gcaagag : 521
GF3_LZ07 : tgagtgtcagggagattccaggagagcgagagcatccagtagagcaagag : 528
WY4_LZ07 : -------------------------------------------------- :   -
GF4_LZ07 : -------------------------------------------------- :   -
WY5_LZ07 : -------------------------------------------------- :   -
GF5_LZ07 : -------------------------------------------------- :   -
WY6_LZ07 : -------------------------------------------------- :   -
GF6_LZ07 : -------------------------------------------------- :   -

WY1_LZ07 : ca : 533
GF1_LZ07 : ca : 522
WY2_LZ07 : ca : 527
GF2_LZ07 : ca : 528
WY3_LZ07 : ca : 523
GF3_LZ07 : ca : 530
WY4_LZ07 : -- :   -
GF4_LZ07 : -- :   -
WY5_LZ07 : -- :   -
GF5_LZ07 : -- :   -
WY6_LZ07 : -- :   -
GF6_LZ07 : -- :   -
```

Figure S27. The alignment of amplicons produced by designed LZ07 primer. The Indel regions are highlighted with red squares. The nucleotides identical across all plastomes are shaded in black, whereas those conserved in 60 % of the sequences are shaded in gray. WY: *P. salicina* 'Wuyuecui' and GF: *P. salicina* 'No.2 Guofeng'. Arabic numerals represent different individuals.

```
RE1_LZ08 : actgtggttg--tttaatg-agtaatggagaacta-taatgttaatttaa :  46
BT1_LZ08 : atggtg-ttg--aataatg-agtaatggagaactg-taatgttaatttaa :  45
RE2_LZ08 : gtggtgtttgcatataatg-agtaatggagaactagtaatgttaatttaa :  49
BT2_LZ08 : atggcggtttgaataaatg-agtaatggagaactg-taatgttaatttaa :  48
RE3_LZ08 : gcgtcttt----cattatg-agtaatggagaacta-taatgttaatttaa :  44
BT3_LZ08 : atggtgtttg--ccttatg-agtaatggagaactg-taatgttaatttaa :  46
RE4_LZ08 : gggtctttgt--tataatg-agtaatggagaactagtaatgttaatttaa :  47
BT4_LZ08 : gggttctttgc-aataatg-agtaatggagaactg-taatgttaatttaa :  47
RE5_LZ08 : atattccttgtatataatgagtaaatggagaacta-taatgttaatttaa :  49
BT5_LZ08 : g-gatctttgcatataatgaagtaatggagaacta-taatgttaatttaa :  48
RE6_LZ08 : agggtcttgc--tataatg-agtaatggagaactagtaatgttaatttaa :  47
BT6_LZ08 : gggc---ttgcaataaatg-agtaatggagaactg-taatgttaatttaa :  45

RE1_LZ08 : tccaaagaatataatgcagctatcaattgatgcttcatttgagagttatg :  96
BT1_LZ08 : tccaaagaatataatgcagctatcaattgatgcttcatttgagagtta-- :  93
RE2_LZ08 : tccaaagaatataatgcagctatcaattgatgcttcatttgagagttatg :  99
BT2_LZ08 : tccaaagaatataatgcagctatcaattgatgcttcatttgagagtta-- :  96
RE3_LZ08 : tccaaagaatataatgcagctatcaattgatgcttcatttgagagttatg :  94
BT3_LZ08 : tccaaagaatataatgcagctatcaattgatgcttcatttgagagtta-- :  94
RE4_LZ08 : tccaaagaatataatgcagctatcaattgatgcttcatttgagagttatt :  97
BT4_LZ08 : tccaaagaatataatgcagctatcaattgatgcttcatttgagagtta-- :  95
RE5_LZ08 : tccaaagaatataatgcagctatcaattgatgcttcatttgagagttatt :  99
BT5_LZ08 : tccaaagaatataatgcagctatcaattgatgcttcatttgagagttatg :  98
RE6_LZ08 : tccaaagaatataatgcagctatcaattgatgcttcatttgagagttatg :  97
BT6_LZ08 : tccaaagaatataatgcagctatcaattgatgcttcatttgagagtta-- :  93

RE1_LZ08 : gtgtttgataaaaattcctttatacaaacgggcacgatgtggtataggtg : 146
BT1_LZ08 : atgtttgataaaaattcctttatacaaacaggtacgagctggtatagttg : 143
RE2_LZ08 : gtgtttgataaaatccctttatacaaacgggcacgatgtgatataggtg : 149
BT2_LZ08 : atgtttgataaaaattcctttatacaaacaggtacgagctggtatagttg : 146
RE3_LZ08 : gtgtttgataaaaatccctttatacaaacgggcacgatgtggtatatgtg : 144
BT3_LZ08 : atgtttgataaaaattcctttatacaaacaggtacgagctggtatagttg : 144
RE4_LZ08 : gtgttagataaaaattcctttatacaaacgggcacgatgtgatatatgtg : 147
BT4_LZ08 : atgtttgataaaaattcctttatacaaacaggtacgagctggtatagttg : 145
RE5_LZ08 : gtgttagataaaaatccctttatacaaacgggcacgatgtggtatatgtg : 149
BT5_LZ08 : atgtttgataaaaattcctttatacaaacaggtacgagctggtatagttg : 148
RE6_LZ08 : gtgttagataaaaatccctttatacaaacgggcacgatgtggtatatgtg : 147
BT6_LZ08 : atgtttgataaaaattcctttatacaaacaggtacgagctggtatagttg : 143
```

```
RE1_LZ08 : gatatccaagtggtgagaaatcatctttgattacccgtgtgctgaagcca : 196
BT1_LZ08 : gatatccaaatgttgggaaatcatctttgattaaccgttgctgaagcga : 193
RE2_LZ08 : tatatccaagtggtgagaaatcctctttgattacccgtttggtgaagcca : 199
BT2_LZ08 : gatatccaaatgttgggaaatcatctttgattaaccgttgctgaagcga : 196
RE3_LZ08 : tatatccaagtggtgaaaaatcctctttttattacccgtttggtgaagaca : 194
BT3_LZ08 : gatatccaaatgttgggaaatcatctttgattaaccgttgctgaagcga : 194
RE4_LZ08 : tatatccaagtggggagaaatcctctttttattacccgtgtgctgaagaca : 197
BT4_LZ08 : gatatccaaatgttgggaaatcatctttgattaaccgttgctgaagcga : 195
RE5_LZ08 : tatatccaaatgttgagaaatcatctttttattacccgtgtgctgaagaga : 199
BT5_LZ08 : gatatccaaatgttgggaaatcatctttgattaaccgttgctgaagcga : 198
RE6_LZ08 : tatatccaagtggtgaaaaatcattttttattcaccgtgtggtgaagaga : 197
BT6_LZ08 : gatatccaaatgttgggaaatcatctttgattaaccgttgctgaagcga : 193

RE1_LZ08 : caagtga- : 203
BT1_LZ08 : cgaatgag : 201
RE2_LZ08 : caagtga- : 206
BT2_LZ08 : cgaatgag : 204
RE3_LZ08 : caagtga- : 201
BT3_LZ08 : cgaatgag : 202
RE4_LZ08 : caagtga- : 204
BT4_LZ08 : cgaatgag : 203
RE5_LZ08 : aaagtga- : 206
BT5_LZ08 : cgaatga- : 205
RE6_LZ08 : caagtga- : 204
BT6_LZ08 : cgaatgag : 201
```

Figure S28. The alignment of amplicons produced by designed LZ08 primer. The Indel regions are highlighted with red squares. The nucleotides identical across all plastomes are shaded in black, whereas those conserved in 60 % of the sequences are shaded in gray. RE: *P. domestica* 'Richard Early' and BT: *P. domestica* 'Bingtang plum'. Arabic numerals represent different individuals.
